# Pushing vs Pulling: The Unique Geometry of Mechanophore Activation in a Rotaxane Force Actuator

**DOI:** 10.1021/jacs.4c05168

**Published:** 2024-06-07

**Authors:** Lei Chen, Guillaume De Bo

**Affiliations:** Department of Chemistry, University of Manchester, Oxford Road, Manchester, M13 9PL, United Kingdom

## Abstract

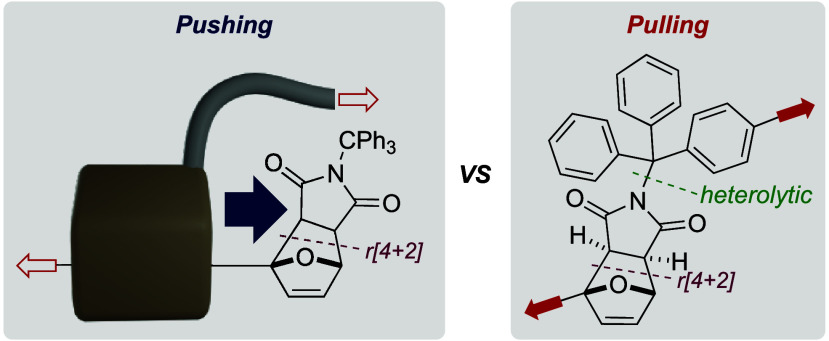

Mechanophores (mechanosensitive molecules) are usually activated by pulling them with covalently attached polymers. A rotaxane actuator offers a new geometry of activation as the macrocycle pushes against a stoppering mechanophore. Here we compare both *pulling* and *pushing* activations and show that pushing is more efficient and selective than pulling. We found that the pulling activation of a bulky furan/maleimide adduct occurs via two competing dissociation pathways: retrocycloaddition and heterolytic cleavage (generating a trityl cation in the process), while the same adduct only cleaves by retrocycloaddition during pushing activation. These results further demonstrate the efficacy and versatility of rotaxane actuators.

In polymer mechanochemistry, mechanophores are usually activated with covalently attached polymers *pulling* on the structure.^1^ The resulting tensile deformation ultimately leads to the scission of at least one bond within the mechanophore structure. The vast majority of the mechanophores described to date have been actuated in this way.^2^ Alternative actuation mechanisms exist such as the flex-activation,^3^ where molecular deformation leads to the activation of a bond orthogonal to the elongation vector, and more recently by the action of a mechanical bond.^4−9^ Rotaxanes are particularly useful, as the macrocycle can easily move along its axle^10,11^ and can impart substantial local deformation. Indeed, we have shown that a rotaxane actuator can influence the mechanochemical reactivity of a mechanophore embedded in its axle, and promote unstoppering reactions by enhancing the mechanical lability of covalent bonds in the axle.^7,8^ More recently, we have demonstrated the use of such a rotaxane actuator for the force-controlled release of up to 5 cargo molecules dispersed along its axle.^9^ The iterative activation of these mechanophores was made possible by the unique *pushing* actuation geometry provided by the rotaxane architecture, in which the macrocycle *pushes* against the mechanophore until activation occurs. Here we compare the *pushing* and *pulling* activation of a Diels–Alder adduct mechanophore and show that *pushing* activation is more efficient and selective than *pulling*. In fact, the activation by *pulling* leads to 2 competing dissociation pathways:^12,13^ the expected retrocycloaddition, also observed during *pushing*, and the heterolytic scission of a C–N bond that leads to the formation of a trityl cation.^9,14^ These results further demonstrate the power of the mechanical bond in mechanochemistry.

We compared the reactivity of a Diels–Alder mechanophore (Figure 1), the *proximal*-*exo* isomer of a bulky furan/maleimide adduct,^15^ actuated by the intermediacy of a rotaxane (**1**, *pushing*) or by the direct action of the polymer arms (**4**, *pulling*). Though these macromolecules are elongated in both cases, from the mechanophore’s perspective, the activation occurs by *pushing* or *pulling*. That is to say that in the rotaxane, the mechanophore is activated by being *pushed* by the macrocycle, while in the *pulling* activation it is being *pulled* by both polymer arms (Figure 1). The main difference resides in the fact that *pushing* is more diffuse, as a substantial portion of the exposed surface of the mechanophore is in contact with the advancing macrocycle. In contrast, in the *pulled* mechanophore, tension propagates across its framework from one anchorage point to another, leading to more pronounced deformation around the most compliant bonds.

**Figure 1:**
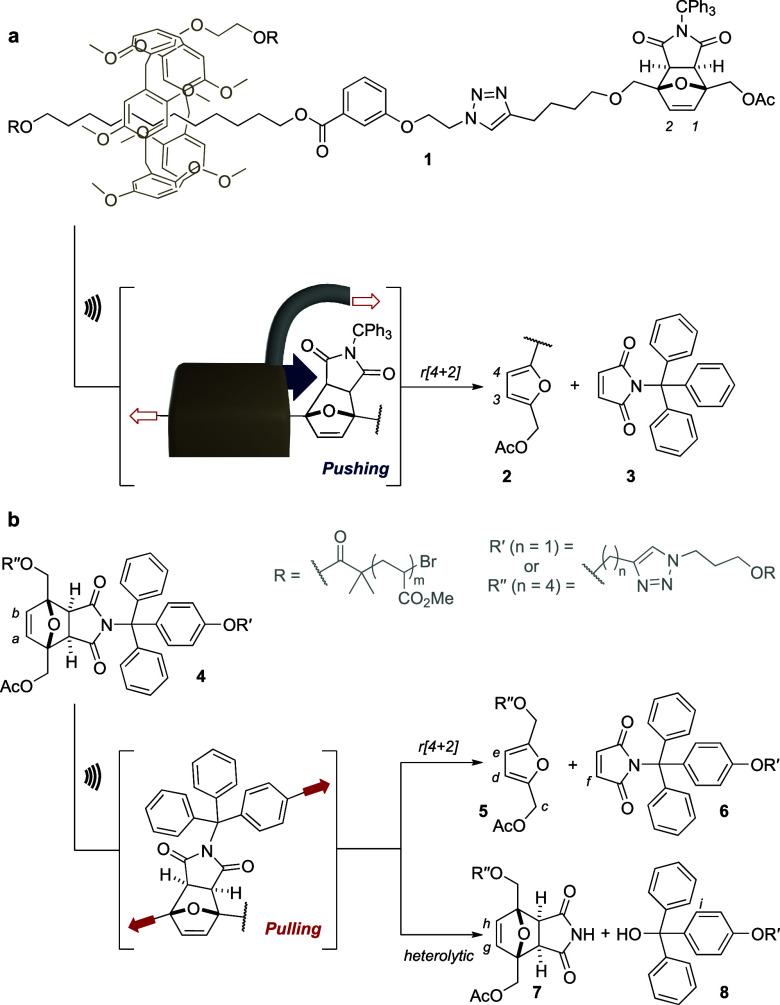
*Pushing* vs *pulling* actuation for the activation of a Diels–Alder mechanophore. (a) *Pushing* actuation leads to the release of N-triphenylmethyl maleimide **3** via a formal retro-[4 + 2] cycloaddition in rotaxane **1**. (b) *Pulling* actuation gives rise to two competing dissociations in mechanophore **4**: r[4 + 2] or heterolytic scission at the C–N bond. Conditions: US (20 kHz, 13.0 W/cm^2^, 1s ON/1s OFF), THF/H_2_O: 75/1, 5–10 °C, 90 min.

Chain-centered adducts were obtained by single electron transfer living radical polymerization (SET-LRP)^16^ of methyl acrylate (see Supporting Information (SI)), and their mechanical activation was performed in THF/H_2_O: 75/1 at 5–10°C, using high-intensity ultrasound, for 90 min (see Figure 1, Table 1, and SI section 5). ^1^H NMR analysis of the sonicated samples confirms that the *pushing* activation by the rotaxane actuator induces the release of *N*-triphenylmethyl maleimide **3**, via a formal retro-[4 + 2] cycloaddition as previously reported (Figure 2a).^9^ In contrast, the *pulling* activation of a similar Diels–Alder mechanophore (**4**), by the intermediacy of covalently linked polymer actuators, gives rise to two competing dissociation pathways: the anticipated r[4 + 2] process and a heterolytic scission of the C–N bond connecting the trityl (triphenylmethyl) group to the rest of the adduct. The r[4 + 2] pathway was confirmed by the emergence of the diagnostic furan (*c–e*, Figure 2b_iii_) and maleimide (*f*, Figure 2b_vi_) peaks in the postsonication mixture (Figure 2b_ii_). Similarly, the heterolytic pathway was revealed by the appearance of olefinic (*g–h*, Figure 2b_ii,iv_) and aromatic peaks (*i*, Figure 2b_ii,v_) of adduct **7** and triphenylmethanol derivative **8** respectively, which are likely coming from the reaction of the mechanically generated maleimide anion and trityl cation with water. The products that would suggest a homolytic scission of this C–N bond were not observed, and the heterolytic scission is supported by calculations (see SI). A similar reactivity was observed in an adduct lacking the terminal −CH_2_OAc group (Figure S7).

**Table 1:** Structural and Activation Parameters

			Mechanophore activation (%)^a^		
	*Presonic*. *M*_*n*_ *(kDa)/Đ*	*Postsonic*. *M*_*n*_ *(kDa)/Đ*	r[4 + 2]	*Heterol.*	*F*_*max*_*(nN)*^b^
**1**	118/1.14	44/1.28	58	0	4.1
**4**	112/1.25	47/1.26	27	20	4.4

a Determined from the integration of furan and adduct olefinic peaks (e.g., *b*, *e*, *h* in the postsonication ^1^H NMR spectrum of **4** (Figure 2b_ii_)). See SI section 6 for details.

b From CoGEF calculations. See SI section 7 for details.

**Figure 2:**
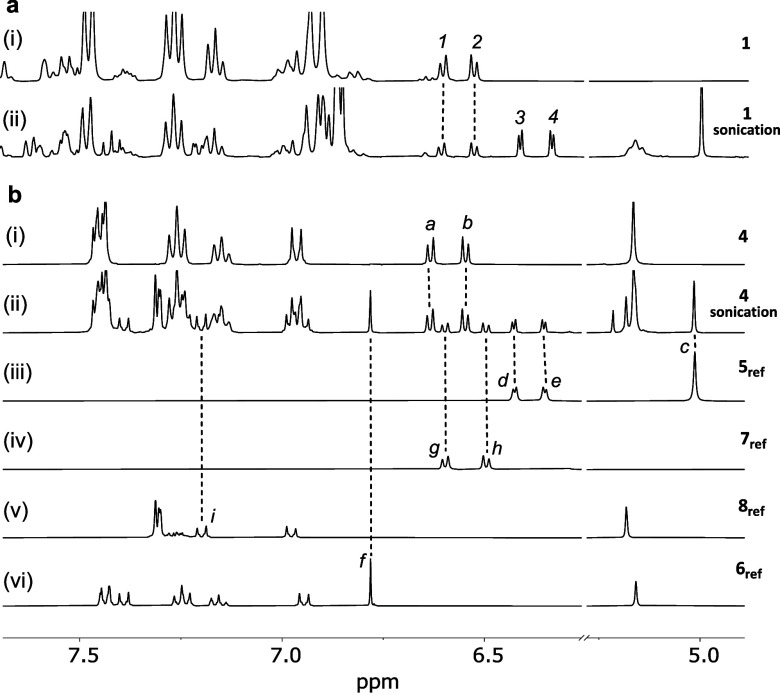
Partial ^1^H NMR (400 MHz, acetone-*d*_*6*_, 298 K) spectra of: (a) pushed mechanophore **1** before (i) and after (ii) sonication, and (b) pulled mechanophore **4** before (i) and after (ii) sonication, along with reference compounds **5**_**ref**_ (iii) and **7**_**ref**_ (iv), **8**_**ref**_ (v), and **6**_**ref**_ (vi), which are independently synthesized references of compounds **5**–**8** (see SI). The assignments correspond to the lettering shown in Figure 1.

The simulated elongation of models of the interlocked (rotaxane) and noninterlocked mechanophores (Figure 3a) shed light on the difference in reactivity between the two modes of actuation investigated (Figure 3). CoGEF (Constrained Geometries Simulate External Force) calculations^17^ (DFT B3LYP/6-31G*, gas) predict a retro-[4 + 2] cycloaddition pathway for both mechanophores (see Figure 3a and SI). Despite similar predicted outcomes, the *pushing* and *pulling* actuations differ greatly in the way they activate the mechanophore. We compared three parameters indicative of this phenomenon: the elongation of putative C–C and C–N scissile bonds *a* and *b* (Figure 3a,b), their related bond angles α and β (Figure 3a,c), and dihedral angle ω (Figure 3a,d). A substantial increase in the length of bond *a*, accompanied by an opening of angle α, is observed upon elongation of the rotaxane model. This is expected from the action of macrocycle *pushing* against the mechanophore. However, this actuation leaves C–N bond *b*, and related angle β, almost unaffected, indicating that the *pushing* actuation does not induce any deformation in the upper part of the mechanophore. Instead, the bottom part experiences a twisting of the maleimide ring, expressed by the opening of dihedral angle ω. This twisting is not observed in the *pulling* actuation, but in this case both bonds *a* and *b*, and both angles α and β increase substantially during elongation. Interestingly, bond *a* is elongating faster than *b*, which explains the predicted r[4 + 2] pathway and its preponderance during sonication (Table 1). Nevertheless, the high deformation of bond *b* and angle β gives access to the heterolytic pathway as well, potentially under the impulse of dynamic effects.^18−21^

**Figure 3:**
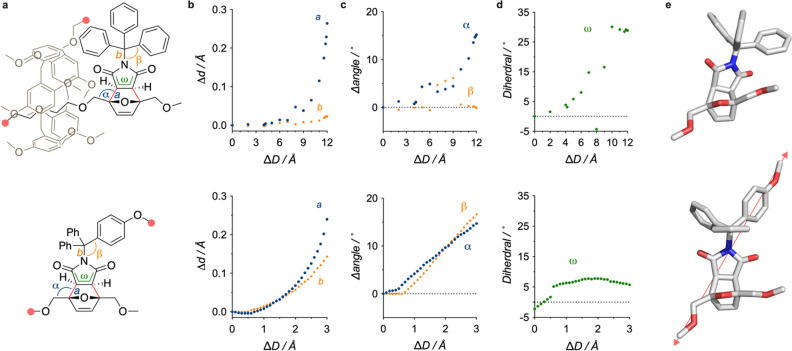
Computational investigation of the *pushing* and *pulling* actuations (CoGEF, DFT entry B3LYP/6-31G*, gas). (a) Models in the computation indicating key structural parameters. Predicted scissile bond are shown in red. Anchor atoms are indicated by the pink disks. Evolution of bond *a* and *b* (b), angles α and β (c), and dihedral ω (d) upon simulated elongation of rotaxane (top) and linear (bottom) models. (e) Equilibrium geometries at the *E*_*max*_ of the Diels–Alder mechanophore upon pushing (top) or pulling (bottom). Macrocycle and axle omitted for clarity in the top structure. Elongation vector shown as a pink arrow in the bottom structure.

The effect of these actuations can be visualized (Figure 3e) in the structure of the mechanophores at maximal deformation (*E*_*max*_, i.e., just before cleavage). In the rotaxane, the maleimide is *pushed* away from the macrocycle, and most of the deformation develops at the contact between the macrocycle and the mechanophore (Figure 3e). This embrace is even more striking in the space-filling model of the rotaxane (Figure 4), which shows the extent of the contact surface between the mechanophore and the incoming macrocycle. In contrast, the pulled mechanophore experiences a more acute deformation that propagates along the vector connecting the two pulling points (Figure 3e). This point-to-point deformation leads to the activation of the most compliant bonds along the way.

**Figure 4:**
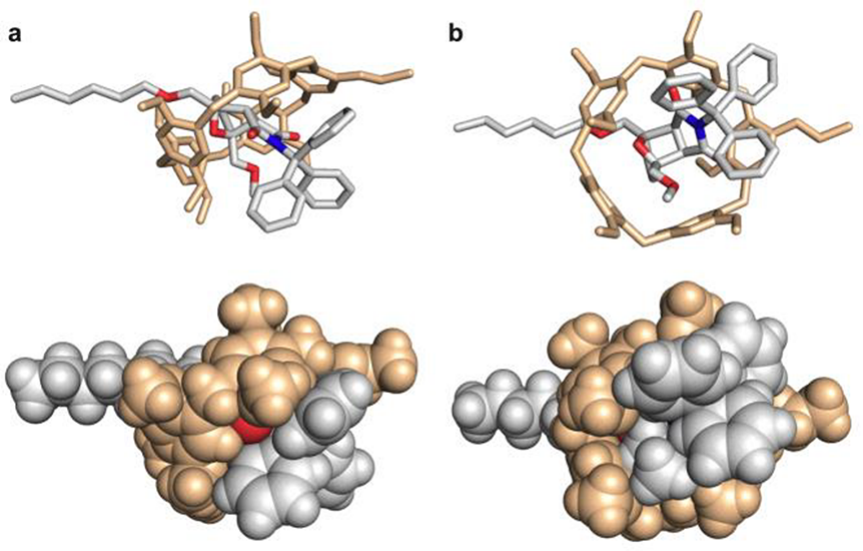
Space-filling model of the rotaxane actuator at maximal deformation (*E*_*max*_ from CoGEF, see SI) reveals the extent of the contact surface between the mechanophore and the macrocycle. Side (a) and bottom (b) view of the rotaxane at *E*_*max*_ in tube (top) and space-filling (bottom) representation. Hydrogen atoms are omitted for clarity in the tube representation.

In conclusion, we have compared the unique *pushing* geometry of activation provided by a rotaxane actuator, with the more common *pulling* activation. We found that *pushing* led to a more efficient and selective activation of a bulky Diels–Alder mechanophore than *pulling*. In the latter case, two competing pathways were observed as the mechanophore followed either the desired retro-[4 + 2] cycloaddition or underwent the heterolytic cleavage of a C–N bond to produce a trityl cation. This difference in behavior is explained by the rotaxane’s ability to provide a more diffuse activation due to the large contact area between the macrocycle and the mechanophore, while *pulling* provokes acute deformation (i.e., bond bending and stretching) in the molecular backbone connecting the two *pulling* points.

## Supporting Information Available

Detailed descriptions of CoGEF calculations, synthetic procedures, characterization of new compounds, and spectroscopic data (PDF)
