# Supplementary material for: Pushing vs Pulling: The Unique Geometry of Mechanophore Activation in a Rotaxane Force Actuator
Source: J Am Chem Soc. 2024 Jun 7;146(24):16381–4. doi: 10.1021/jacs.4c05168 (PMC11191692; doi:10.1021/jacs.4c05168)
Supplement: Supplementary file 1 — ja4c05168_si_001.pdf [file ja4c05168_si_001.pdf]

Supporting Information

Pushing vs Pulling: the Unique Geometry of Mechanophore Activation in a Rotaxane Force Actuator

*Lei Chen and Guillaume De Bo**

Department of Chemistry, University of Manchester, Oxford Road, Manchester, M13 9PL, UK

*E-mail: guillaume.debo@manchester.ac.uk

1 Table of Contents

1 Table of Contents **2**
2 General Experimental Details **4**
3 Synthesis of Mechanophores and Reference Compounds **5**
3.1 Synthesis of Mechanophores 5
3.1.1 Synthetic Routes to **S5** and **S7** 5
3.1.2 Synthesis of **S4** 5
3.1.3 Synthesis of **S5** 6
3.1.4 Synthesis of **S6** 7
3.1.5 Synthesis of **S7** 7
3.2 Synthesis of Reference Compounds 8
3.2.1 Synthetic Routes to **S8**, **S9**, **S11**, **S13** and **S15** 8
3.2.2 Synthesis of **S8** 9
3.2.3 Synthesis of **S9** 9
3.2.4 Synthesis of **S11** 10
3.2.5 Synthesis of **S13** 10
3.2.6 Synthesis of **S14** 11
3.2.7 Synthesis of **S15** 11
4 Synthesis of Polymers **12**
4.1 Representative Procedure for SET-LRP of Methyl Acrylate Using Mechanophore Initiators 12
4.2 Synthesis of Polymer with Mechanophores 12
4.2.1 Synthesis of Polymer **1** 12
4.2.2 Synthesis of Polymer **4** 13
4.2.3 Synthesis of Polymer **S17** 13
4.3 Synthesis of Reference Polymers 13
4.3.1 Synthesis of Polymer **5** 13
4.3.2 Synthetic Route to Polymer **6** and **7** 14
4.3.3 Synthesis of Polymer **S18** 14
4.3.4 Synthesis of Polymer **6** 14
4.3.5 Synthesis of Polymer **7** 15
4.3.6 Synthesis of Polymer **8** 15
4.3.7 Synthesis of Polymer **S19** 15
4.3.8 Synthesis of Polymer **S20** 15
4.3.9 Synthesis of Polymer **S21** 16
4.4 SEC Data for Synthesised Polymers 16
4.5 SEC Traces for Polymers with Mechanophore 17
4.6 SEC Traces for Reference Polymers 18
5 Mechanophore Activation via Ultrasound **19**
5.1 General Procedure for Sonication Experiments 19
5.2 Sonication of Polymer **1** 19
5.3 Sonication of Polymer **4** 22
5.4 Sonication of Polymer **S17** 25
5.5 Kinetic Investigation Overview 27
6 Calculation of Extent of Mechanophore Activation **30**
6.1 Calculations for Polymer **1** 30
6.2 Calculations for Polymers **4** and **S17** 31
6.3 Summary of Mechanophores Activated by Sonication 33
7 CoGEF Calculations **34**
7.1 General method 34
7.2 CoGEF of linear models (pulling) 34
7.3 CoGEF of rotaxane models (pulling) 34
7.4 Heterolytic cleavage of C-N bond in a distal-exo model 35

8 NMR Spectra ... **37**
8.1 Small Molecule NMR Spectra ... 37
8.1.1 Spectra of **S4** ... 37
8.1.2 Spectra of **S5** ... 38
8.1.3 Spectra of **S6** ... 39
8.1.4 Spectra of **S7** ... 40
8.1.5 Spectra of **S8** ... 41
8.1.6 Spectra of **S9** ... 42
8.1.7 Spectra of **S11** ... 43
8.1.8 Spectra of **S13** ... 44
8.1.9 Spectra of **S14** ... 45
8.1.10 Spectra of **S15** ... 46
8.2 Polymer NMR Spectra ... 47
8.2.1 Spectra of polymer **1** ... 47
8.2.2 Spectra of polymer **4** ... 47
8.2.3 Spectra of polymer **S17** ... 48
8.2.4 Spectra of polymer **5** ... 48
8.2.5 Spectra of polymer **S18** ... 49
8.2.6 Spectra of polymer **6** ... 49
8.2.7 Spectra of polymer **7** ... 50
8.2.8 Spectra of polymer **8** ... 50
8.2.9 Spectra of polymer **S19** ... 51
8.2.10 Spectra of polymer **S20** ... 51
8.2.11 Spectra of polymer **S21** ... 52
8.3 Post-Sonication NMR Spectra ... 52
8.3.1 Post-Sonication ^{1}H NMR Spectra of Polymer **1** ... 52
8.3.2 Post-Sonication ^{1}H NMR Spectra of Polymer **4** ... 54
8.3.1 Post-Sonication ^{1}H NMR Spectra of Polymer **S17** ... 55
9 Mass Spectrometry Isotopic Patterns ... **57**
9.1 Isotopic distribution of **S5** ... 57
9.2 Isotopic distribution of **S7** ... 57
10 References ... **58**

2 General Experimental Details

Unless otherwise stated, all reagents and solvents were purchased from commercial suppliers and used without further purification. Dry solvents were obtained by passing through an activated alumina column on a Phoenix SDS solvent drying system (JC Meyer Solvent Systems, CA, USA). Compound **S2**[1], **S1**, **S3**, **S12**, **S16**, **S23**, **3**[2] and **S10**[3] were prepared according to literature procedure.

Size exclusion chromatography (SEC) analyses were performed in THF solution (1.0 mg mL^{-1}) at 40 °C using a GPC/SEC Agilent 1260 Infinity II with 2 × PL gel 10 µm mixed-B (or mixed-C where indicated) and a PL gel 500 Å column, and equipped with a differential refractive index (DRI) detector employing narrow polydispersity polystyrene standards (Agilent Technologies) as a calibration reference. Samples were filtered through a Whatman Puradisc 4 mm syringe filter with 0.45 µm PTFE membrane before injection to equipment, and experiments were carried out with injection volume of 50 µL, flow rate of 1 mL min^{-1}. Results were analyzed using *n*-dodecane as internal marker using Agilent GPC/SEC Software Version 2.2.

Ultrasound experiments were performed using a Sonics VCX 500 ultrasonic processor equipped with a 13 mm diameter solid or replaceable-tip probe. The distance between the titanium tip and the bottom of the Suslick cell was 2 cm. The ultrasonic intensity was calibrated using the method outlined by Hickenboth *et al*.[4] The Suslick cells were fabricated by the Department of Chemistry glass workshop at the University of Manchester.

Analytical TLC was performed on precoated silica gel plates (0.25 mm thick, 60 F254, Merck, Germany) and observed under UV light or stained with a potassium permanganate base solution. Preparative TLC was performed on precoated silica gel plates: 500 µm or 2000 µm, UNIPLATE GF, Analtech Inc., DE, USA. Flash column chromatography was performed with silica gel 60 (230-400 mesh) from Sigma-Aldrich. ^{1}H and ^{13}C NMR spectra were recorded on a Bruker Avance III 600 MHz Prodigy instrument, a Bruker Avance III 500 MHz Prodigy instrument or a Bruker Avance III 400 MHz Prodigy instrument. Chemical shifts are reported in parts per million (ppm) from high to low frequency and referenced to the residual solvent resonance. Coupling constants (*J*) are reported in Hertz (Hz) and splitting patterns are designated as follows: b = broad, s = singlet, d = doublet, t = triplet, q = quartet, p = pentet and m = multiplet. ^{1}H and ^{13}C assignments were made using 1D or 2D NMR methods (HSQC, HMBC, COSY). Mass spectra were obtained through the Mass Spectrometry services in the Department of Chemistry at the University of Manchester.

Abbreviations: BiBB: bromoisobutyryl bromide; CoGEF: constrained geometries simulate external force; DCM: dichloromethane; DMSO: dimethylsulfoxide; ESI: electrospray ionization; EtOAc: ethyl acetate; Et_2O: diethyl ether; HRMS: high resolution mass spectrometry; MA: methyl acrylate; MeCN: acetonitrile; MeOH: methanol; MS: mass spectrometry; Me_6TREN: tris[2-(dimethylamino)ethyl]amine; PE: petroleum ether; PMDETA: N,N,N′,N′′,N′′-Pentamethyldiethylenetriamine; THF: tetrahydrofuran; TLC: thin layer chromatography.

3 Synthesis of Mechanophores and Reference Compounds

3.1 Synthesis of Mechanophores

3.1.1 Synthetic Routes to S5 and S7

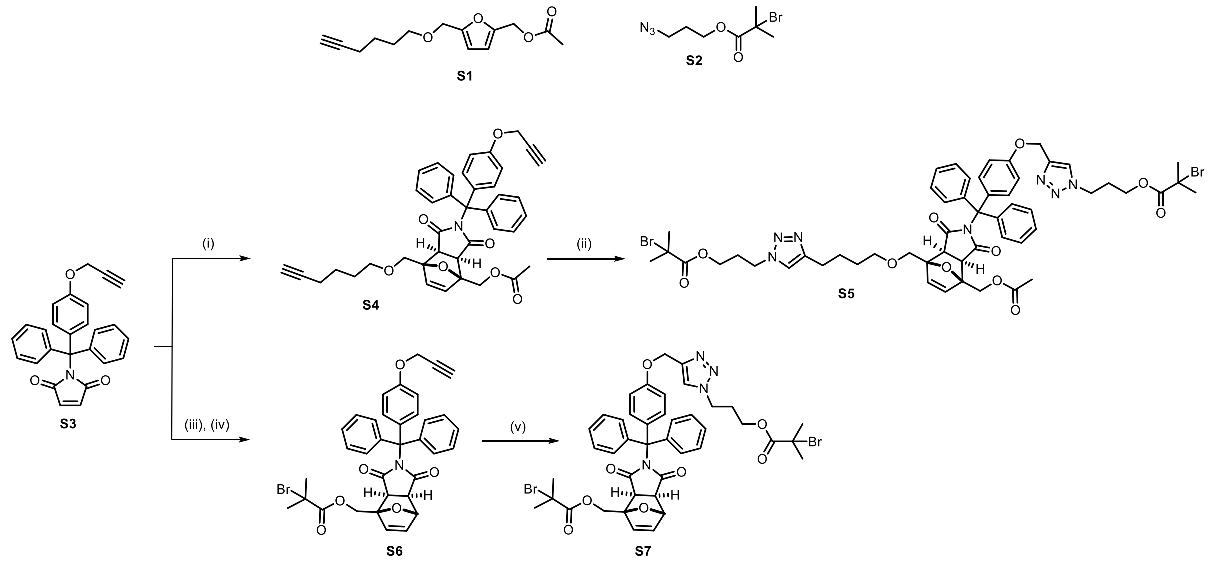

Scheme S1. Synthetic routes to **S5** and **S7**. Conditions: (i) **S1**, MeCN, 70 °C, 16 h, 31% yield; (ii) **S2**, CuBr, PMDETA, DCM, r.t., 2 h, 68% yield; (iii) Furfuryl alcohol, MeCN, 70 °C, 16 h; (iv) BiBB, Et_3N, DCM, r.t., 16 h, 23% yield; (v) **S2**, CuBr, PMDETA, DCM, r.t., 2 h, 57% yield.

3.1.2 Synthesis of S4

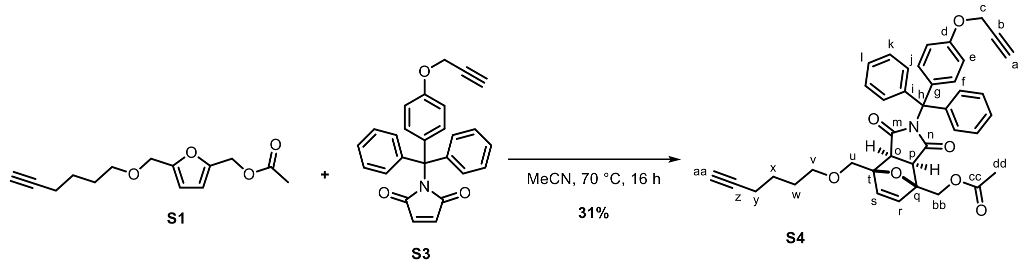

A solution of **S1** (50 mg, 200 µmol, 1.0 eq.) and **S3** (314 mg, 800 µmol, 4.0 eq.) in acetonitrile (1 mL) was stirred for 16 h at 70 °C. After the solvent was evaporated, the residue was purified via column chromatography (SiO_2, DCM, 100% to DCM/MeOH, 20:1) to give a crude of *endo* and *exo* isomers. Then, this crude was further purified by preparative TLC (2000 µm, PE/EtOAc, 5/2, eluted twice) to yield **S4** as a white powder (40 mg, 62 µmol, 31% yield).

^{1}H NMR (400 MHz, Acetone-d_6, 298 K) δ = 7.49 – 7.41 (m, 6H, $H_{f,j}$), 7.28 – 7.21 (m, 4H, H_k), 7.17 – 7.10 (m, 2H, H_l), 6.94 – 6.88 (m, 2H, H_e), 6.62 (d, *J* = 5.6 Hz, 1H, H_r), 6.54 (d, *J* = 5.6 Hz, 1H, H_s), 4.76 (d, *J* = 2.4 Hz, 2H, H_c), 4.73 (s, 2H, H_{bb}), 4.18 (d, *J* = 11.7 Hz, 1H, H_u), 3.91 (d, *J* = 11.7 Hz, 1H, H_u), 3.72 – 3.64 (m, 1H, H_v), 3.61 – 3.53 (m, 1H, H_v), 3.07 (t, *J* = 2.4 Hz, 1H, H_a), 2.98 (d, *J* = 6.5 Hz, 1H, H_o or H_p), 2.94 (d, *J* = 6.5 Hz, 1H, H_p or H_o), 2.33 (t, *J* = 2.7 Hz, 1H, H_{aa}), 2.23 (td, *J* = 7.0, 2.7 Hz, 2H, H_y), 2.08 (s, 3H, H_{dd}), 1.76 – 1.68 (m, 2H, H_w), 1.67 – 1.57 (m, 2H, H_x).
^{13}C NMR (101 MHz, Acetone-d_6, 298 K) δ = 173.91, 173.88 ($C_{m,n}$), 170.71 (C_{cc}), 157.28 (C_d), 143.87,

143.81 (C_i), 139.44 (C_r), 137.93 (C_s), 135.71 (C_g), 131.16 (C_f), 128.99, 128.96 (C_j), 128.29, 128.27 (C_k), 127.05 (C_l), 114.32 (C_e), 92.33 (C_t), 90.75 (C_q), 84.93 (C_z), 79.78 (C_b), 77.01 (C_a), 73.89 (C_h), 71.82 (C_v), 69.99 (C_{aa}), 69.02 (C_u), 62.24 (C_{bb}), 56.20 (C_c), 51.39, 51.36 ($C_{o,p}$), 29.84 (C_w, overlapped with solvent peak), 26.06 (C_x), 20.71 (C_{dd}), 18.51 (C_y).
HRMS-ESI(+): 666.2465 $[M+Na]^+$, calculated for $C_{40}H_{37}NO_7Na^+$: 666.2462.

3.1.3 Synthesis of S5

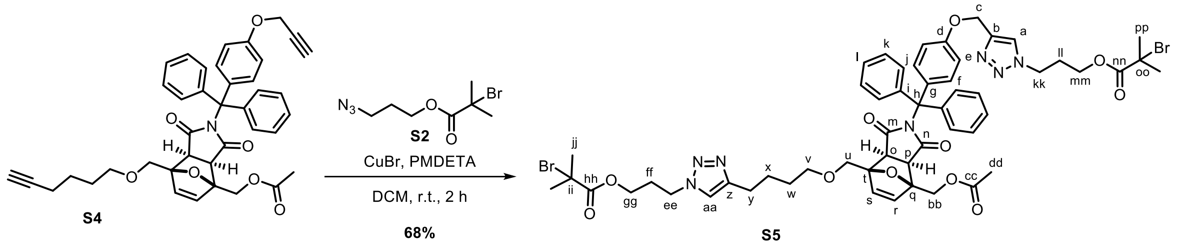

Three sealed 5 mL microwave vials (*A*, *B*, and *C*) were prepared as follows: *A* contained PMDETA (3 mg, 16 µmol, 1.0 eq.) in DCM (3 mL), *B* contained CuBr (2 mg, 16 µmol, 1.0 eq.), and *C* contained **S4** (10 mg, 16 µmol, 1.0 eq.) and **S2** (8 mg, 33 µmol, 2.1 eq.). Vials *B* and *C* were subjected to three N_2/vacuum cycles before use. The solution in vial *A* was degassed by bubbling with N_2 until ~1 mL DCM left before being transferred to vial *B via* cannula; the resulting mixture was stirred until all CuBr had dissolved. The CuBr/PMDETA solution in vial *B* was then transferred to vial *C via* cannula; the resulting reaction mixture was stirred for 2 h at room temperature. The reaction mixture was washed by aqueous EDTA solution (0.25 M, pH 7, 2 x 1 mL) and brine (2 x 1 mL). The organic layer was collected and dried with magnesium sulfate. The mixture was filtered before being concentrated under vacuum. The residue was purified by preparative TLC (500 µm, DCM/MeOH, 30/1) to yield **S5** as a white powder (12 mg, 11 µmol, 68% yield).

^{1}H NMR (400 MHz, Acetone-d_6, 298 K) δ = 8.09 (s, 1H, H_a), 7.70 (s, 1H, H_{aa}), 7.48 – 7.40 (m, 6H, $H_{f,j}$), 7.29 – 7.21 (m, 4H, H_k), 7.17 – 7.10 (m, 2H, H_l), 6.98 – 6.91 (m, 2H, H_e), 6.62 (d, J = 5.6 Hz, 1H, H_r), 6.53 (d, J = 5.6 Hz, 1H, H_s), 5.15 (s, 2H, H_c), 4.73 (s, 2H, H_{bb}), 4.59 (t, J = 6.9 Hz, 2H, H_{kk}), 4.50 (t, J = 6.9 Hz, 2H, H_{ee}), 4.26 – 4.13 (m, 5H, $H_{mm,gg,u}$), 3.91 (d, J = 11.6 Hz, 1H, H_u), 3.72 – 3.65 (m, 1H, H_v), 3.61 – 3.53 (m, 1H, H_v), 2.98 (d, J = 6.5 Hz, 1H, H_o or H_p), 2.94 (d, J = 6.5 Hz, 1H, H_p or H_o), 2.71 (t, J = 7.4 Hz, 2H, H_y), 2.39 – 2.32 (m, 2H, H_{ll}), 2.32 – 2.25 (m, 2H, H_{ff}), 2.06 (s, 3H, H_{dd}), 1.96 – 1.91 (s, s, 12H, $H_{jj,pp}$), 1.82 – 1.72 (m, 2H, H_x), 1.71 – 1.61 (m, 2H, H_w).
^{13}C NMR (101 MHz, Acetone-d_6, 298 K) δ = 173.92, 173.90 ($C_{m,n}$), 171.75 ($C_{hh,nn}$), 170.75 (C_{cc}), 158.11 (C_d), 148.28 (C_z), 144.33 (C_b), 143.88, 143.85 (C_i), 139.51 (C_r), 137.92 (C_s), 135.36 (C_g), 131.14 (C_f), 129.03, 129.01 (C_j), 128.28, 128.26 (C_k), 127.05 (C_l), 124.82 (C_a), 122.24 (C_{aa}), 114.31 (C_e), 92.31 (C_t), 90.73 (C_q), 73.93 (C_h), 72.15 (C_v), 69.00 (C_u), 63.53 ($C_{mm,gg}$), 62.38 (C_c), 62.27 (C_{bb}), 57.41, 57.40 ($C_{oo,ii}$), 51.38, 51.37 ($C_{o,p}$), 47.55 (C_{kk}), 47.17 (C_{ee}), 30.01 – 29.89 ($C_{w,ll,ff}$, overlapped with solvent peak), 26.86 (C_x), 25.99 (C_y), 20.72 (C_{dd}).
HRMS-ESI(+): 1164.2723 $[M+Na]^+$, calculated for $C_{54}H_{61}Br_2N_7O_{11}Na^+$: 1164.2688.

3.1.4 Synthesis of S6

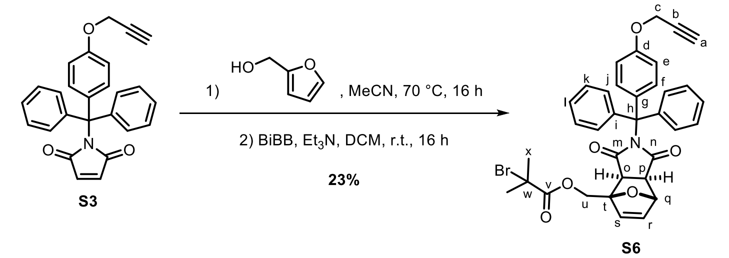

To a solution of **S3** (40 mg, 102 µmol, 1.0 eq.) in acetonitrile (1 mL) was added furfuryl alcohol (100 mg, 1018 µmol, 10.0 eq.). The mixture was then stirred for 16 h at 70 °C. After the solvent was evaporated, the residue was purified via column chromatography (SiO_2, PE/Et_2O, 2/1) to give a mixture of *endo* and *exo* isomers. Then, this mixture was dissolved in DCM (1 mL) before BiBB (19 µL, 153 µmol, 1.5 eq.) and Et_3N (21 µL, 153 µmol, 1.5 eq.) were added in sequence and stirred for 16 h at room temperature. The reaction mixture was concentrated under vacuum, and the residue was purified by preparative TLC (2000 µm, PE/EtOAc, 2/1, eluted twice) to yield **S6** as a white powder (15 mg, 23 µmol, 23% yield).

^{1}H NMR (400 MHz, Acetone-d_6, 298 K) δ = 7.48 – 7.39 (m, 6H, $H_{f,j}$), 7.28 – 7.20 (m, 4H, H_k), 7.17 – 7.10 (m, 2H, H_l), 6.93 – 6.87 (m, 2H, H_e), 6.71 – 6.66 (m, 1H, H_r), 6.57 (d, J = 5.7 Hz, 1H, H_s), 5.31 (d, J = 1.8 Hz, 1H, H_q), 4.86 (d, J = 12.8 Hz, 1H, H_u), 4.80 – 4.70 (m, 3H, $H_{c,u}$), 3.09 (t, J = 2.4 Hz, 1H, H_a), 3.00 – 2.94 (m, 2H, $H_{o,p}$), 2.01 – 1.91 (s, s, 6H, H_x).
^{13}C NMR (101 MHz, Acetone-d_6, 298 K) δ = 175.18 (C_n), 174.17 (C_m), 171.48 (C_v), 157.17 (C_d), 143.86 (C_i), 138.39 (C_r), 137.45 (C_s), 135.77 (C_g), 131.09 (C_f), 128.96, 128.90 (C_j), 128.26, 128.25 (C_k), 126.99, 126.93 (C_l), 114.26 (C_e), 90.60 (C_t), 82.44 (C_q), 79.77 (C_b), 77.02 (C_a), 73.79 (C_h), 63.66 (C_u), 56.97 (C_w), 56.12 (C_c), 50.39 (C_p), 49.07 (C_o).
HRMS-ESI(+): 662.1138 $[M+Na]^+$, calculated for $C_{35}H_{30}BrNO_6Na^+$: 662.1149.

3.1.5 Synthesis of S7

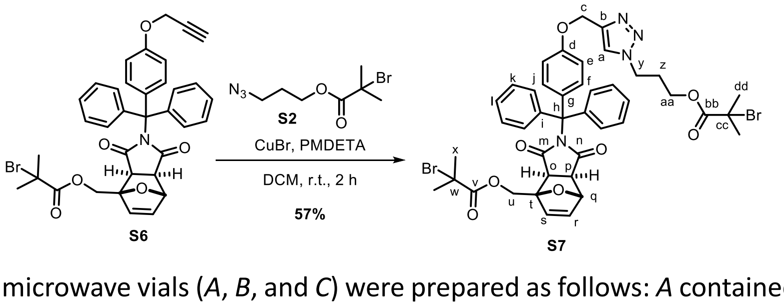

Three sealed 5 mL microwave vials (*A*, *B*, and *C*) were prepared as follows: *A* contained PMDETA (4 mg, 23 µmol, 1.0 eq.) in DCM (3 mL), *B* contained CuBr (3 mg, 23 µmol, 1.0 eq.), and *C* contained **S6** (15 mg, 23 µmol, 1.0 eq.) and **S2** (6 mg, 23 µmol, 1.0 eq.). Vials *B* and *C* were subjected to three N_2/vacuum cycles before use. The solution in vial *A* was degassed by bubbling with N_2 until ~1 mL DCM left before being transferred to vial *B via* cannula; the resulting mixture was stirred until all CuBr had dissolved. The CuBr/PMDETA solution in vial *B* was then transferred to vial *C via* cannula; the resulting reaction mixture was stirred for 2 h at room temperature. The reaction mixture was washed by aqueous EDTA solution (0.25 M, pH 7, 2 x 1 mL) and brine (2 x 1 mL). The organic layer was collected and dried with magnesium sulfate. The mixture was filtered before being concentrated under vacuum. The residue was purified by preparative TLC (500 µm, PE/acetone, 2/1) to yield **S7** as a white powder (12 mg, 13

μmol, 57% yield).

^{1}H NMR (400 MHz, Acetone-d_6, 298 K) δ = 8.09 (s, 1H, H_a), 7.47 – 7.37 (m, 6H, $H_{f,j}$), 7.28 – 7.19 (m, 4H, H_k), 7.17 – 7.08 (m, 2H, H_l), 6.98 – 6.90 (m, 2H, H_e), 6.71 – 6.64 (m, 1H, H_r), 6.57 (d, J = 5.7 Hz, 1H, H_s), 5.30 (d, J = 1.8 Hz, 1H, H_q), 5.14 (s, 2H, H_c), 4.86 (d, J = 12.8 Hz, 1H, H_u), 4.74 (d, J = 12.7 Hz, 1H, H_u), 4.59 (t, J = 6.9 Hz, 2H, H_y), 4.22 (t, J = 6.1 Hz, 2H, H_{aa}), 3.01 – 2.94 (m, 2H, $H_{o,p}$), 2.34 (p, J = 6.5 Hz, 2H, H_z), 1.96 – 1.93 (s, s, 6H, H_x), 1.93 (s, 6H, H_{dd}).
^{13}C NMR (101 MHz, Acetone-d_6, 298 K) δ = 175.19 (C_n), 174.18 (C_m), 171.70 (C_{bb}), 171.49 (C_v), 158.02 (C_d), 144.28 (C_b), 143.93, 143.88 (C_i), 138.39 (C_r), 137.46 (C_s), 135.38 (C_g), 131.11 (C_f), 128.94 (C_j), 128.25, 128.23 (C_k), 126.94, 126.92 (C_l), 124.82 (C_a), 114.24 (C_e), 90.61 (C_t), 82.45 (C_q), 73.82 (C_h), 63.68 (C_u), 63.48 (C_{aa}), 62.28 (C_c), 57.38 (C_{cc}), 56.98 (C_w), 50.38 (C_p), 49.07 (C_o), 47.50 (C_y), 30.96 (C_x), 30.84 (C_{dd}), 29.93 – 29.76 (C_z, overlapped with solvent peak).
HRMS-ESI(+): 911.1251 [M+Na]$^+$, calculated for $C_{42}H_{42}Br_2N_4O_8Na^+$: 911.1262.

3.2 Synthesis of Reference Compounds

3.2.1 Synthetic Routes to S8, S9, S11, S13 and S15

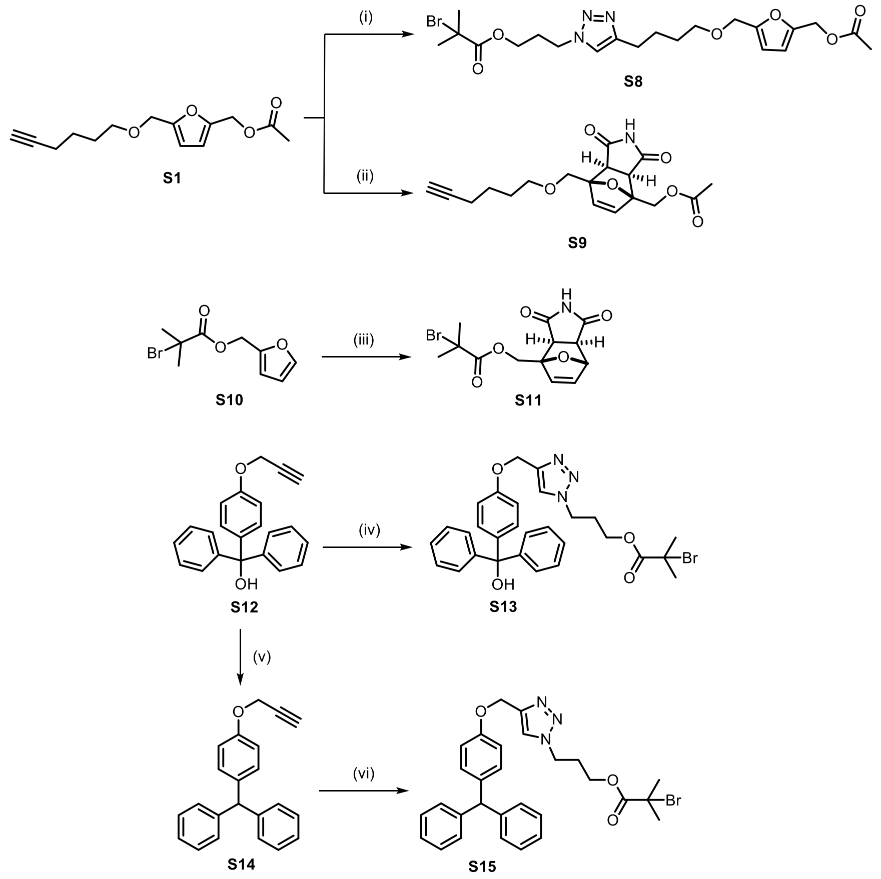

Scheme S2. Synthetic routes to **S8**, **S9**, **S11**, **S13** and **S15**. Conditions: (i) **S2**, CuBr, PMDETA, DCM, r.t., 2 h, 60% yield; (ii) Maleimide, Toluene, 90 °C, 16 h, 48% yield; (iii) Maleimide, MeCN, 70 °C, 16 h, 29% yield; (iv) **S2**, CuBr, PMDETA, DCM, r.t., 2 h, 67% yield; (v) Et_3SiH, TFA, DCM, r.t., 1h, 84% yield; (vi) **S2**, CuBr, PMDETA, DCM, r.t., 2 h, 87% yield.

3.2.2 Synthesis of S8

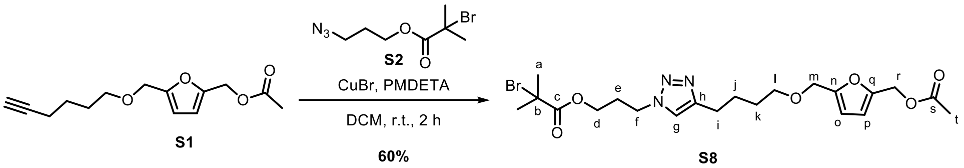

Three sealed 5 mL microwave vials (*A*, *B*, and *C*) were prepared as follows: *A* contained PMDETA (7 mg, 40 µmol, 1.0 eq.) in DCM (3 mL), *B* contained CuBr (6 mg, 40 µmol, 1.0 eq.), and *C* contained **S1** (10 mg, 40 µmol, 1.0 eq.) and **S2** (10 mg, 40 µmol, 1.0 eq.). Vials *B* and *C* were subjected to three N_2/vacuum cycles before use. The solution in vial *A* was degassed by bubbling with N_2 until ~1 mL DCM left before being transferred to vial *B via* cannula; the resulting mixture was stirred until all CuBr had dissolved. The CuBr/PMDETA solution in vial *B* was then transferred to vial *C via* cannula; the resulting reaction mixture was stirred for 2 h at room temperature. The reaction mixture was washed by aqueous EDTA solution (0.25 M, pH 7, 2 x 1 mL) and brine (2 x 1 mL). The organic layer was collected and dried with magnesium sulfate. The mixture was filtered before being concentrated under vacuum. The residue was purified by preparative TLC (500 µm, PE/acetone, 3/1, eluted twice) to yield **S8** as a white powder (12 mg, 24 µmol, 60% yield).

^{1}H NMR (400 MHz, Acetone-*d*$_6$, 298 K) δ = 7.70 (s, 1H, H_g), 6.42 (d, J = 3.1 Hz, 1H, H_p), 6.34 (d, J = 3.1 Hz, 1H, H_o), 5.01 (s, 2H, H_r), 4.51 (t, J = 6.9 Hz, 2H, H_f), 4.40 (s, 2H, H_m), 4.20 (t, J = 6.1 Hz, 2H, H_d), 3.49 (t, J = 6.3 Hz, 2H, H_l), 2.66 (t, J = 7.4 Hz, 2H, H_i), 2.30 (p, J = 6.6 Hz, 2H, H_e), 2.01 (s, 3H, H_t), 1.93 (s, 6H, H_a), 1.75 – 1.66 (m, 2H, H_j), 1.65 – 1.56 (m, 2H, H_k).
^{13}C NMR (101 MHz, Acetone-*d*$_6$, 298 K) δ = 171.74 (C_c), 170.61 (C_s), 154.15 (C_n), 150.76 (C_q), 148.22 (C_h), 122.17 (C_g), 111.98 (C_p), 110.62 (C_o), 70.42 (C_l), 65.17 (C_m), 63.51 (C_d), 58.47 (C_r), 57.40 (C_b), 47.16 (C_f), 30.90 (C_a), 29.99 – 29.87 ($C_{e,k}$, overlapped with solvent peak), 26.90 (C_j), 25.97 (C_i), 20.68 (C_t).
HRMS-ESI(+): 522.1194 $[M+Na]^+$, calculated for $C_{21}H_{30}BrN_3O_6Na^+$: 522.1210.

3.2.3 Synthesis of S9

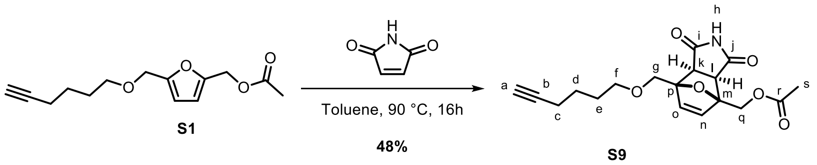

A solution of **S1** (30 mg, 120 µmol, 1.0 eq.) and maleimide (233 mg, 2399 µmol, 20.0 eq.) in toluene (1 mL) was stirred for 16 h at 90 °C. After the solvent was evaporated, the residue was purified via column chromatography (SiO_2, DCM, 100% to DCM/MeOH, 20:1) to give a crude of *endo* and *exo* isomers. Then, this crude was further purified by preparative TLC (2000 µm, PE/Et_2O, 1/2, eluted twice) to yield **S9** as a white powder (20 mg, 58 µmol, 48% yield).

^{1}H NMR (500 MHz, Acetone-*d*$_6$, 298 K) δ = 10.12 (br, 1H, H_h), 6.59 (d, J = 5.6 Hz, 1H, H_n), 6.49 (d, J = 5.6 Hz, 1H, H_o), 4.79 (d, J = 12.8 Hz, 1H, H_q), 4.43 (d, J = 12.8 Hz, 1H, H_q), 4.15 (d, J = 11.6 Hz, 1H, H_g), 3.77 (d, J = 11.5 Hz, 1H, H_g), 3.64 – 3.57 (m, 1H, H_f), 3.56 – 3.50 (m, 1H, H_f), 3.15 (d, J = 6.5 Hz, 1H, H_k or H_l), 3.12 (d, J = 6.5 Hz, 1H, H_l or H_k), 2.31 (t, J = 2.6 Hz, 1H, H_a), 2.22 (td, J = 7.0, 2.6 Hz, 2H, H_c), 2.03 (s, 3H, H_s), 1.72 – 1.64 (m, 2H, H_e), 1.63 – 1.55 (m, 2H, H_d).
^{13}C NMR (126 MHz, Acetone-*d*$_6$, 298 K) δ = 175.82, 175.73 ($C_{i,j}$), 170.72 (C_r), 139.44 (C_n), 137.82 (C_o), 91.64 (C_p), 90.01 (C_m), 84.93 (C_b), 71.58 (C_f), 69.92 (C_a), 68.85 (C_g), 62.44 (C_q), 52.98, 52.95 ($C_{k,l}$), 29.38

(C_e), 26.04 (C_d), 20.62 (C_s), 18.45 (C_c).
HRMS-ESI(+): 370.1248 $[M+Na]^+$, calculated for $C_{18}H_{21}NO_6Na^+$: 370.1261.

3.2.4 Synthesis of S11

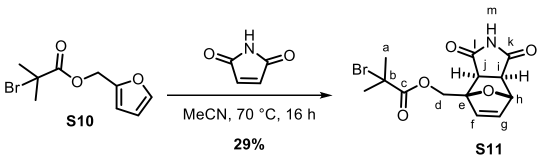

To a solution of **S10** (100 mg, 407 µmol, 1.0 eq.) in acetonitrile (0.5 mL) was added maleimide (39 mg, 407 µmol, 1.0 eq.). The mixture was then stirred for 16 h at 70 °C. After the solvent was evaporated, the residue was purified by preparative TLC (2000 µm, PE/Et_2O, 1/2, eluted twice) to yield **S11** as a white powder (40 mg, 117 µmol, 29% yield).

^{1}H NMR (400 MHz, Acetone-d_6, 298 K) δ = 10.03 (br, 1H, H_m), 6.65 (ddd, J = 5.7, 1.8, 0.8 Hz, 1H, H_g), 6.54 (d, J = 5.7 Hz, 1H, H_f), 5.18 (d, J = 1.8 Hz, 1H, H_h), 4.92 (d, J = 12.6 Hz, 1H, H_d), 4.57 (dd, J = 12.7, 0.9 Hz, 1H, H_d), 3.11 (d, J = 6.5 Hz, 1H, H_i), 3.07 (d, J = 6.5 Hz, 1H, H_j), 1.94 – 1.90 (s, s, 6H, H_a).
^{13}C NMR (101 MHz, Acetone-d_6, 298 K) δ = 177.18 (C_k), 176.07 (C_l), 171.48 (C_c), 138.29 (C_g), 137.56 (C_f), 90.01 (C_e), 81.99 (C_h), 63.76 (C_d), 56.97 (C_b), 52.13 (C_i), 50.54 (C_j), 30.97, 30.94 (C_a).
HRMS-ESI(+): 365.9943 $[M+Na]^+$, calculated for $C_{13}H_{14}BrNO_5Na^+$: 365.9948.

3.2.5 Synthesis of S13

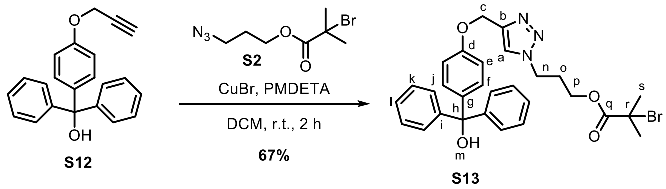

Three sealed 5 mL microwave vials (*A*, *B*, and *C*) were prepared as follows: *A* contained PMDETA (3 mg, 16 µmol, 1.0 eq.) in DCM (3 mL), *B* contained CuBr (2 mg, 16 µmol, 1.0 eq.), and *C* contained **S12** (5 mg, 16 µmol, 1.0 eq.) and **S2** (4 mg, 16 µmol, 1.0 eq.). Vials *B* and *C* were subjected to three N_2/vacuum cycles before use. The solution in vial *A* was degassed by bubbling with N_2 until ~1 mL DCM left before being transferred to vial *B via* cannula; the resulting mixture was stirred until all CuBr had dissolved. The CuBr/PMDETA solution in vial *B* was then transferred to vial *C via* cannula; the resulting reaction mixture was stirred for 2 h at room temperature. The reaction mixture was washed by aqueous EDTA solution (0.25 M, pH 7, 2 x 1 mL) and brine (2 x 1 mL). The organic layer was collected and dried with magnesium sulfate. The mixture was filtered before being concentrated under vacuum. The residue was purified by preparative TLC (500 µm, PE/acetone, 3/1) to yield **S13** as a white powder (6 mg, 11 µmol, 67% yield).

^{1}H NMR (400 MHz, Acetone-d_6, 298 K) δ = 8.09 (s, 1H, H_a), 7.35 – 7.22 (m, 10H, $H_{j,k,l}$), 7.22 – 7.16 (m, 2H, H_f), 7.00 – 6.94 (m, 2H, H_e), 5.20 (s, 1H, H_m), 5.17 (s, 2H, H_c), 4.60 (t, J = 6.9 Hz, 2H, H_n), 4.23 (t, J = 6.1 Hz, 2H, H_p), 2.35 (p, J = 6.5 Hz, 2H, H_o), 1.93 (s, 6H, H_s).
^{13}C NMR (101 MHz, Acetone-d_6, 298 K) δ = 171.75 (C_q), 158.38 (C_d), 149.03, 149.00 (C_i), 144.42 (C_b), 141.34, 141.30 (C_g), 130.16 (C_f), 128.84 (C_j), 128.36 (C_k), 127.55 (C_l), 124.75 (C_a), 114.54 (C_e), 81.78, 81.68 (C_h), 63.51 (C_p), 62.42 (C_c), 57.39 (C_r), 47.53 (C_n), 30.90 (C_s), 29.92 (C_o).

HRMS-ESI(+): 586.1294 [M+Na]$^+$, calculated for $C_{29}H_{30}BrN_3O_4Na^+$: 586.1312.

3.2.6 Synthesis of S14

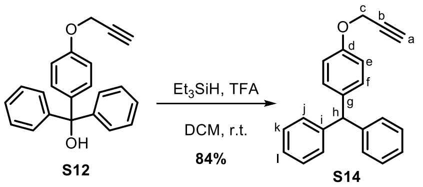

To a solution of **S12** (10 mg, 32 µmol, 1.0 eq.) in DCM (1 mL) was added Et_3SiH (10 µL, 64 µmol, 2.0 eq.) and TFA (5 µL, 64 µmol, 2.0 eq.). The mixture was stirred for 1 h at room temperature before concentrated under vacuum. The residue was purified by preparative TLC (500 µm, PE/Acetone, 4/1) to yield **S14** as a white powder (8 mg, 27 µmol, 84% yield).

^{1}H NMR (500 MHz, Acetone-d_6, 298 K) δ = 7.33 – 7.27 (m, 4H, H_k), 7.24 – 7.19 (m, 2H, H_l), 7.17 – 7.12 (m, 4H, H_j), 7.10 – 7.06 (m, 2H, H_f), 6.97 – 6.92 (m, 2H, H_e), 5.58 (s, 1H, H_h), 4.76 (d, J = 2.4 Hz, 2H, H_c), 3.05 (t, J = 2.4 Hz, 1H, H_a).
^{13}C NMR (126 MHz, Acetone-d_6, 298 K) δ = 157.16 (C_d), 145.29 (C_i), 137.84 (C_g), 131.10 (C_f), 130.11 (C_j), 129.12 (C_k), 127.05 (C_l), 115.46 (C_e), 79.85 (C_b), 76.90 (C_a), 56.70 (C_h), 56.25 (C_c).
HRMS-ESI(+): 321.1247 [M+Na]$^+$, calculated for $C_{22}H_{18}ONa^+$: 321.1250.

3.2.7 Synthesis of S15

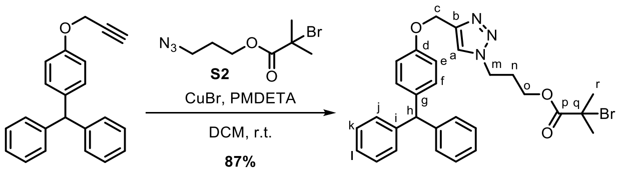

Three sealed 5 mL microwave vials (*A*, *B*, and *C*) were prepared as follows: *A* contained PMDETA (3 mg, 17 µmol, 1.0 eq.) in DCM (3 mL), *B* contained CuBr (2 mg, 17 µmol, 1.0 eq.), and *C* contained **S14** (5 mg, 17 µmol, 1.0 eq.) and **S2** (4 mg, 17 µmol, 1.0 eq.). Vials *B* and *C* were subjected to three N_2/vacuum cycles before use. The solution in vial *A* was degassed by bubbling with N_2 until ~1 mL DCM left before being transferred to vial *B via* cannula; the resulting mixture was stirred until all CuBr had dissolved. The CuBr/PMDETA solution in vial *B* was then transferred to vial *C via* cannula; the resulting reaction mixture was stirred for 2 h at room temperature. The reaction mixture was washed by aqueous EDTA solution (0.25 M, pH 7, 2 x 1 mL) and brine (2 x 1 mL). The organic layer was collected and dried with magnesium sulfate. The mixture was filtered before being concentrated under vacuum. The residue was purified by preparative TLC (500 µm, PE/acetone, 4/1) to yield **S15** as a white powder (8 mg, 15 µmol, 87% yield).

^{1}H NMR (400 MHz, Acetone-d_6, 298 K) δ = 8.08 (s, 1H, H_a), 7.33 – 7.26 (m, 4H, H_k), 7.24 – 7.18 (m, 2H, H_l), 7.17 – 7.11 (m, 4H, H_j), 7.09 – 7.03 (m, 2H, H_f), 7.01 – 6.95 (m, 2H, H_e), 5.57 (s, 1H, H_h), 5.16 (s, 2H, H_c), 4.59 (t, J = 6.9 Hz, 2H, H_m), 4.22 (t, J = 6.1 Hz, 2H, H_o), 2.38 – 2.30 (m, 2H, H_n), 1.93 (s, 6H, H_r).
^{13}C NMR (101 MHz, Acetone-d_6, 298 K) δ = 171.74 (C_p), 158.03 (C_d), 145.37 (C_i), 144.45 (C_b), 137.40 (C_g), 131.14 (C_f), 130.12 (C_j), 129.11 (C_k), 127.04 (C_l), 124.72 (C_a), 115.38 (C_e), 63.51 (C_o), 62.46 (C_c), 57.39 (C_q), 56.71 (C_h), 47.53 (C_m), 30.89 (C_r), 29.92 (C_n).
HRMS-ESI(+): 570.1340 [M+Na]$^+$, calculated for $C_{29}H_{30}BrN_3O_3Na^+$: 570.1363.

4 Synthesis of Polymers

4.1 Representative Procedure for SET-LRP of Methyl Acrylate Using Mechanophore Initiators

Methyl acrylate was filtered through basic alumina to remove the inhibitor prior to use. A stock catalytic solution of Me_6TREN (16 µL, 0.060 mmol) and $CuBr_2$ (5.6 mg, 0.025 mmol) in dry DMSO (1 mL) was prepared. To a 5 mL microwave vial was added the appropriate initiator compound along with catalytic solution, methyl acrylate and dry DMSO. This solution was degassed by bubbling with N_2 for 10 min. A Cu(0) wire wrapped around a stirrer bar, having been cleaned in 12 N HCl for 10 min, was added to the reaction mixture. The reaction mixture was degassed for a further 2 min before being allowed to stir for 15 - 40 min (until the extent of polymerization, as determined approximately by the increasing viscosity of the solution, was deemed acceptable). The solution was added dropwise to a solution of vigorously stirred methanol; the precipitated polymer was recovered and dried under vacuum for two days to yield a white material. Molecular weight and polydispersity indices were determined using an analytical SEC that had been calibrated with polystyrene standards.

4.2 Synthesis of Polymer with Mechanophores

4.2.1 Synthesis of Polymer 1

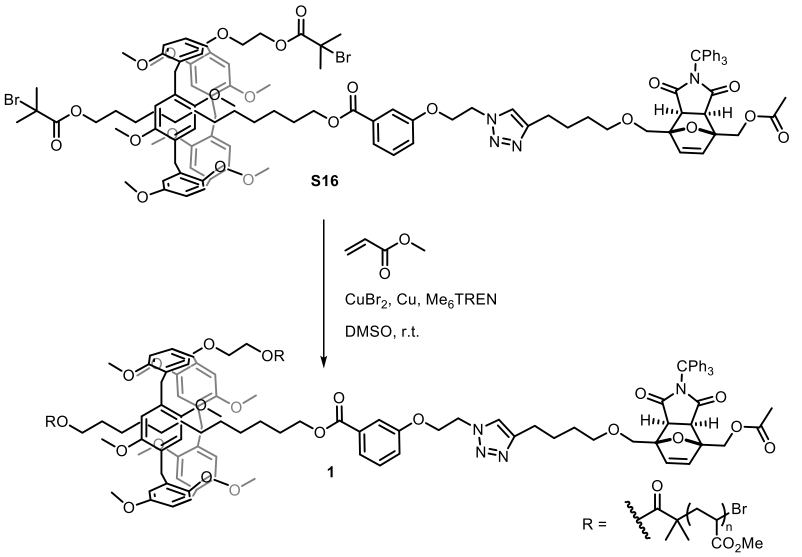

Synthesis followed the representative procedure. **S16** (2.0 mg, 1.0 µmol, 1.0 eq.), 8 µL of catalytic solution ($CuBr_2$: 0.2 µmol, 0.2 eq.; Me_6TREN: 0.5 µmol, 0.5 eq.), methyl acrylate (170 µL, 2.0 mmol, 2000.0 eq.), Cu (0) wire (~3 cm, ~30 mg, 0.5 mmol, ~500.0 eq.) and dry DMSO (170 µL) were used in the reaction to yield polymer **1** (60 mg, M_n = 114 kDa; $Đ$ = 1.17).

4.2.2 Synthesis of Polymer 4

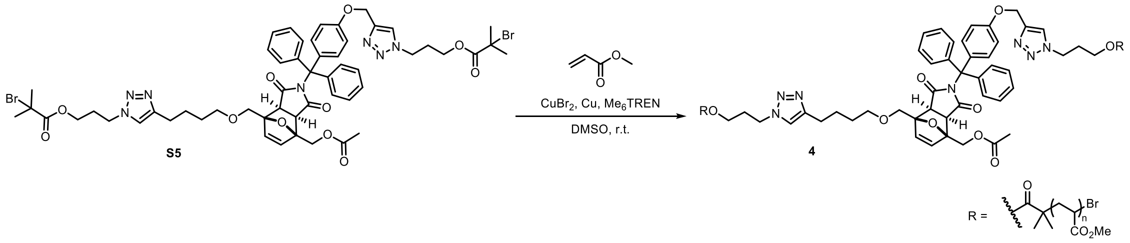

Synthesis followed the representative procedure. **S5** (2.0 mg, 1.8 µmol, 1.0 eq.), 14 µL of catalytic solution ($CuBr_2$: 0.4 µmol, 0.2 eq.; Me_6TREN: 0.8 µmol, 0.5 eq.), methyl acrylate (313 µL, 3.5 mmol, 2000.0 eq.), Cu (0) wire (~3 cm, ~30 mg, 0.5 mmol, ~285.0 eq.) and dry DMSO (313 µL) were used in the reaction to yield polymer **4** (105 mg, M_n = 122 kDa; $Đ$ = 1.19).

4.2.3 Synthesis of Polymer S17

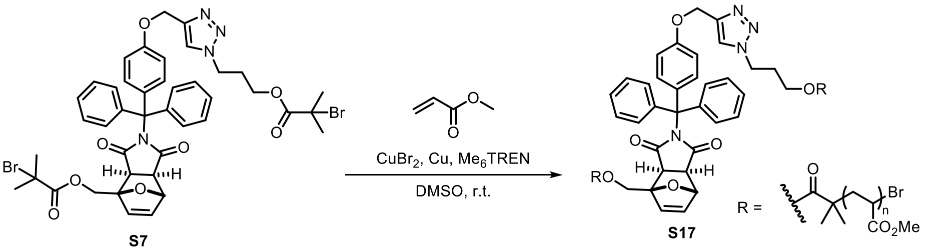

Synthesis followed the representative procedure. **S7** (2.0 mg, 2.2 µmol, 1.0 eq.), 18 µL of catalytic solution ($CuBr_2$: 0.4 µmol, 0.2 eq.; Me_6TREN: 1.1 µmol, 0.5 eq.), methyl acrylate (401 µL, 4.5 mmol, 2000.0 eq.), Cu (0) wire (~3 cm, ~30 mg, 0.5 mmol, ~200.0 eq.) and dry DMSO (401 µL) were used in the reaction to yield polymer **S17** (102 mg, M_n = 118 kDa; $Đ$ = 1.15).

4.3 Synthesis of Reference Polymers

4.3.1 Synthesis of Polymer 5

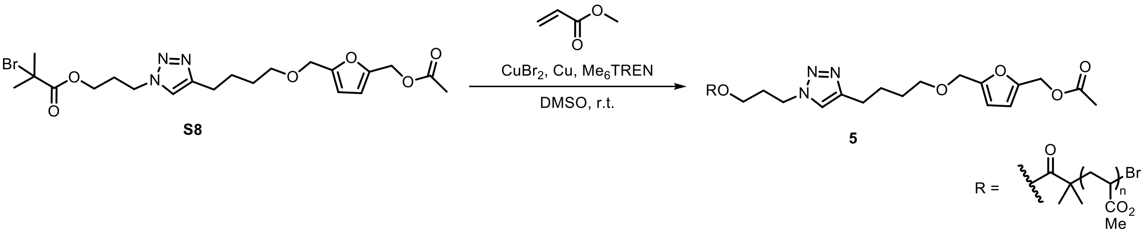

Synthesis followed the representative procedure. **S8** (2.0 mg, 4.0 µmol, 1.0 eq.), 32 µL of catalytic solution ($CuBr_2$: 0.8 µmol, 0.2 eq.; Me_6TREN: 1.9 µmol, 0.5 eq.), methyl acrylate (715 µL, 8.0 mmol, 2000.0 eq.), Cu (0) wire (~3 cm, ~30 mg, 0.5 mmol, ~125.0 eq.) and dry DMSO (715 µL) were used in the reaction to yield polymer **5** (150 mg, M_n = 90 kDa; $Đ$ = 1.18).

4.3.2 Synthetic Route to Polymer 6 and 7

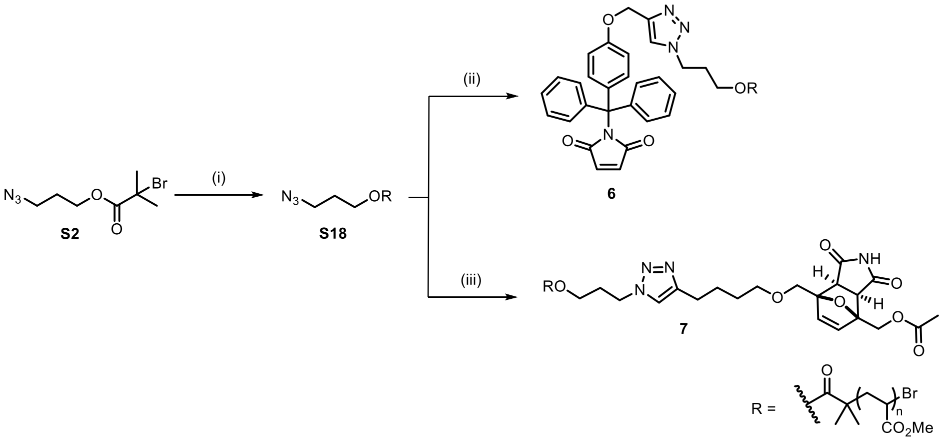

Scheme S3. Synthetic route to polymer **6** and **7**. Conditions: (i) MA, $CuBr_2$, Cu, Me_6TREN, DMSO, r.t.; (ii) **S3**, CuBr, PMDETA, DCM, r.t., 16 h; (iii) **S9**, CuBr, PMDETA, DCM, r.t., 16 h.

4.3.3 Synthesis of Polymer S18

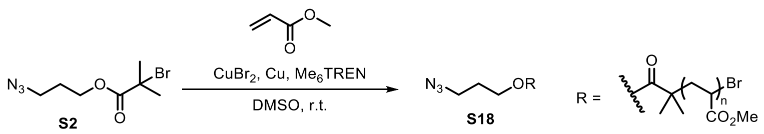

Synthesis followed the representative procedure. **S2** (1.5 mg, 6.0 µmol, 1.0 eq.), 48 µL of catalytic solution ($CuBr_2$: 1.2 µmol, 0.2 eq.; Me_6TREN: 2.9 µmol, 0.5 eq.), methyl acrylate (1075 µL, 12.0 mmol, 2000.0 eq.), Cu (0) wire (~3 cm, ~30 mg, 0.5 mmol, ~80.0 eq.) and dry DMSO (1075 µL) were used in the reaction to yield polymer **S18** (150 mg, M_n = 78 kDa; $Đ$ = 1.20).

4.3.4 Synthesis of Polymer 6

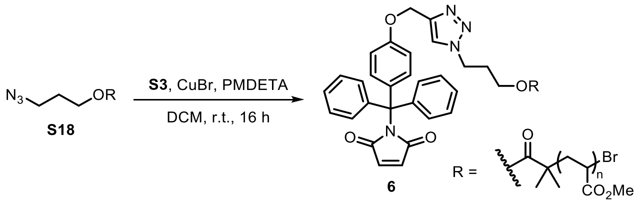

Three sealed 5 mL microwave vials (*A*, *B*, and *C*) were prepared as follows: *A* contained PMDETA (1.0 mg, 5.8 µmol, 15.0 eq.) in DCM (3 mL), *B* contained CuBr (1.1 mg, 7.7 µmol, 20.0 eq.), and *C* contained **S18** (30 mg, 0.4 µmol, 1.0 eq.) and **S3** (0.8 mg, 1.6 µmol, 5.0 eq.). Vials *B* and *C* were subjected to three N_2/vacuum cycles before use. The solution in vial *A* was degassed by bubbling with N_2 until ~1 mL DCM left before being transferred to vial *B via* cannula; the resulting mixture was stirred until all CuBr had dissolved. The CuBr/PMDETA solution in vial *B* was then transferred to vial *C via* cannula; the resulting reaction mixture was stirred for 16 h at room temperature. DCM (25 mL) was added in the reaction mixture before washed by aqueous EDTA solution (0.25 M, pH 7, 2 x 25 mL) and brine (2 x 25 mL). The organic layer was collected and dried with magnesium sulfate. Then the mixture was filtered and evaporated by rotavap in the round bottom flask to make a polymer film. This polymer film was washed by MeOH (3 x 25 mL) and dried under vacuum for 16h to to give polymer **6** (20 mg, M_n = 83 kDa; $Đ$ = 1.16).

4.3.5 Synthesis of Polymer 7

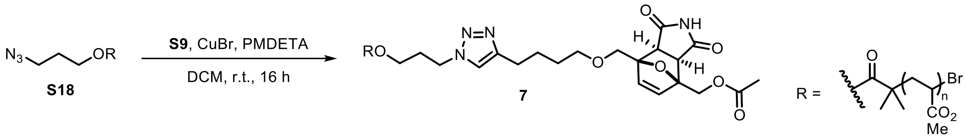

Three sealed 5 mL microwave vials (*A*, *B*, and *C*) were prepared as follows: *A* contained PMDETA (1.3 mg, 7.7 µmol, 15.0 eq.) in DCM (3 mL), *B* contained CuBr (5.8 mg, 10.3 µmol, 20.0 eq.), and *C* contained **S18** (40 mg, 0.5 µmol, 1.0 eq.) and **S9** (0.9 mg, 2.6 µmol, 5.0 eq.). Vials *B* and *C* were subjected to three N_2/vacuum cycles before use. The solution in vial *A* was degassed by bubbling with N_2 until ~1 mL DCM left before being transferred to vial *B via* cannula; the resulting mixture was stirred until all CuBr had dissolved. The CuBr/PMDETA solution in vial *B* was then transferred to vial *C via* cannula; the resulting reaction mixture was stirred for 16 h at room temperature. DCM (25 mL) was added in the reaction mixture before washed by aqueous EDTA solution (0.25 M, pH 7, 2 x 25 mL) and brine (2 x 25 mL). The organic layer was collected and dried with magnesium sulfate. Then the mixture was filtered and evaporated by rotavap in the round bottom flask to make a polymer film. This polymer film was washed by MeOH (3 x 25 mL) and dried under vacuum for 16h to to give polymer **7** (35 mg, M_n = 80 kDa; *Đ* = 1.18).

4.3.6 Synthesis of Polymer 8

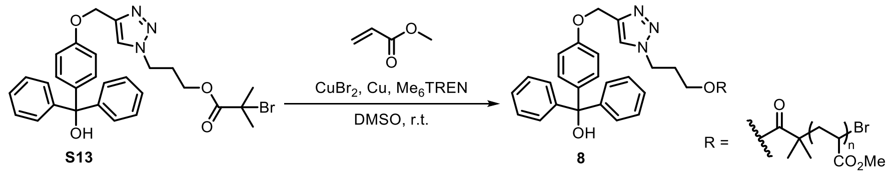

Synthesis followed the representative procedure. **S13** (2.0 mg, 3.6 µmol, 1.0 eq.), 28 µL of catalytic solution ($CuBr_2$: 0.7 µmol, 0.2 eq.; Me_6TREN: 1.7 µmol, 0.5 eq.), methyl acrylate (634 µL, 7.1 mmol, 2000.0 eq.), Cu (0) wire (~3 cm, ~30 mg, 0.5 mmol, ~140.0 eq.) and dry DMSO (634 µL) were used in the reaction to yield polymer **8** (105 mg, M_n = 78 kDa; *Đ* = 1.16).

4.3.7 Synthesis of Polymer S19

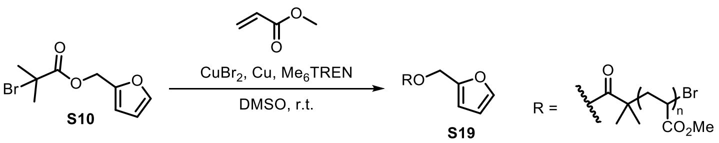

Synthesis followed the representative procedure. **S10** (1.0 mg, 4.1 µmol, 1.0 eq.), 32 µL of catalytic solution ($CuBr_2$: 0.8 µmol, 0.2 eq.; Me_6TREN: 2.0 µmol, 0.5 eq.), methyl acrylate (725 µL, 8.1 mmol, 2000.0 eq.), Cu (0) wire (~3 cm, ~30 mg, 0.5 mmol, ~120.0 eq.) and dry DMSO (725 µL) were used in the reaction to yield polymer **S19** (80 mg, M_n = 54 kDa; *Đ* = 1.14).

4.3.8 Synthesis of Polymer S20

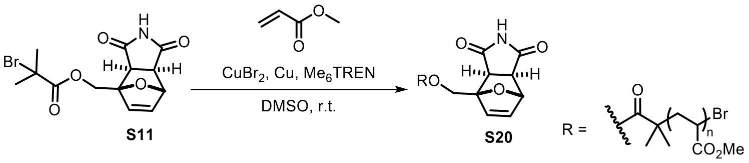

Synthesis followed the representative procedure. **S11** (2.0 mg, 5.8 µmol, 1.0 eq.), 47 µL of catalytic solution ($CuBr_2$: 1.2 µmol, 0.2 eq.; Me_6TREN: 2.8 µmol, 0.5 eq.), methyl acrylate (1040 µL, 11.7 mmol,

2000.0 eq.), Cu (0) wire (~3 cm, ~30 mg, 0.5 mmol, ~80.0 eq.) and dry DMSO (1040 µL) were used in the reaction to yield polymer **S20** (75 mg, M_n = 41 kDa; $Đ$ = 1.15).

4.3.9 Synthesis of Polymer S21

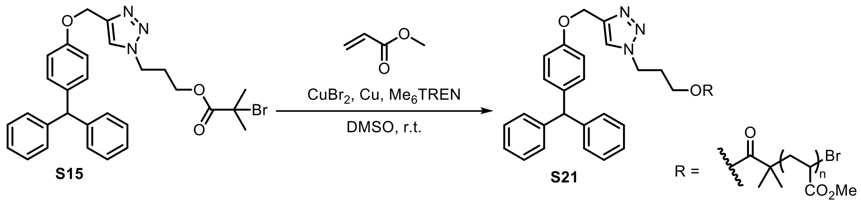

Synthesis followed the representative procedure. **S15** (2.0 mg, 3.7 µmol, 1.0 eq.), 29 µL of catalytic solution ($CuBr_2$: 0.7 µmol, 0.2 eq.; Me_6TREN: 1.8 µmol, 0.5 eq.), methyl acrylate (652 µL, 7.3 mmol, 2000.0 eq.), Cu (0) wire (~3 cm, ~30 mg, 0.5 mmol, ~140.0 eq.) and dry DMSO (652 µL) were used in the reaction to yield polymer **S21** (108 mg, M_n = 81 kDa; $Đ$ = 1.19).

4.4 SEC Data for Synthesised Polymers

Table S1. M_n and $Đ$ values for all synthesised polymers.

Polymer	M_n / kDa	$Đ$
**1**	114	1.17
**4**	122	1.19
**S17**	118	1.15
**5**	90	1.18
**S18**	78	1.20
**6**	83	1.16
**7**	80	1.18
**8**	78	1.16
**S19**	54	1.14
**S20**	41	1.15
**S21**	81	1.19

4.5 SEC Traces for Polymers with Mechanophore

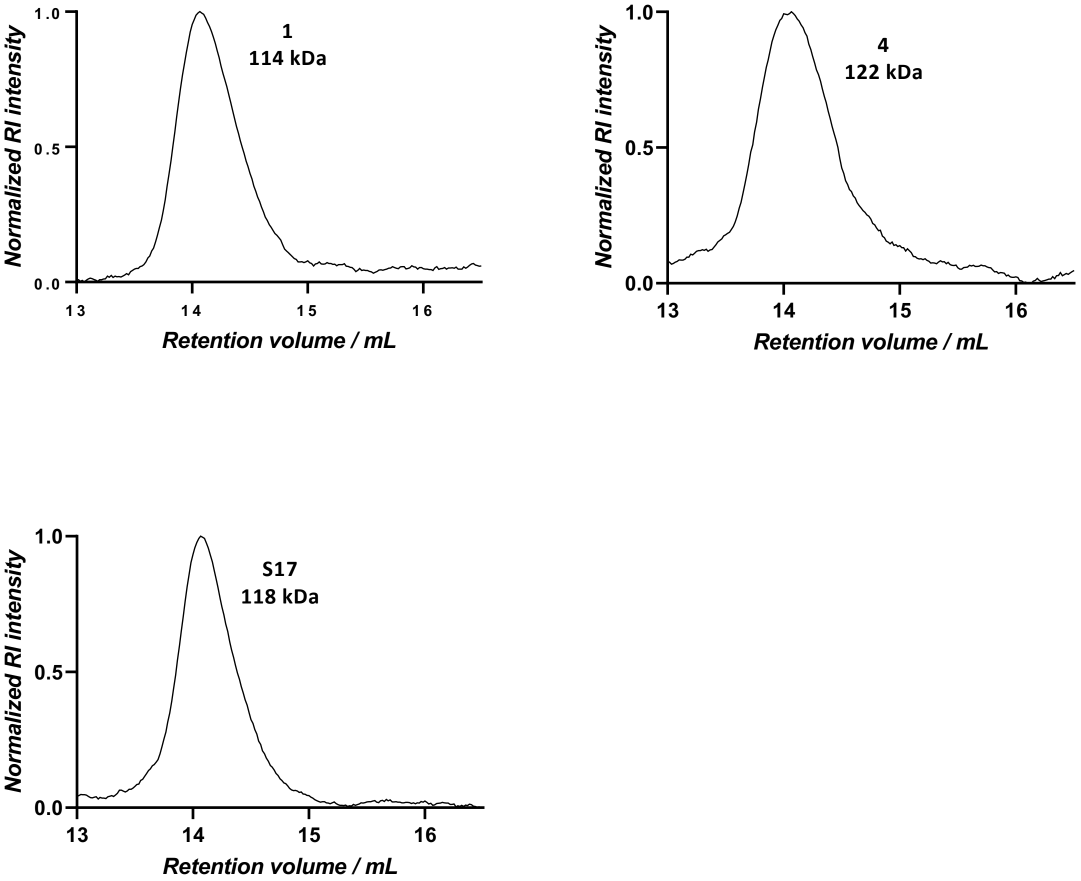

Figure S1. SEC traces for polymers **1**, **4** and **S17**.

4.6 SEC Traces for Reference Polymers

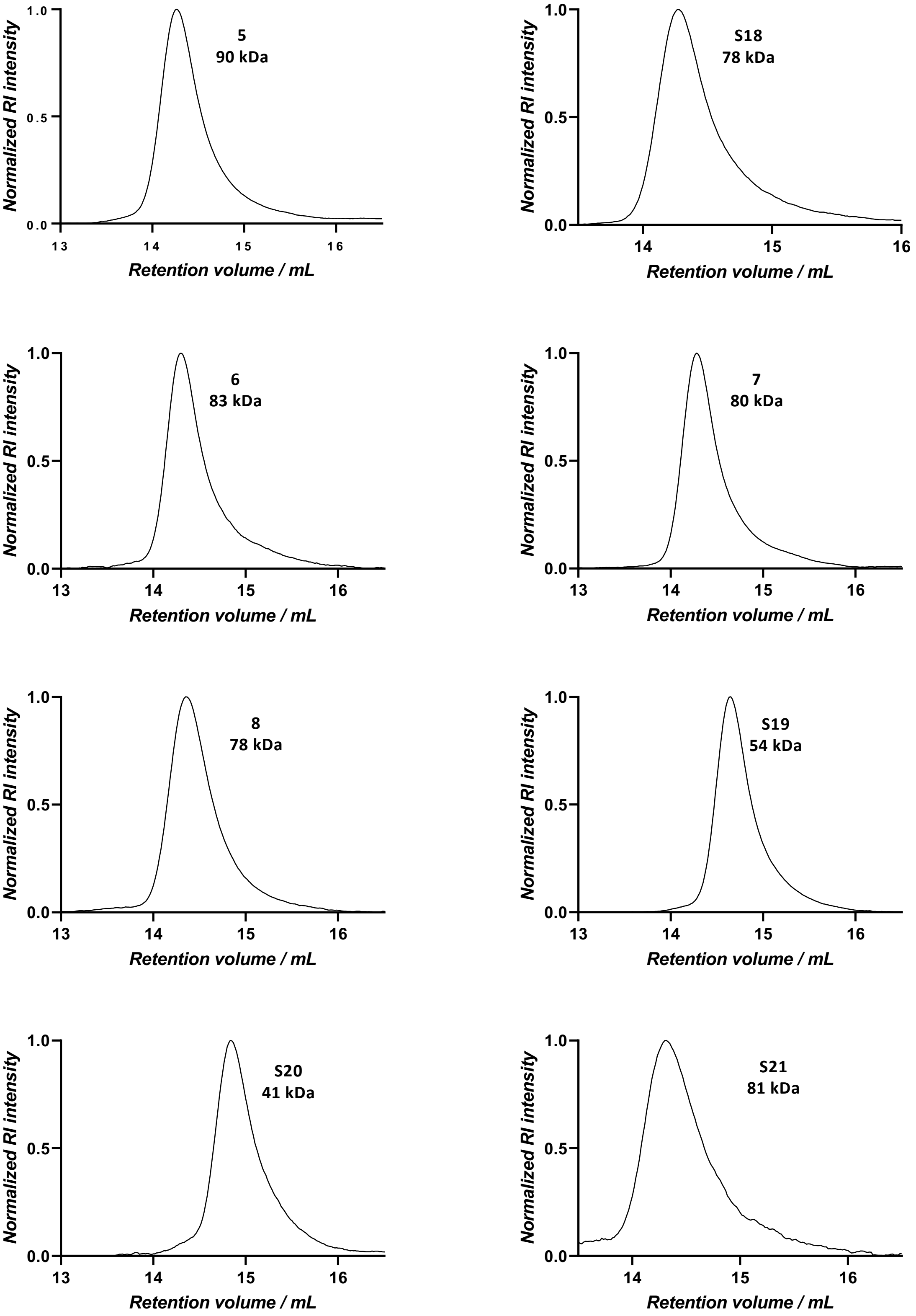

Figure S2. SEC trace of reference polymers **5**, **S18**, **6**, **7**, **8**, **S19**, **S20** and **S21**.

5 Mechanophore Activation via Ultrasound

5.1 General Procedure for Sonication Experiments

The appropriate polymer (15 mg) was added to a Suslick cell and dissolved in the appropriate solvent 15 mL). The solution was degassed by bubbling N_2 through it for a minimum of 10 min prior to the start of sonication; bubbling of N_2 was also maintained throughout the experiment. The Suslick cell was cooled with an ice bath throughout the duration of the sonication to maintain a temperature of ~ 5-10 °C inside the cell. Pulsed ultrasound was applied to the system (1 s ON / 1 s OFF, 25% amplitude (13.0 W cm^{-2}), 20 kHz) for the desired period of time. After sonication, the solvent was evaporated and the polymer was analysed by SEC and NMR spectroscopy. The post-sonication polymer was recovered and washed with MeOH to extract any small molecules not attached to polymer chains. The remaining MeOH-washed polymer and the concentrated MeOH washings were then analysed by NMR spectroscopy.

5.2 Sonication of Polymer 1

Sonication of polymer **1**, using the methodology described in the general procedure (*Section 5.1*) and with the solvent used being acetonitrile or THF/H_2O (75/1), was carried out to determine the extent of activation for retrocycloaddition and C-N bond cleavage in the Diels-Alder structure. SEC analysis of the sonicated polymers showed complete cleavage (M_n of the post-sonication material was less than half of that of the pre-sonication polymer).

Comparison of the ^{1}H NMR spectra of pre- and post-sonication polymer **1** showed that the desired retro-Diels-Alder reaction happened upon mechanical force application; this is evidenced by clear formation of furan species along with the respective maleimide group while the intact Diels-Alder structure clearly decreased in relative intensity. Comparison to reference species also demonstrated that the post-sonication material had some intact rotaxane species remaining. Additionally, the ^{1}H NMR spectrum of the concentrated MeOH washings of polymer **1** sonicated in acetonitrile showed no peaks match with references **S24** and **S25** potentially produced from the heterolytic and homolytic cleavage of C-N bond respectively, indicating only retrocycloaddition occurred after activation. When the sonication was performed in THF/H_2O (75/1), the released maleimide has a diminished olefinic peak, probably due to the addition of radicals generated inside the cavitation bubble (e.g. from water homolysis).

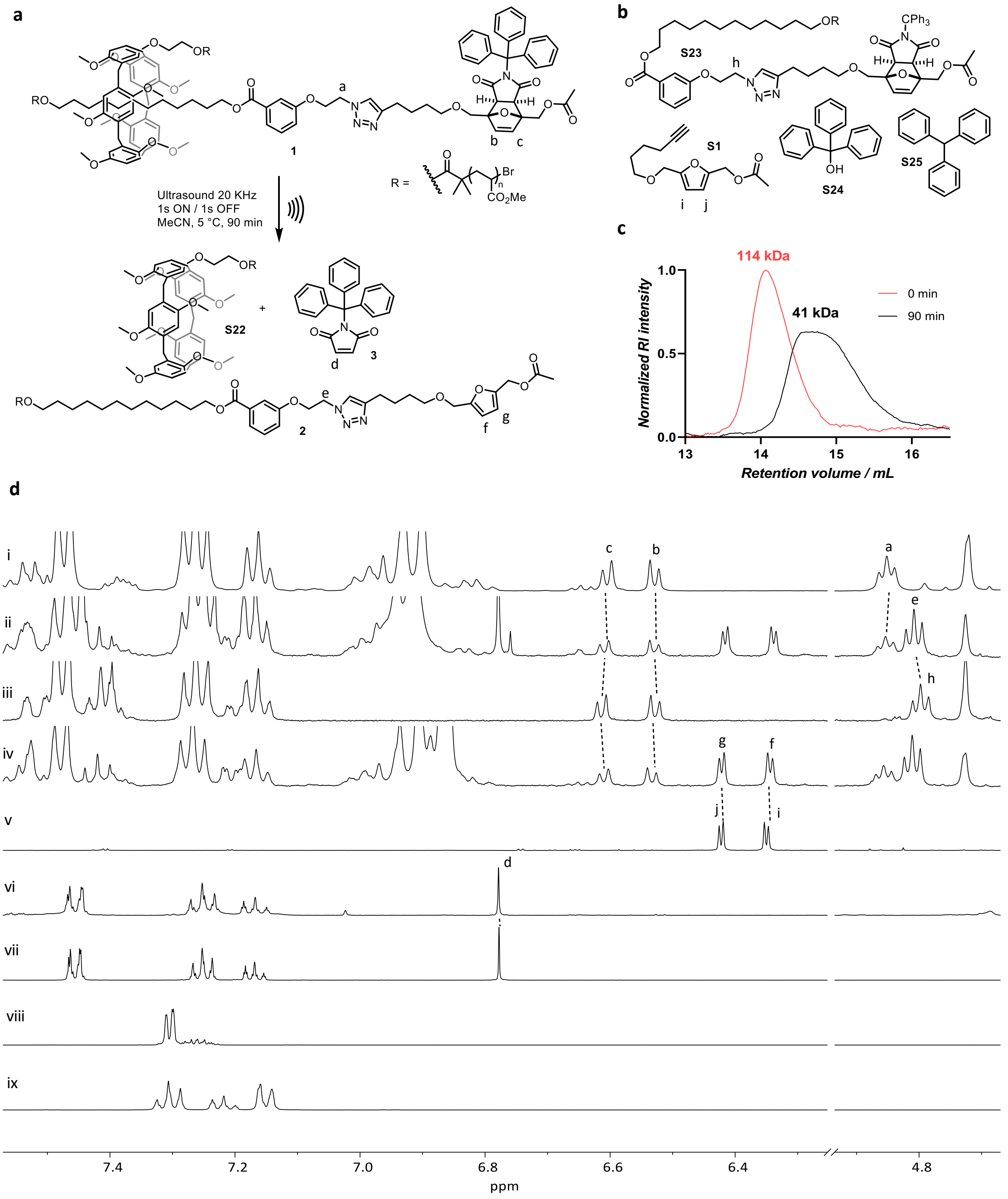

Figure S3. Sonication of polymer **1** in acetonitrile. Sonication of polymer **1** affords polymer fragments **S22** and **2** along with small molecule **3** (a). Reference species **S23**, **S1**, **S24** and **S25** (b). SEC traces of polymer **1** (c) with M_n values before (red) and after (black) sonication. Partial NMR (400 MHz, Acetone-d_6, 298 K) spectra comparison (d) of the pre-sonication polymer **1** (i), post-sonication polymer before being washed with methanol (ii), reference polymer **S23** (iii), post-sonication polymer after being washed with methanol (iv), reference compound **S1** (v), concentrated methanol washings (vi), reference compound **3** (vii), reference compound **S24** (viii), and reference compound **S25** (ix).

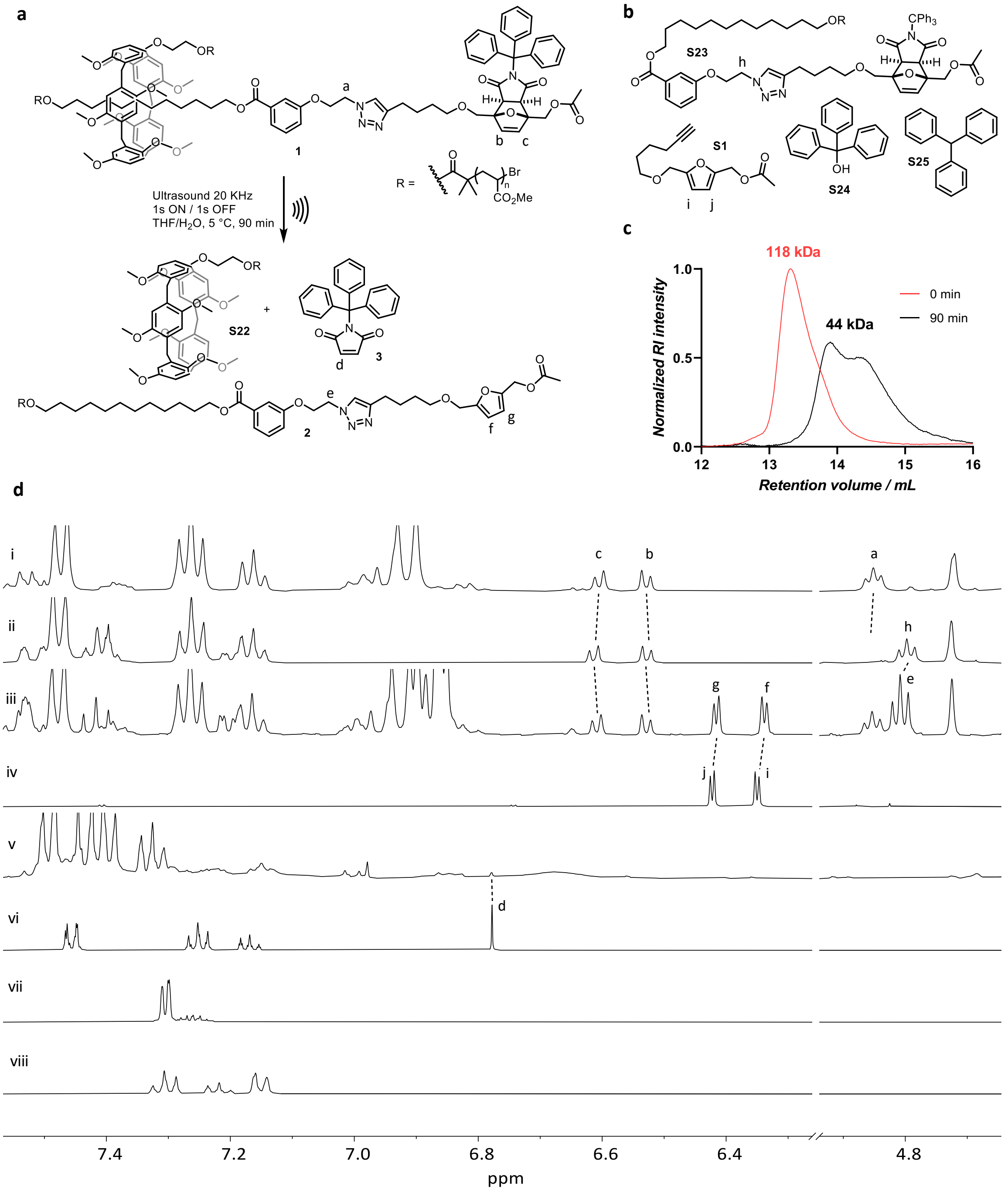

Figure S4. Sonication of polymer **1** in THF/H_2O (75/1). Sonication of polymer **1** affords polymer fragments **S22** and **2** along with small molecule **3** (a). Reference species **S23**, **S1**, **S24** and **S25** (b). SEC traces (column: PL gel 10 µm mixed-C) of polymer **1** (c) with M_n values before (red) and after (black) sonication. Partial NMR (400 MHz, Acetone-d_6, 298 K) spectra comparison (d) of the pre-sonication polymer **1** (i), reference polymer **S23** (ii), post-sonication polymer after being washed with methanol (iii), reference compound **S1** (iv), concentrated methanol washings (v), reference compound **3** (vi), reference compound **S24** (vii), and reference compound **S25** (viii).

5.3 Sonication of Polymer 4

Sonication of polymer **4**, using the methodology described in the general procedure (*Section 5.1*) and with the solvent used being acetonitrile or THF/H_2O (75/1), was carried out to determine the extent of activation for retrocycloaddition and C-N bond cleavage in the Diels-Alder structure. SEC analysis of the sonicated polymers showed complete cleavage (M_n of the post-sonication material was less than half of that of the pre-sonication polymer).

Comparison of the ^{1}H NMR spectra of pre- and post-sonication polymer **4** showed that both of retro-Diels-Alder reaction and cleavage of C-N bond happened upon mechanical force application. The retro-Diels-Alder reaction is evidenced by clear formation of furan species along with the respective maleimide group while the intact Diels-Alder structure clearly decreased in relative intensity. The peaks near by the olefinic peaks of Diels-Alder adduct confirmed the cleavage of C-N bond after comparing with peaks (*i*, *j*) in reference polymer **7**. Additionally, trityl alcohol peaks (*m*-*p*) rather than triphenylmethane peaks (*s*-*u*) were found in the ^{1}H NMR spectra of post-sonication polymer **4**, indicating the heterolytic scission of C-N bond.

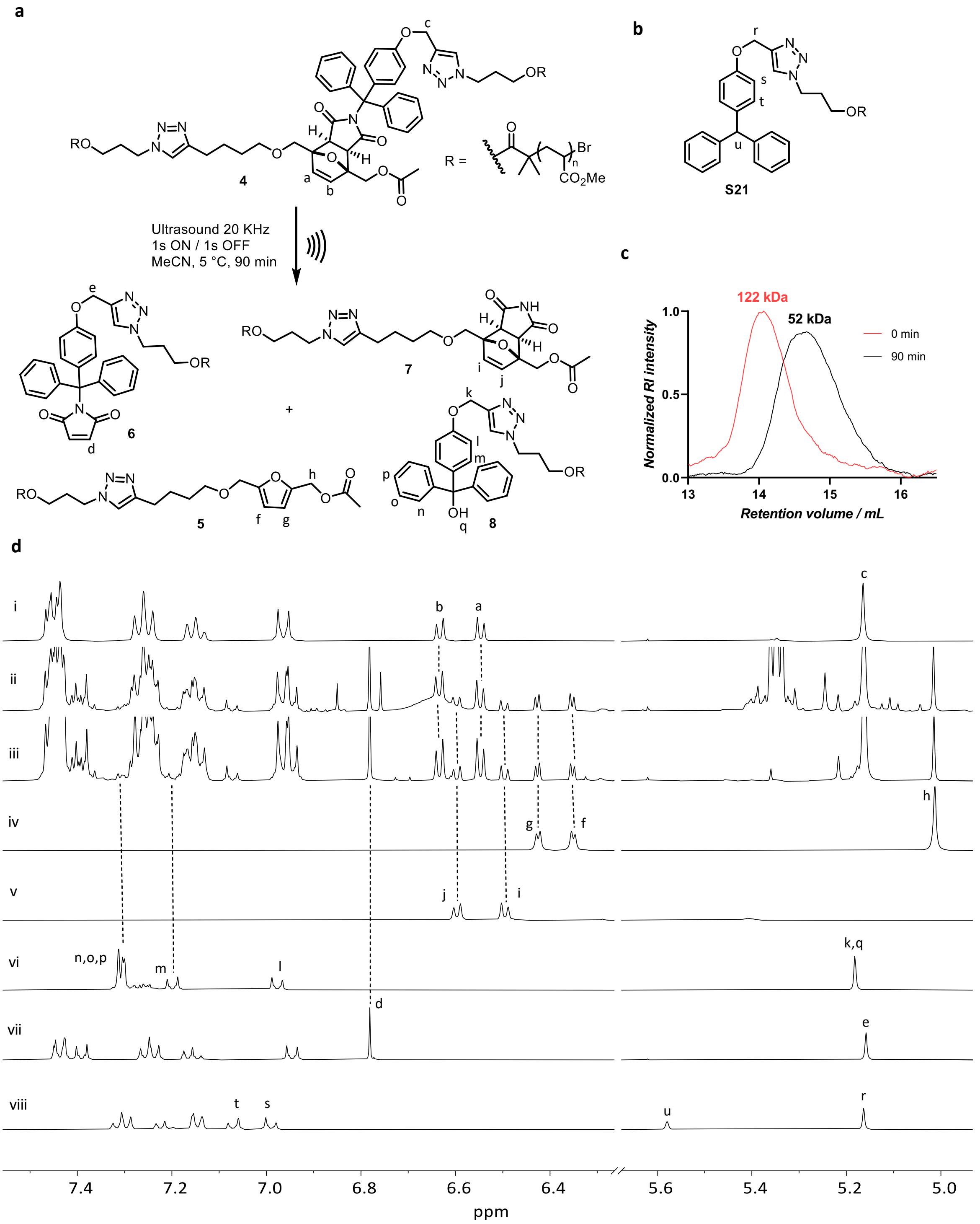

Figure S5. Sonication of polymer **4** in acetonitrile. Sonication of polymer **4** affords polymer fragments **5**, **6**, **7** and **8** (a). Reference species **S21** (b). SEC traces of polymer **4** (c) with M_n values before (red) and after (black) sonication. Partial NMR (400 MHz, Acetone-d_6, 298 K) spectra comparison (d) of the pre-sonication polymer **4** (i), post-sonication polymer before being washed with methanol (ii), post-sonication polymer after being washed with methanol (iii), reference polymer **5** (iv), reference compound **7** (v), reference compound **8** (vi), reference compound **6** (vii) and reference compound **S21** (viii).

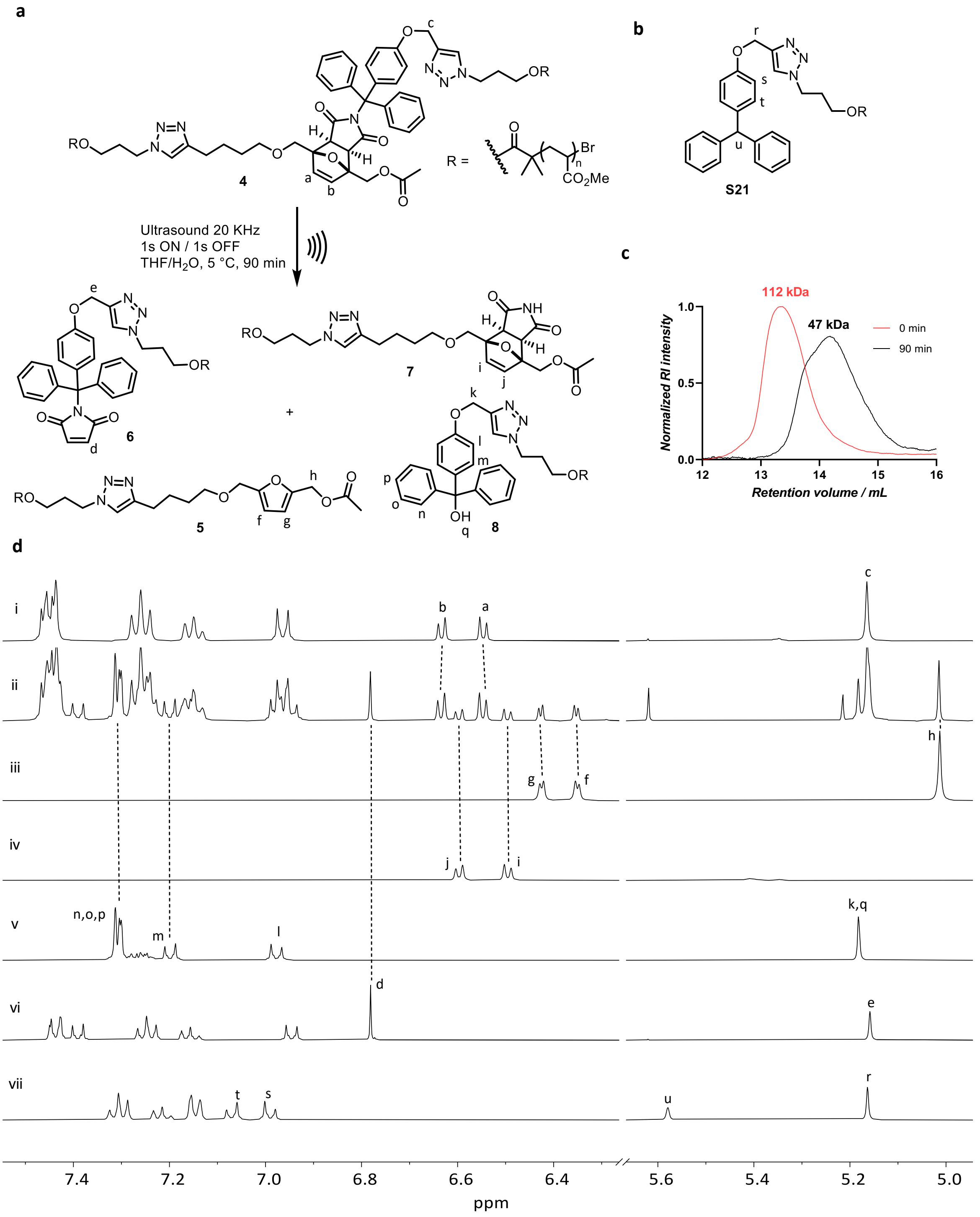

Figure S6. Sonication of polymer **4** in THF/H_2O (75/1). Sonication of polymer **4** affords polymer fragments **5**, **6**, **7** and **8** (a). Reference species **S21** (b). SEC traces (column: PL gel 10 µm mixed-C) of polymer **4** (c) with M_n values before (red) and after (black) sonication. Partial NMR (400 MHz, Acetone-d_6, 298 K) spectra comparison (d) of the pre-sonication polymer **4** (i), post-sonication polymer after being washed with methanol (ii), reference polymer **5** (iii), reference compound **7** (iv), reference compound **8** (v), reference compound **6** (vi) and reference compound **S21** (vii).

5.4 Sonication of Polymer S17

Sonication of polymer **S17**, using the methodology described in the general procedure (*Section 5.1*) and with the solvent used being acetonitrile, was carried out to determine the extent of activation for retrocycloaddition and C-N bond cleavage in the Diels-Alder structure. SEC analysis of the sonicated polymers showed complete cleavage (M_n of the post-sonication material was less than half of that of the pre-sonication polymer).

In the sonication experiment, polymer **S17** lacking the terminal -CH_2OAc group on DA adduct showed similar dissociation to polymer **4**: retro-Diels-Alder reaction and heterolytic cleavage of C-N bond but more retrocycloaddition and less unselective cleavage of polymer chain (*Table S3*).

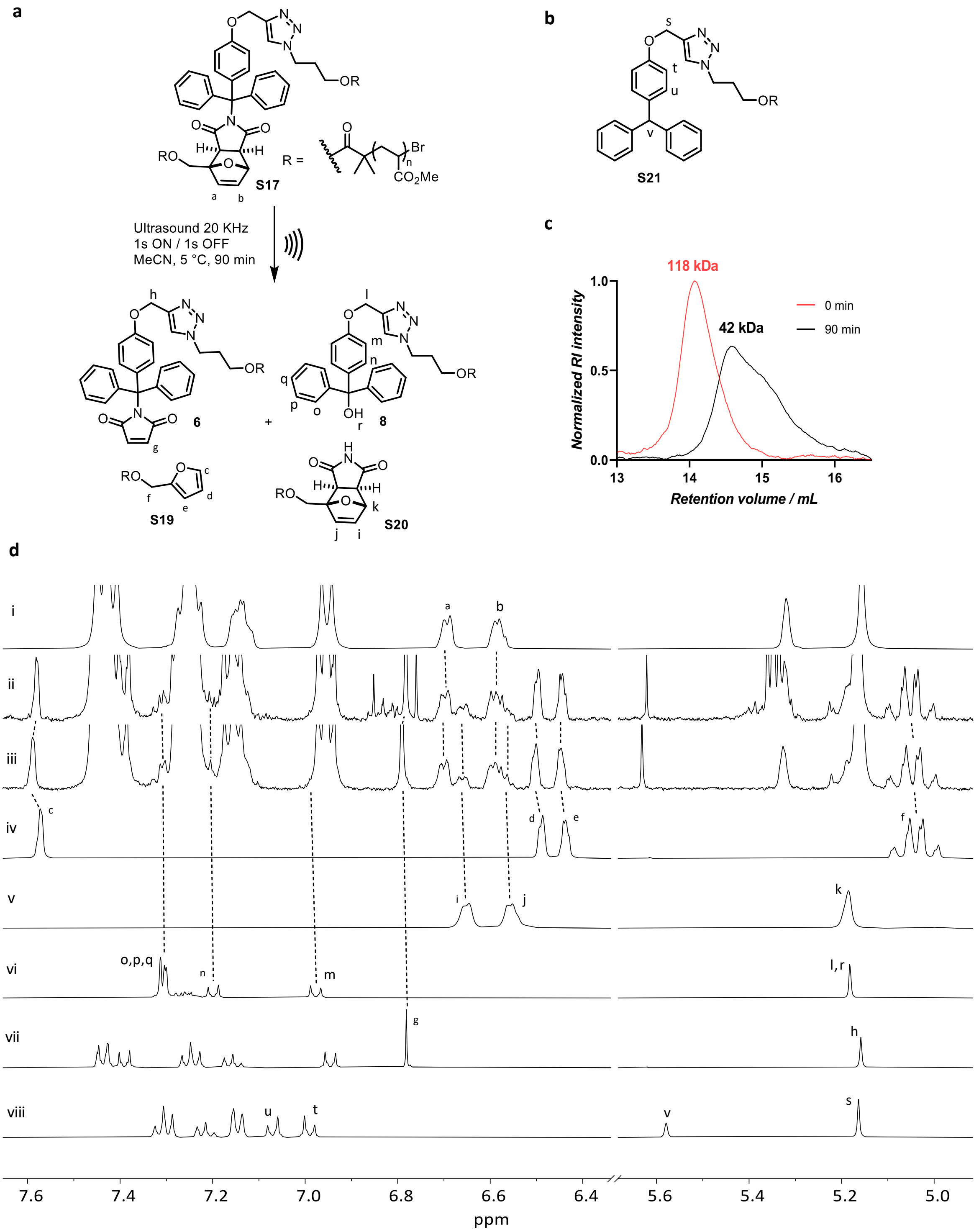

Figure S7. Sonication of polymer **S17** in acetonitrile. Sonication of polymer **S17** affords polymer fragments **S19**, **6**, **S20** and **8** (a). Reference species **S21** (b). SEC traces of polymer **S17** (c) with M_n values before (red) and after (black) sonication. Partial NMR (400 MHz, Acetone-d_6, 298 K) spectra comparison (d) of the pre-sonication polymer **S17** (i), post-sonication polymer before being washed with methanol (ii), post-sonication polymer after being washed with methanol (iii), reference polymer **S19** (iv), reference compound **S20** (v), reference compound **8** (vi), reference compound **6** (vii) and reference compound **S21** (viii).

5.5 Kinetic Investigation Overview

Sonications were carried out in acetonitrile following the general procedure (*Section 5.1*). Aliquots of 200 µL were taken at sonication times of: 0, 5, 10, 15, 20, 25, 30, 35, 40, 50, 60 and 90 min. The solvent was evaporated off under a stream of N_2 and redissolved in 700 µL of THF. The sample was filtered through a syringe filter (PTFE, 0.45 µm pore size) and analysed by SEC. Upon sonication, the average molecular weight of the sample tends toward around 45 kDa and the apparent rate constant (k*) can be derived from first five points with Nalepa's method (**Table S2**, **Figure S6-S9**).[5]

Table S2. M_n and k* values for polymers **1**, **4** and **S17**.

**Polymer**	**M_n/ kDa**	**k*/ $min^{-1}.kDa^{-1}.10^5$**			
		Run 1	Run 2	Run 3	Average
**1**	114	28.2	24.8	26.3	26.4
**4**	122	24.3	25.0	25.2	24.8
**S17**	118	22.0	21.5	22.6	22.0

Note: First five points were used for the calculation of k* due to the secondary cleavage after 20 min sonication time.

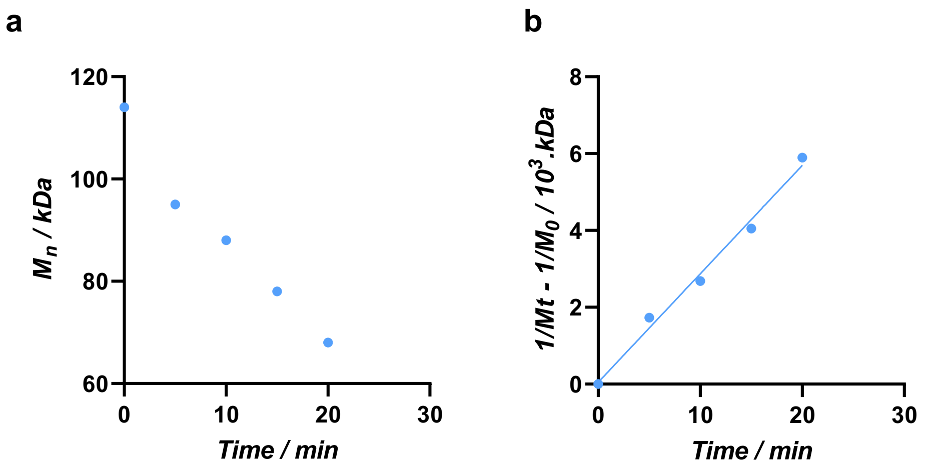

Figure S8. Kinetics of polymer **1**, showing M_n decay over the course of the sonication (a) and the determination of the apparent rate constant k* (b).

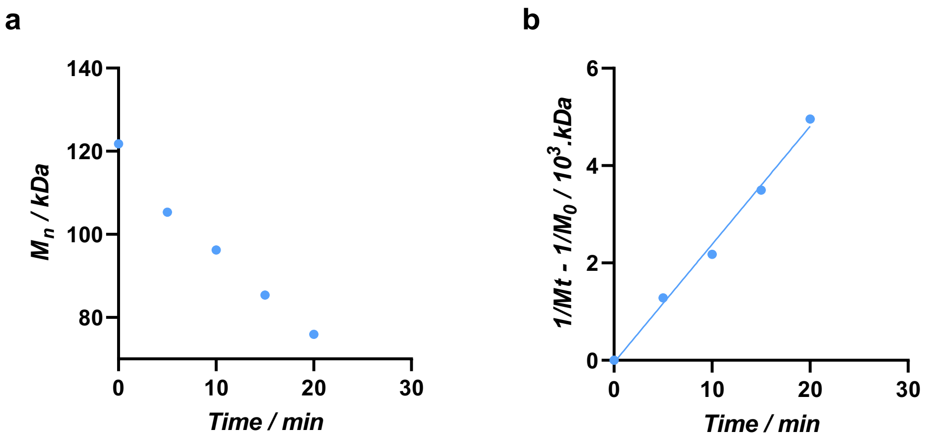

Figure S9. Kinetics of polymer **4**, showing M_n decay over the course of the sonication (a) and the determination of the apparent rate constant *k** (b).

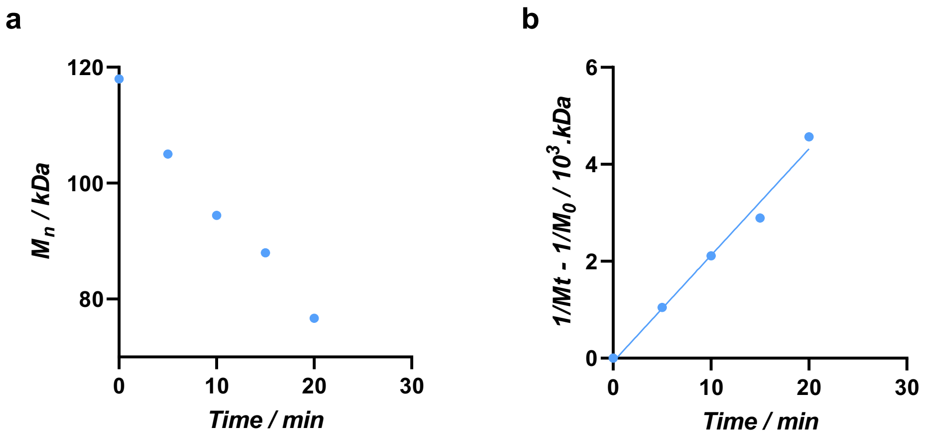

Figure S10. Kinetics of polymer **S17**, showing M_n decay over the course of the sonication (a) and the determination of the apparent rate constant *k** (b).

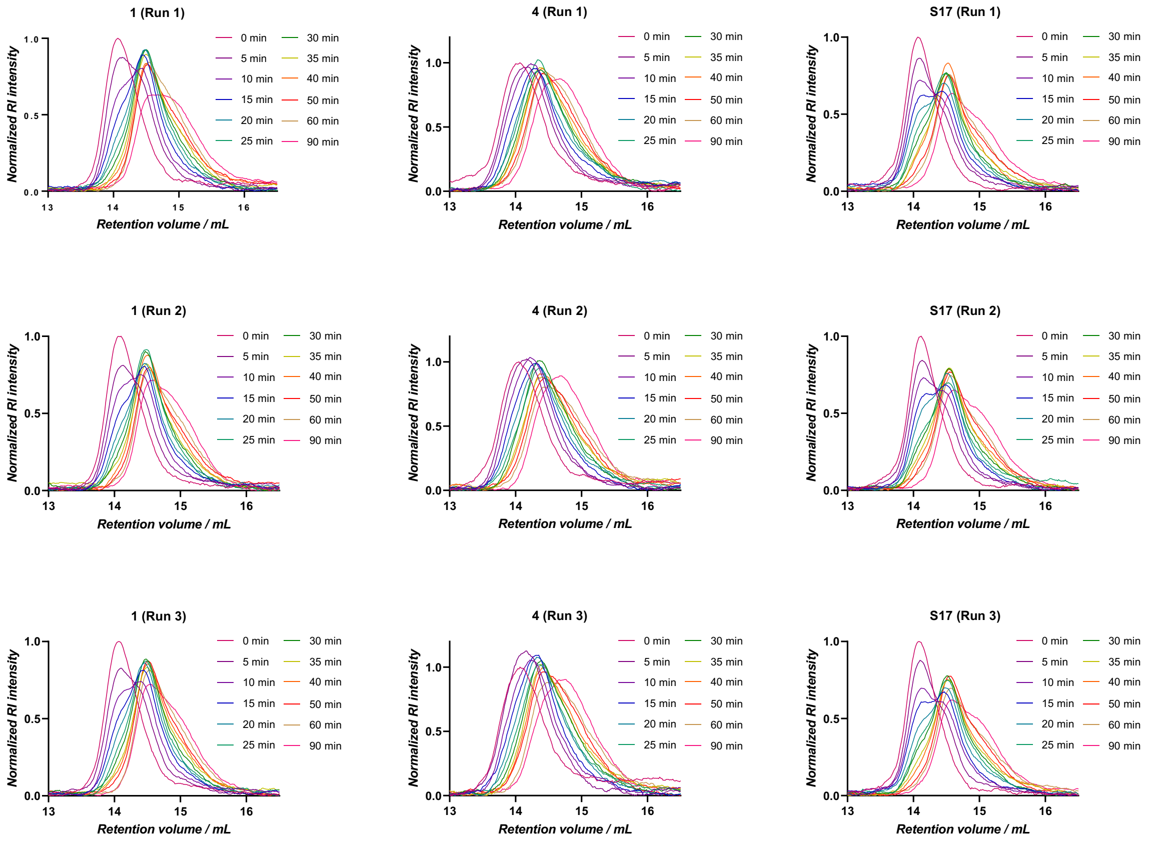

Figure S11. Kinetics of polymer **1**, **4** and **S17** carried out in triplicate, showing area-normalised response traces.

6 Calculation of Extent of Mechanophore Activation

6.1 Calculations for Polymer 1

Here we used polymer **1** sonicated in acetonitrile as an example of how we calculated the extent of activation of retrocycloaddition for polymer with rotaxane actuator. No C-N bond cleavage was found in this mechanophore after the activation.

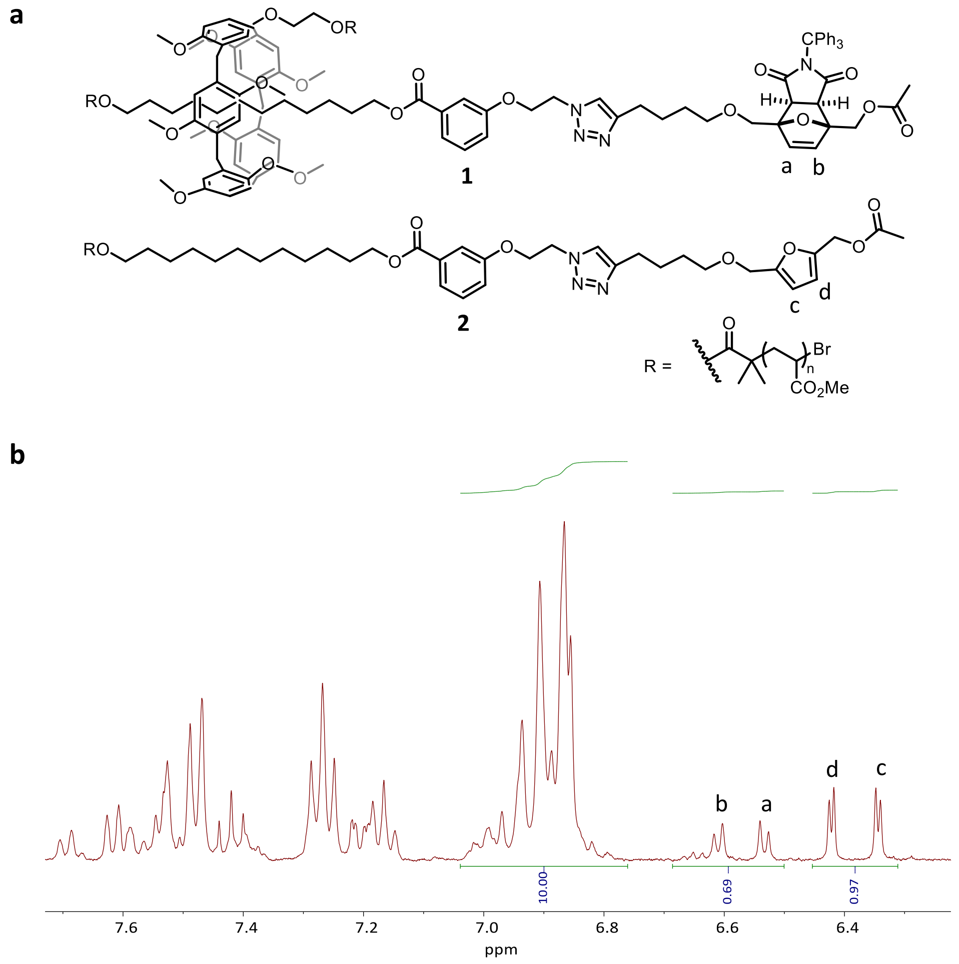

Figure S12. Polymer **1** affords polymer fragment **2** after activation (a). And, partial ^{1}H NMR (400 MHz, Acetone-d_6, 298 K) spectra of post-sonication polymer **1** after being washed with methanol (b).

Here, we use values determined from integration of peaks a and b ($I_{a,b}$), c and d ($I_{c,d}$) in the ^{1}H NMR spectra of post-sonication polymer **1** after being washed with methanol.

The extent of retro-Diels-Alder reaction (C_r) having occurred during the sonication was determined by the formula below:

$$C_r = \frac{I_{c,d}}{I_{a,b} + I_{c,d}} \times 100\%$$

In this case, $\boldsymbol{C_r}$ **= 58%** [(0.97 / (0.69 + 0.97)) × 100%].

6.2 Calculations for Polymers 4 and S17

Here we used polymer **4** sonicated in acetonitrile as an example of how we calculated the extent of activation of retrocycloaddition and C-N bond cleavage for polymers **4** and **S17**.

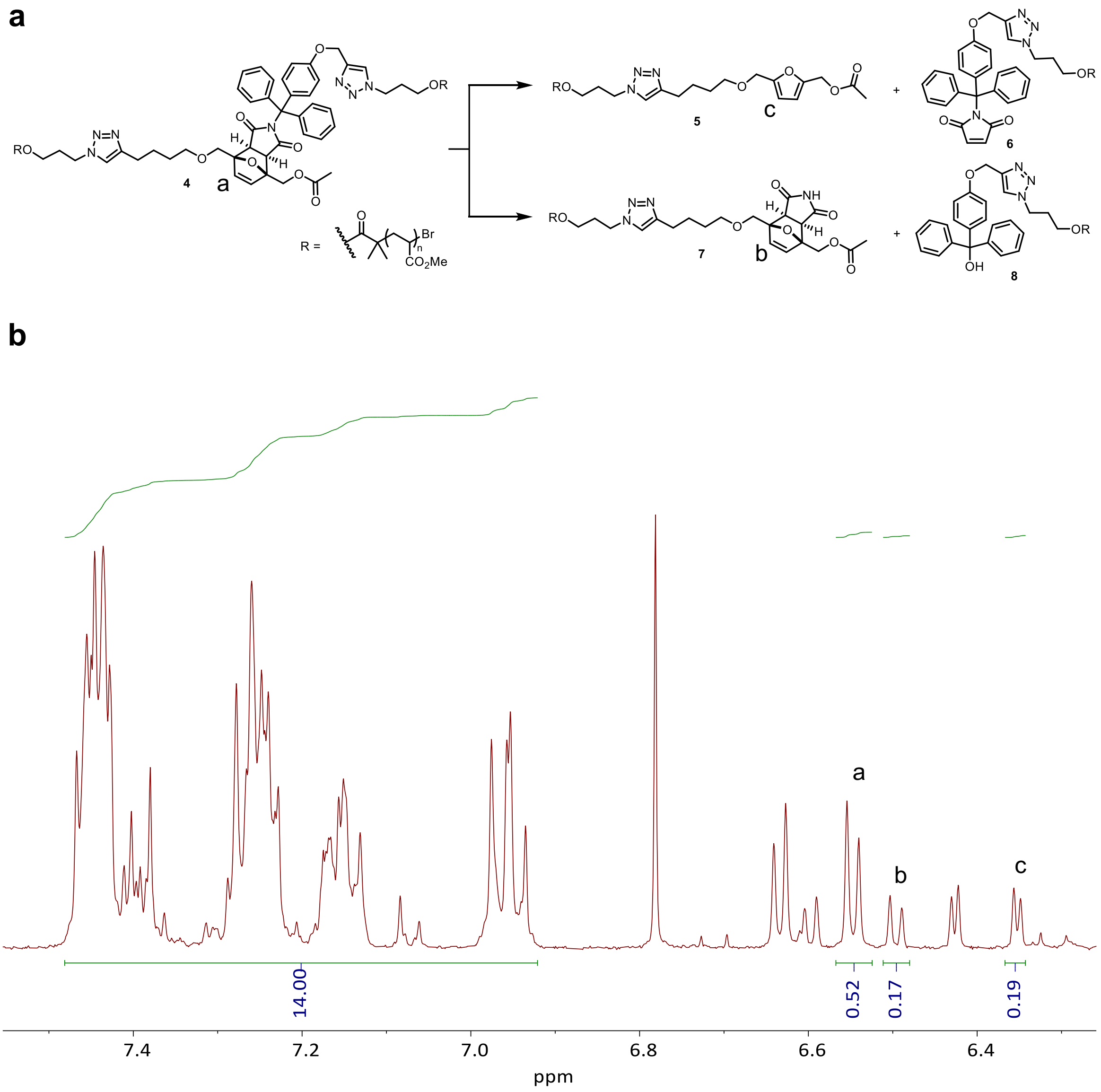

Figure S13. Polymer **4** affords polymer fragments **5**, **6**, **7** and **8** after activation (a). And partial ^{1}H NMR (400 MHz, Acetone-d_6, 298 K) spectra of post-sonication polymer **4** after being washed with methanol (b).

Here, we use values determined from integration of peak a (I_a), b (I_b), and c (I_b) in the ^{1}H NMR spectra of post-sonication polymer **4** after being washed with methanol.

The extent of retro-Diels-Alder reaction (C_r) and C-N bond cleavage (C_{CN}) having occurred during the sonication was determined by the formula below:

$$C_r = \frac{I_c}{I_a + I_b + I_c} \times 100\%$$

$$C_{CN} = \frac{I_b}{I_a + I_b + I_c} \times 100\%$$

In this case, $\boldsymbol{C_r}$ **= 22%** [(0.19 / (0.52 + 0.17 + 0.19)) × 100%] and $\boldsymbol{C_{CN}}$ **= 19%** [(0.17 / (0.52 + 0.17 + 0.19)) × 100%].

6.3 Summary of Mechanophores Activated by Sonication

Table S3. Analysis of mechanical activation *via* ultrasound of polymers **1, 4** and **S17** in acetonitrile.

**No.**	**Polymer**	**Geometry**	**Pre-sonication**		**Post-sonication**								
			**M_n (kDa)**	**Đ**	**M_n (kDa)**	**Đ**	**k*/ $min^{-1}.kDa^{-1}.10^5$**				**Conversion of retro-DA reaction (%)**	**Conversion of cleavage of NC bond (%)**	**Intact mechanophore[a] (%)**
							**Run 1**	**Run 2**	**Run 3**	**Average**			
**1**	**1**	Interlocked	114	1.17	41	1.35	28.2	24.8	26.3	26.4	58	0	42
**2**	**4**	Non-interlocked	122	1.19	52	1.29	24.3	25.0	25.2	24.8	22	19	59
**3**	**S17**		118	1.15	42	1.36	22.0	21.5	22.6	22.0	40	18	42

Table S4. Analysis of mechanical activation *via* ultrasound of polymers **1** and **4** in THF/H_2O (75/1).

**No.**	**Polymer**	**Geometry**	**Pre-sonication**		**Post-sonication**				
			**M_n (kDa)**	**Đ**	**M_n (kDa)**	**Đ**	**Conversion of retro-DA reaction (%)**	**Conversion of cleavage of NC bond (%)**	**Intact mechanophore[a] (%)**
**1**	**1**	Interlocked	118	1.14	44	1.28	58	0	42
**2**	**4**	Non-interlocked	112	1.25	47	1.26	27	20	53

Notes:
a) This value is given as the remaining percentage that is unaccounted for those that undergo the desired activation.

Table S5. Pairwise comparison in t-test of the apparent rate constant (k*) of polymers **1, 4** and **S17**.

	**1**	**4**	**S17**
**1**	-	-	-
**4**	No significant difference (P > 0.05)	-	-
**S17**	Significant difference (P <0.05)	Significant difference (P <0.05)	-

7 CoGEF Calculations

7.1 General method

CoGEF calculations were performed on Spartan'20 following Beyer's method.[6] The structure of the mechanophore was built in Spartan'20 and minimized using molecular mechanics (MMFF). The distance between the terminal methyl groups was constrained and increased by increments of 0.1 Å and/or 1.0 Å depending on the mechanophore. At each step, the energy was minimized by molecular mechanics (MMFF) then DFT (B3LYP/6-31G*) in vaccum, unless otherwise stated. The relative energy of each intermediate was determined by setting the energy of the initial state at 0 kJ/mol. F_{max} values were determined from the slope of the final 3 points.

7.2 CoGEF of linear models (pulling)

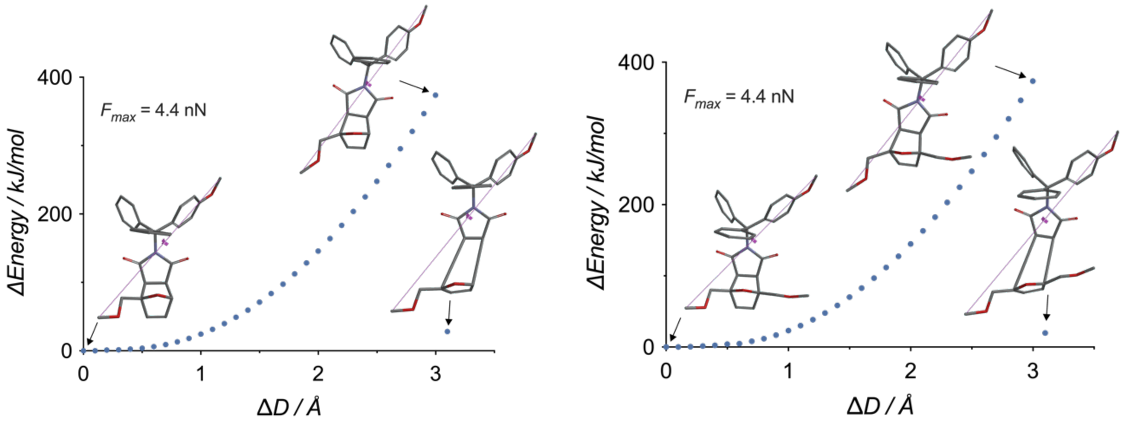

Figure S14. Evolution of energy upon simulated elongation (CoGEF, DFT B3LYP/6-31G*) of models of the DA adducts activated by pulling.

7.3 CoGEF of rotaxane models (pulling)

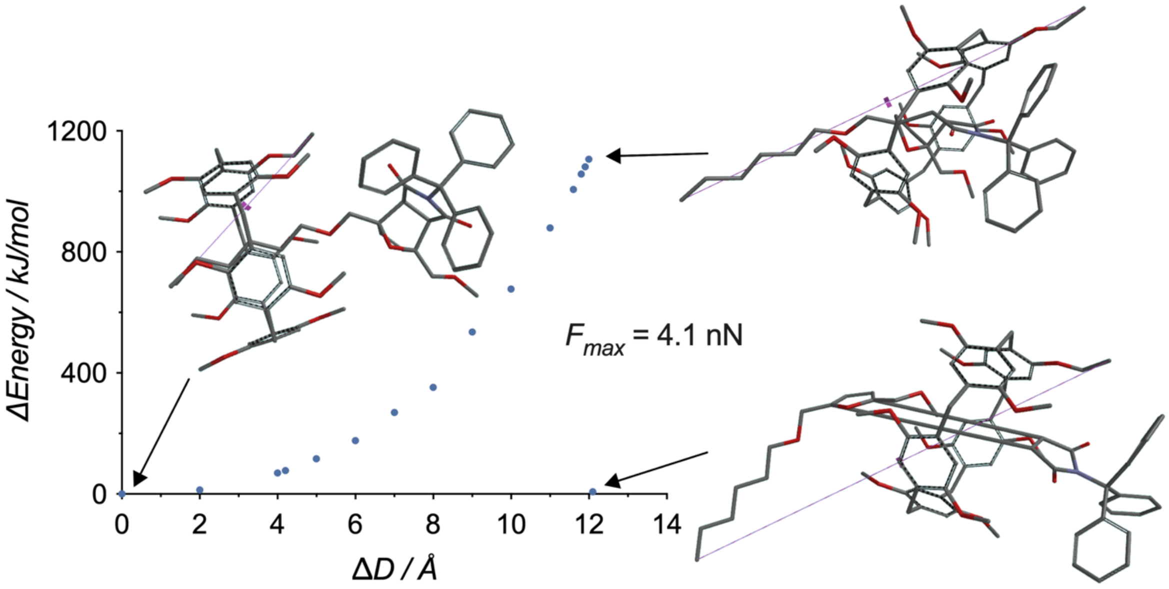

Figure S15. Evolution of energy upon simulated elongation (CoGEF, DFT B3LYP/6-31G*) of a model of the DA adduct activated by pushing (rotaxane).

7.4 Heterolytic cleavage of C-N bond in a distal-exo model

The maleimide and trityl alcohol products observed during the sonication of the linear adduct (Figure 1) suggest that the C-N bond scission proceeds via a heterolytic mechanism whereby the generated maleimide anion and trityl cation react with water. This hypothesis is supported by the simulated elongation of the corresponding *distal-exo* adduct, in which the rDA reactivity is suppressed (Figure S16 and Table S6).[3]

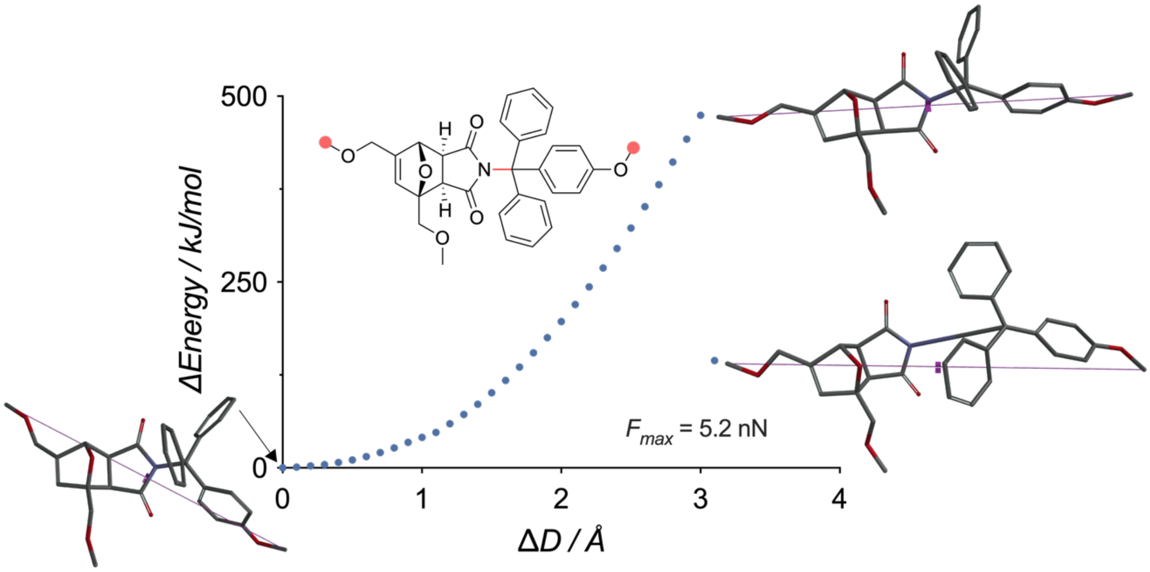

Figure S16. Evolution of energy upon simulated elongation (CoGEF, DFT ωB97X/6-31G*, CPCM dielectric = 7.43 (THF)) of a *distal-exo* adduct in wich the rDA reactivity is suppressed, leading to the scission of the C-N bond.

Table S6. Natural charges at C and N, and orbitals for the broken model of the *distal-exo* adduct, the maleimide anion and radicals, and trityl cation and radical models (DFT ωB97X-D/6-31G*, CPCM dielectric = 7.43 (THF)) indicate a heterolytic scission of the C-N bond.

**HOMO**		
Distal-exo after scission	Maleimide anion	Maleimide radical
Natural charge N = **-0.746**	*Natural charge N* = **-0.731**	*Natural charge N* = **-0.274**
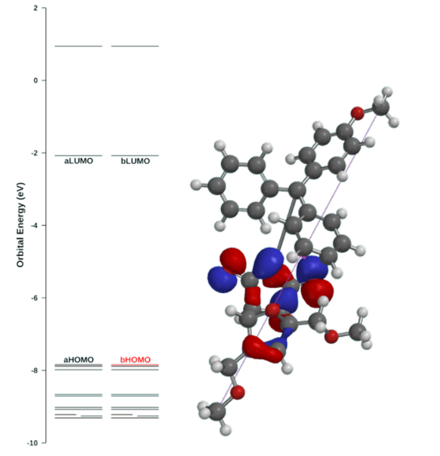	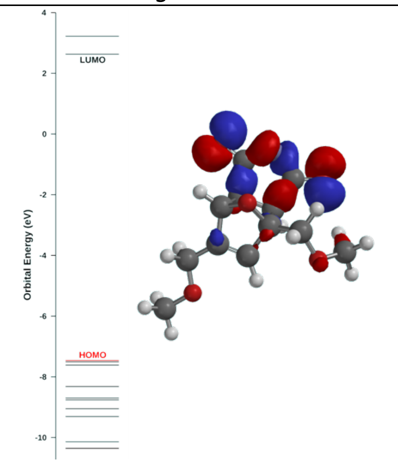	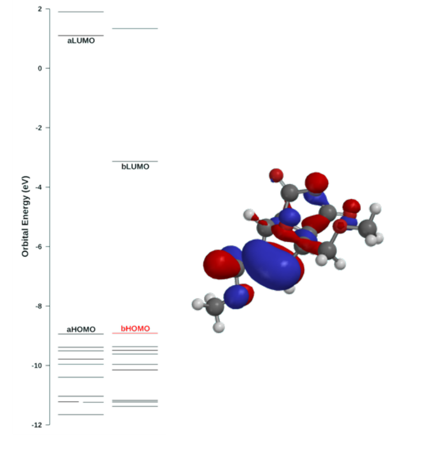

**LUMO**		
Distal-exo after scission	Trityl cation	Trityl radical
Natural charge at C = **0.255**	*Natural charge at C* = **0.251**	*Natural charge at C* = **0.032**
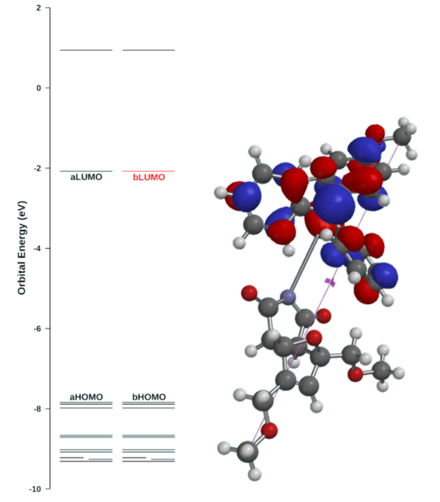	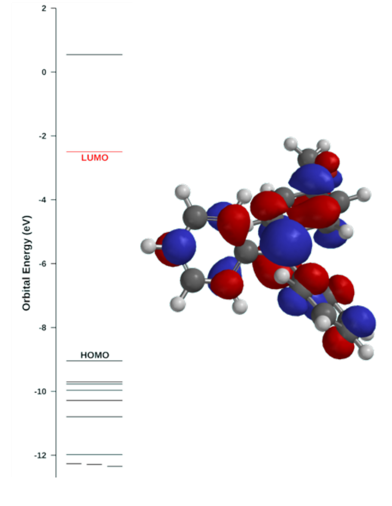	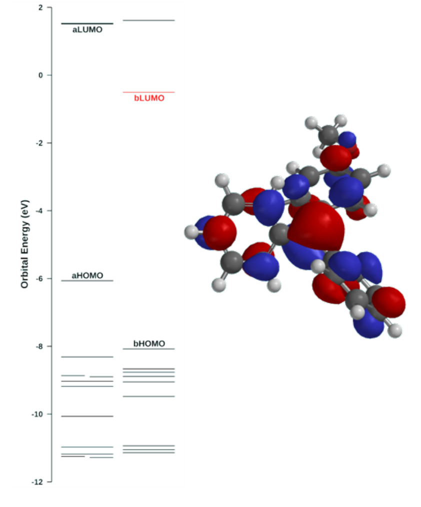

8 NMR Spectra

8.1 Small Molecule NMR Spectra

8.1.1 Spectra of S4

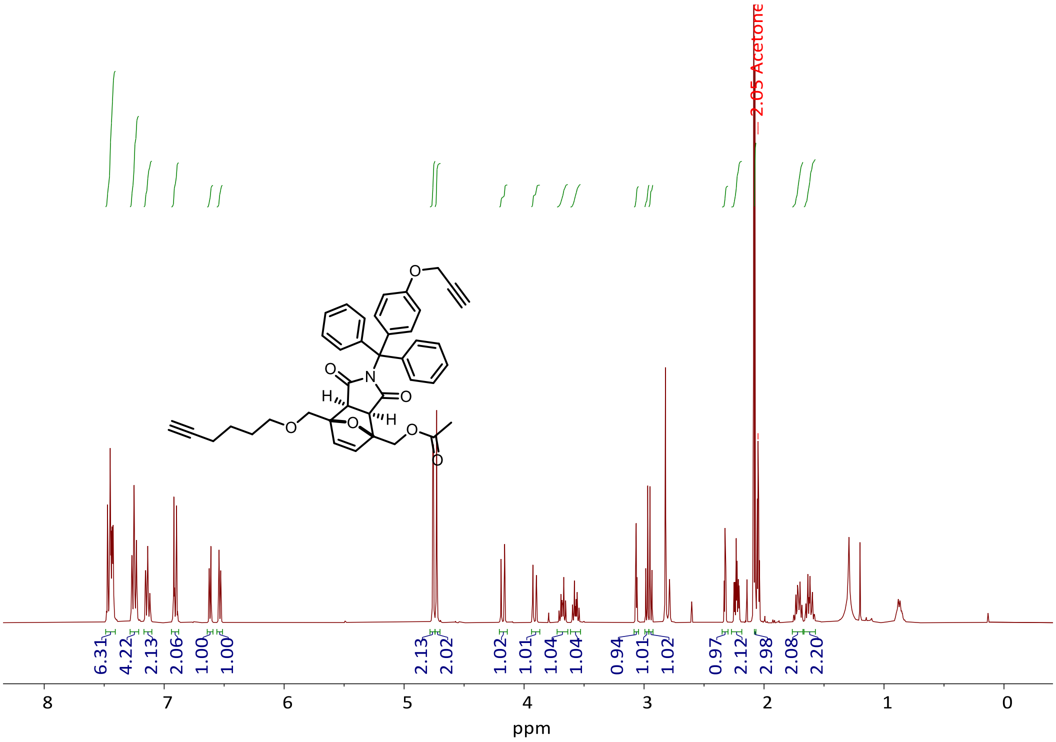

Spectrum S1. ^{1}H NMR (400 MHz, Acetone-d_6, 298 K) spectrum of compound **S4**.

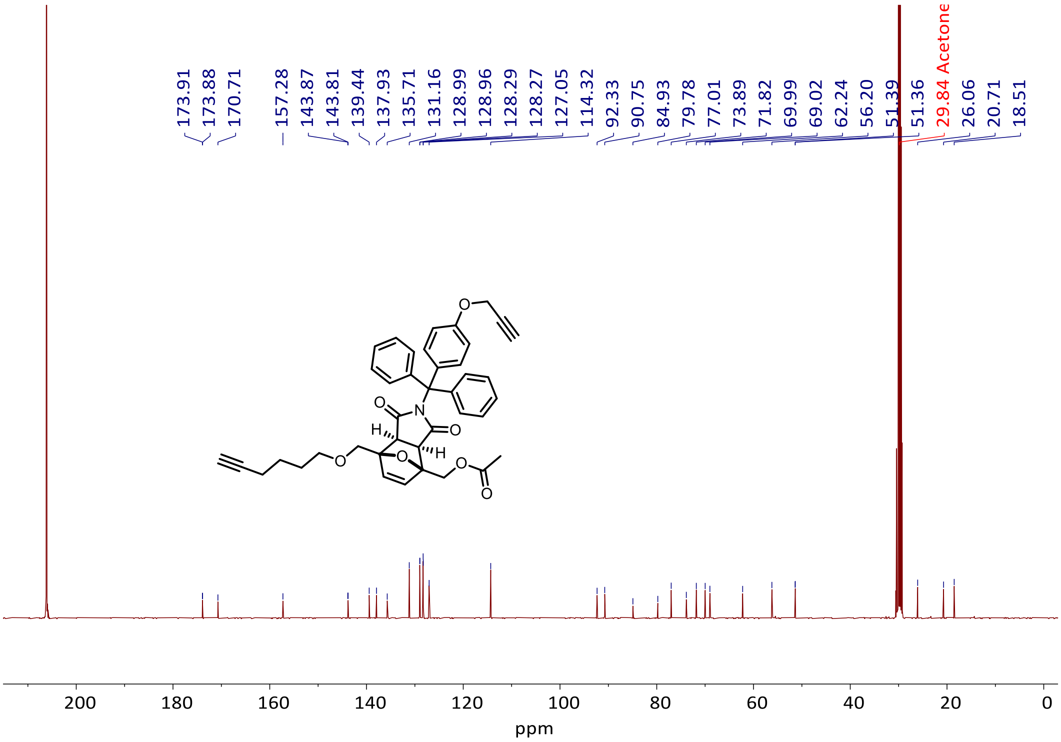

Spectrum S2. ^{13}C NMR (101 MHz, Acetone-d_6, 298 K) spectrum of compound **S4**.

8.1.2 Spectra of S5

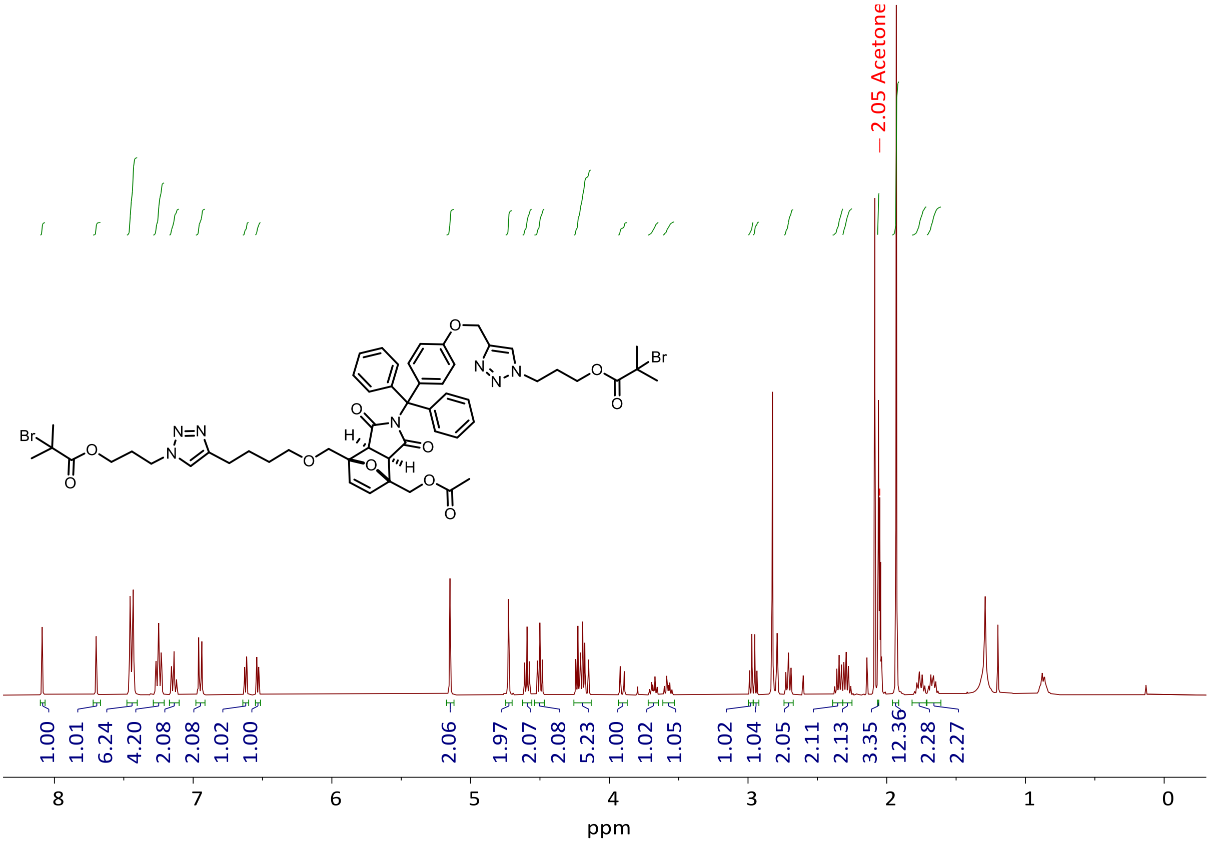

Spectrum S3. ^{1}H NMR (400 MHz, Acetone-d_6, 298 K) spectrum of compound **S5**.

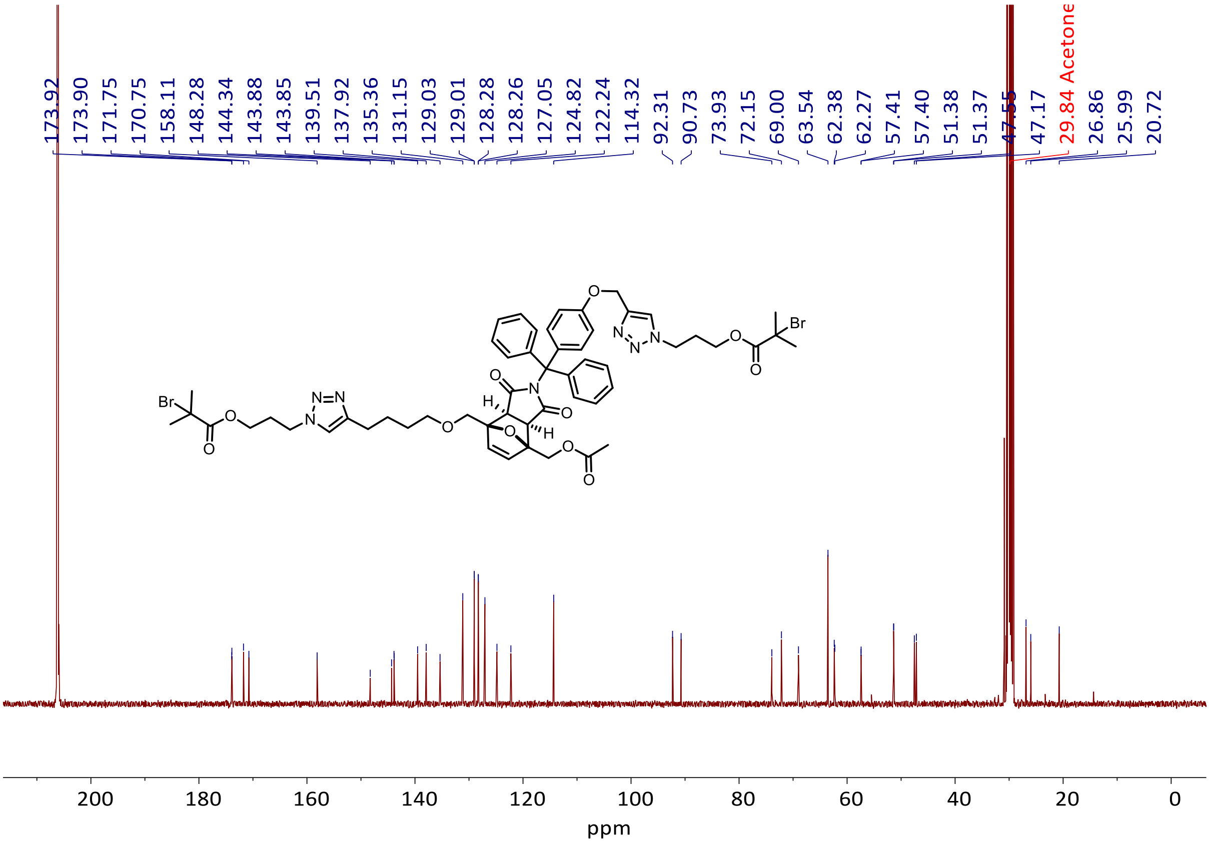

Spectrum S4. ^{13}C NMR (101 MHz, Acetone-d_6, 298 K) spectrum of compound **S5**.

8.1.3 Spectra of S6

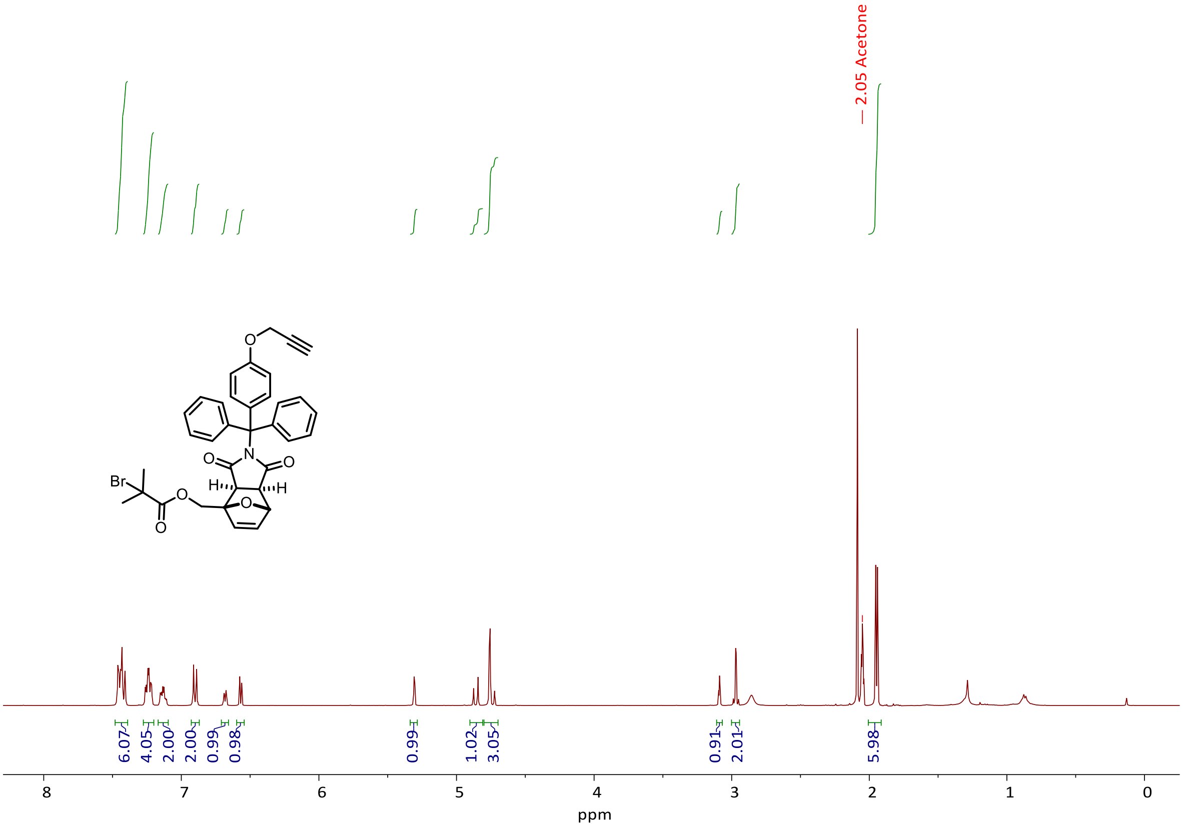

Spectrum S5. ^{1}H NMR (400 MHz, Acetone-d_6, 298 K) spectrum of compound **S6**.

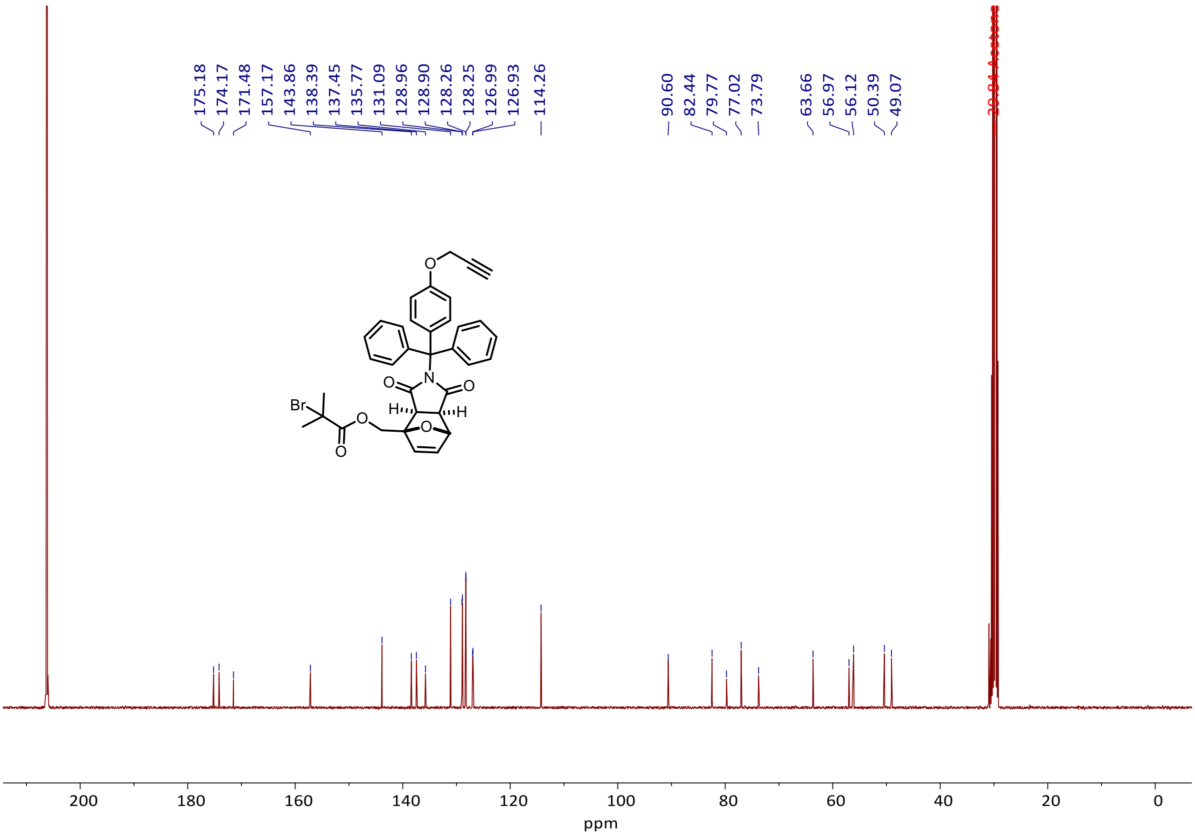

Spectrum S6. ^{13}C NMR (101 MHz, Acetone-d_6, 298 K) spectrum of compound **S6**.

8.1.4 Spectra of S7

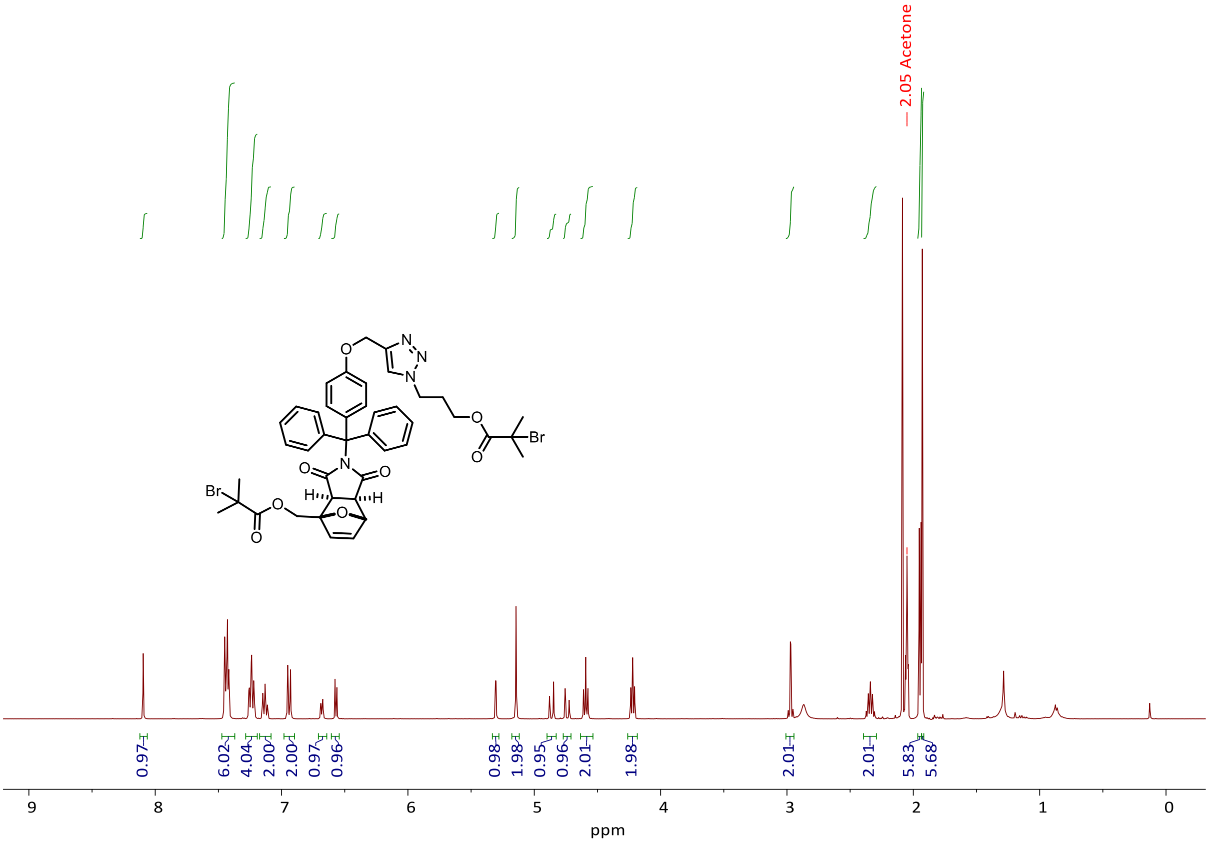

Spectrum S7. ^{1}H NMR (400 MHz, Acetone-d_6, 298 K) spectrum of compound **S7**.

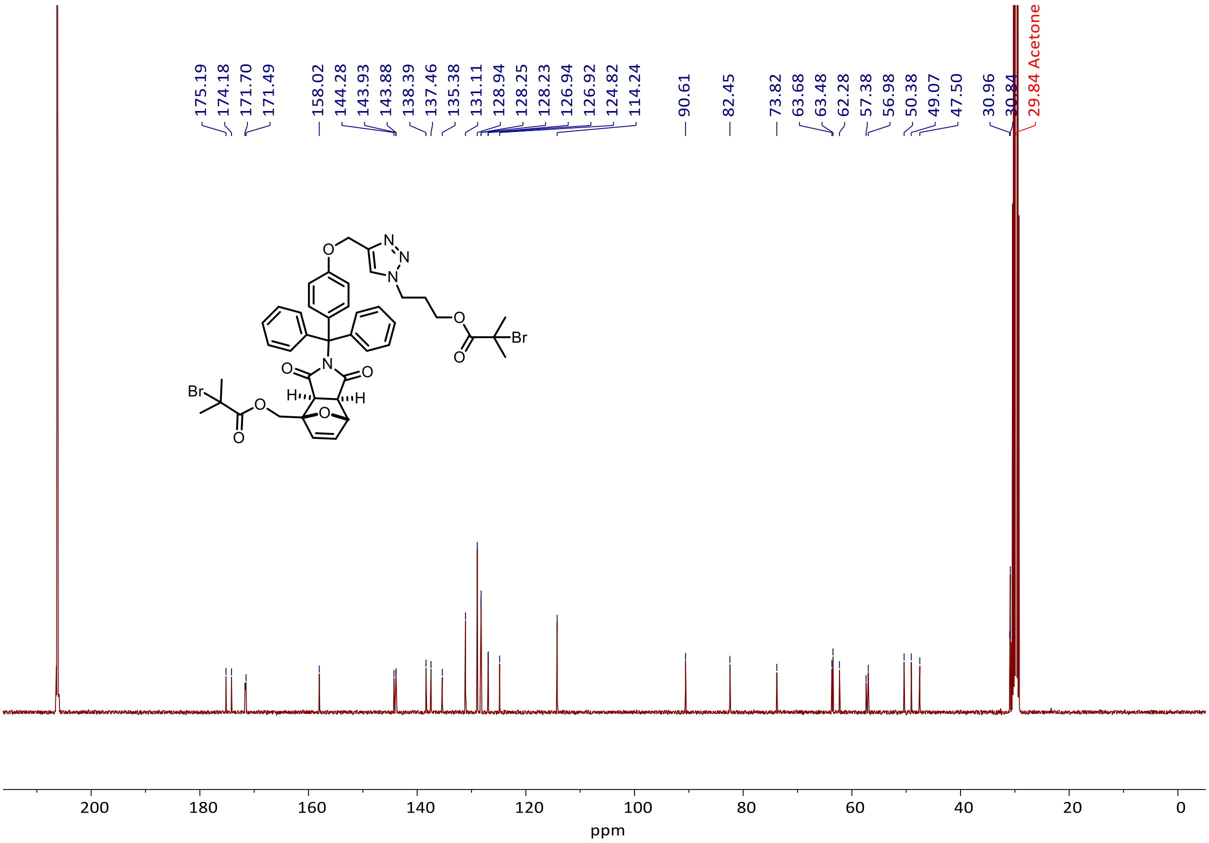

Spectrum S8. ^{13}C NMR (101 MHz, Acetone-d_6, 298 K) spectrum of compound **S7**.

8.1.5 Spectra of S8

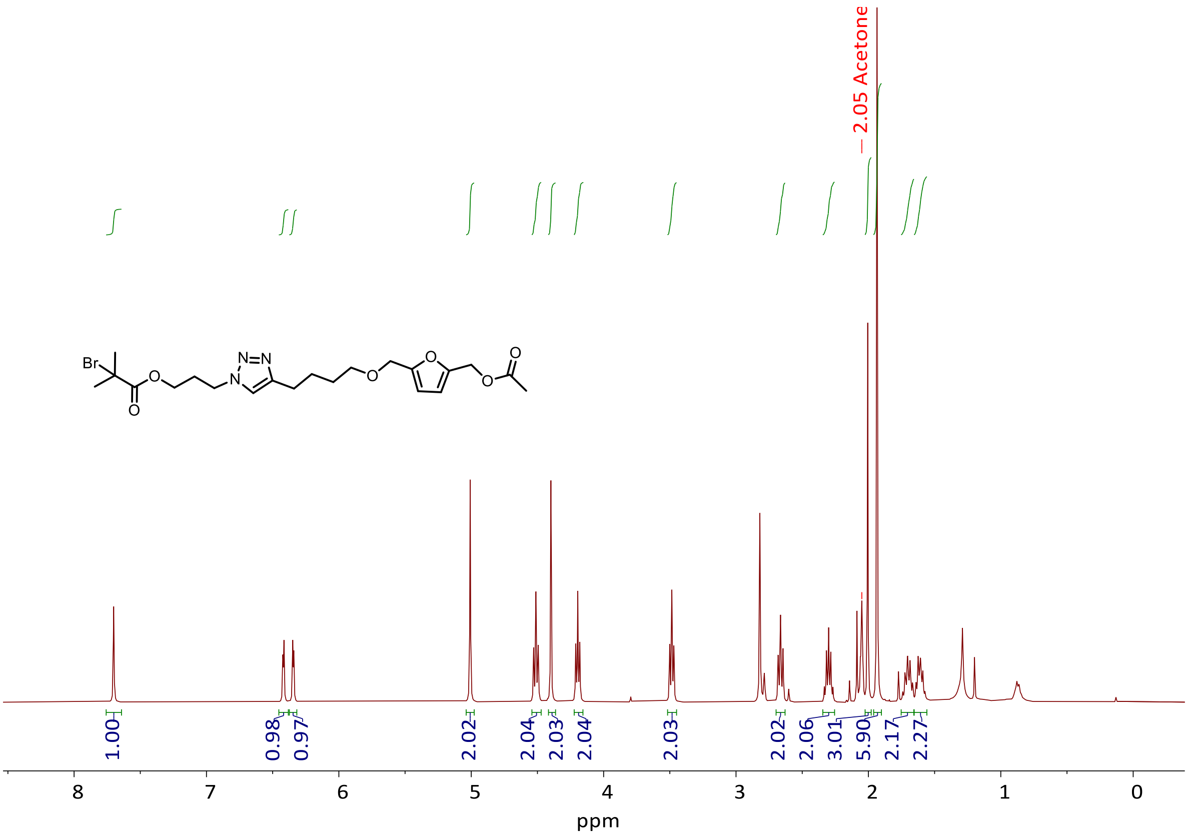

Spectrum S9. ^{1}H NMR (400 MHz, Acetone-d_6, 298 K) spectrum of compound **S8**.

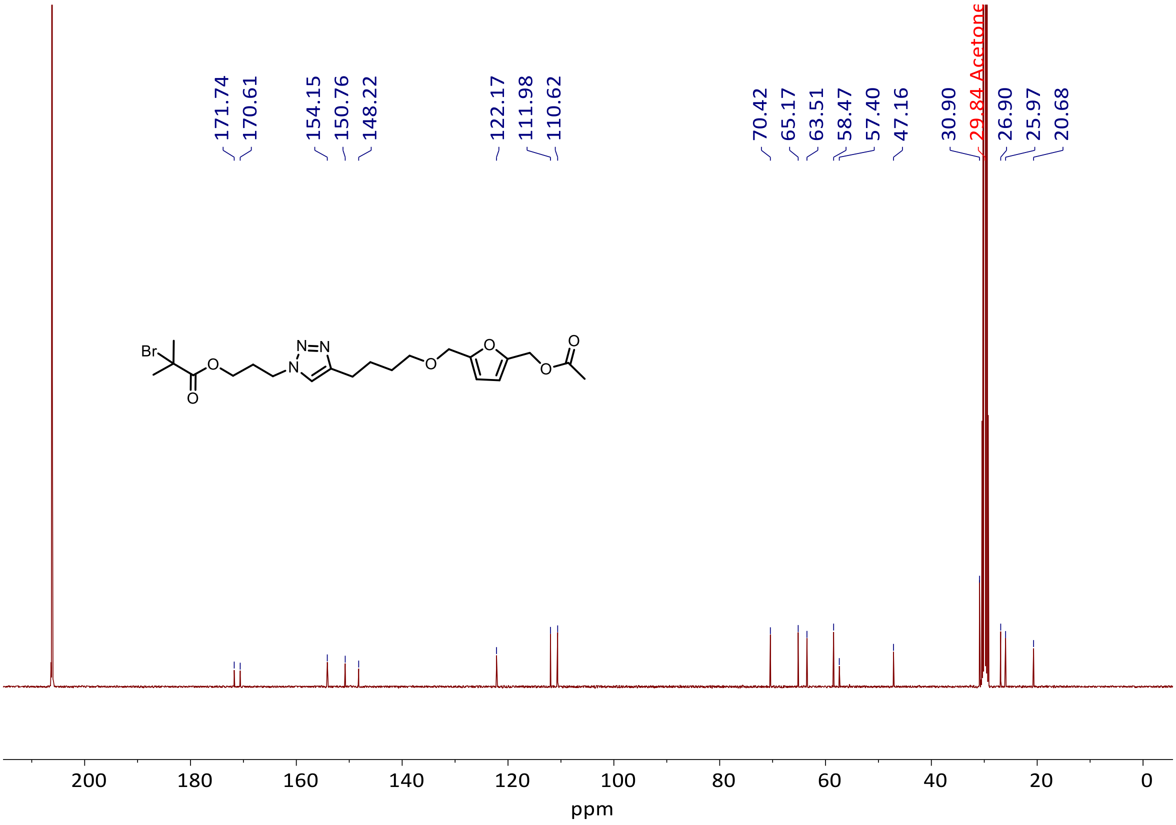

Spectrum S10. ^{13}C NMR (101 MHz, Acetone-d_6, 298 K) spectrum of compound **S8**.

8.1.6 Spectra of S9

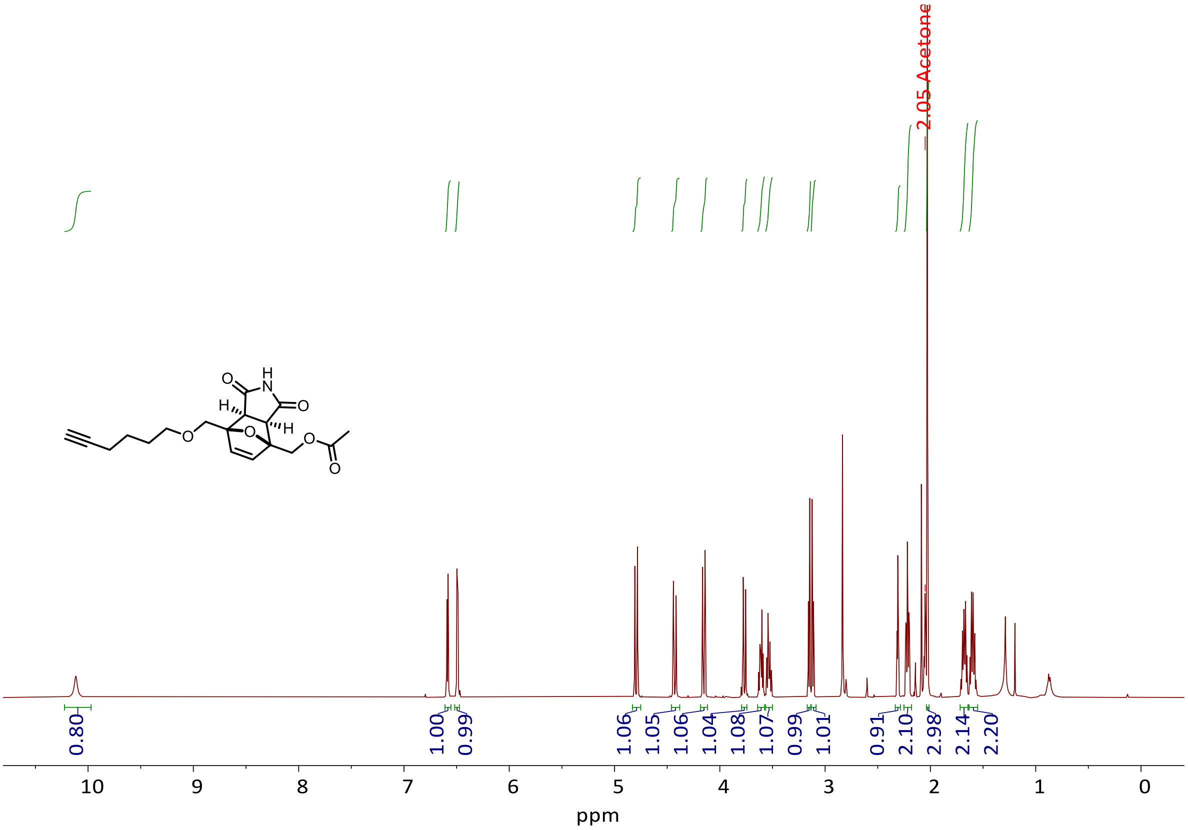

Spectrum S11. ^{1}H NMR (500 MHz, Acetone-d_6, 298 K) spectrum of compound **S9**.

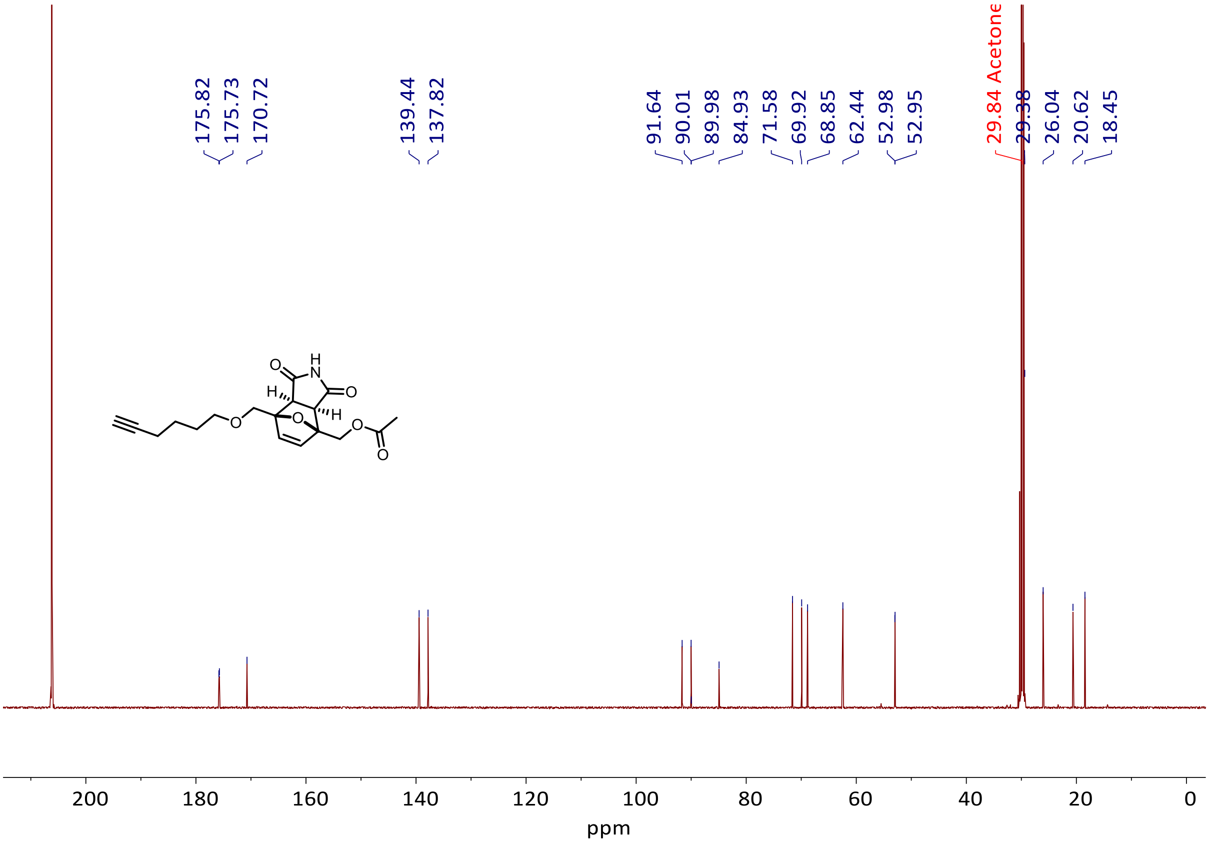

Spectrum S12. ^{13}C NMR (126 MHz, Acetone-d_6, 298 K) spectrum of compound **S9**.

8.1.7 Spectra of S11

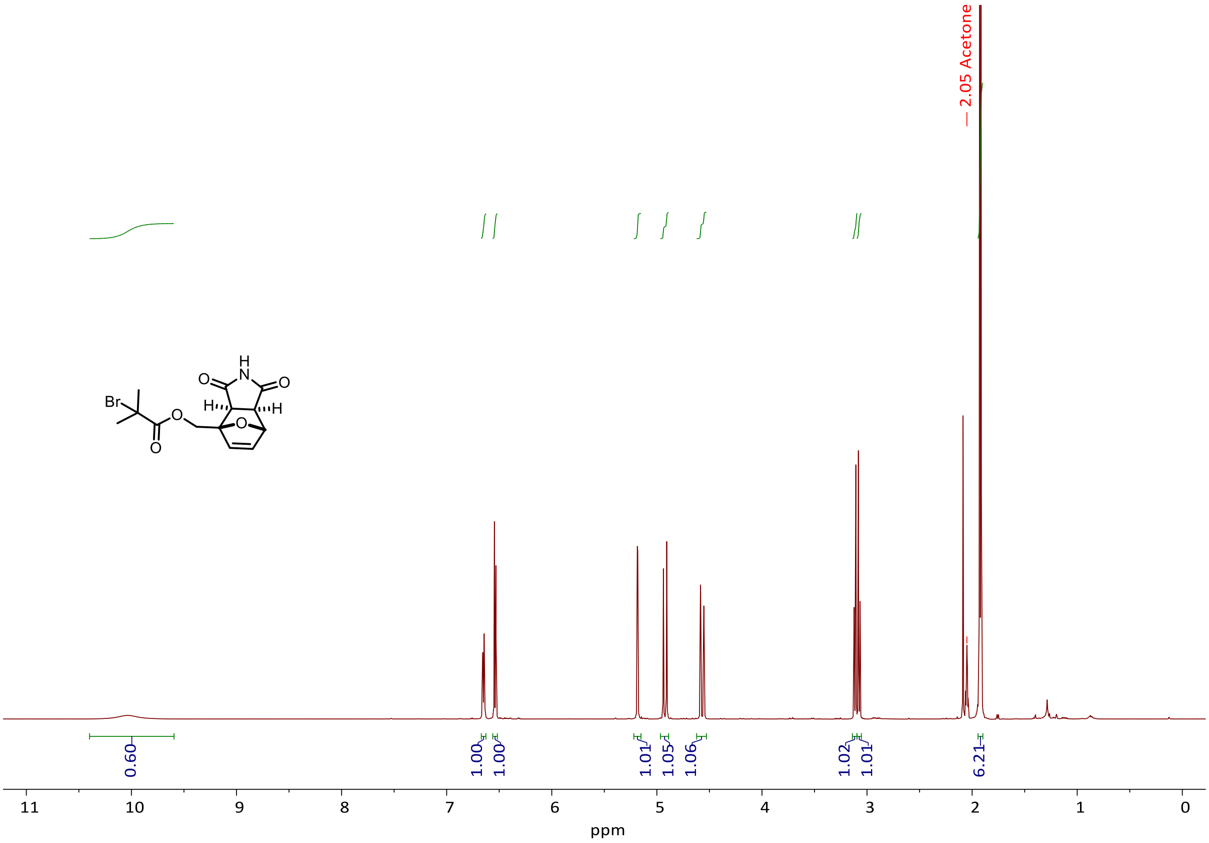

Spectrum S13. ^{1}H NMR (400 MHz, Acetone-d_6, 298 K) spectrum of compound **S11**.

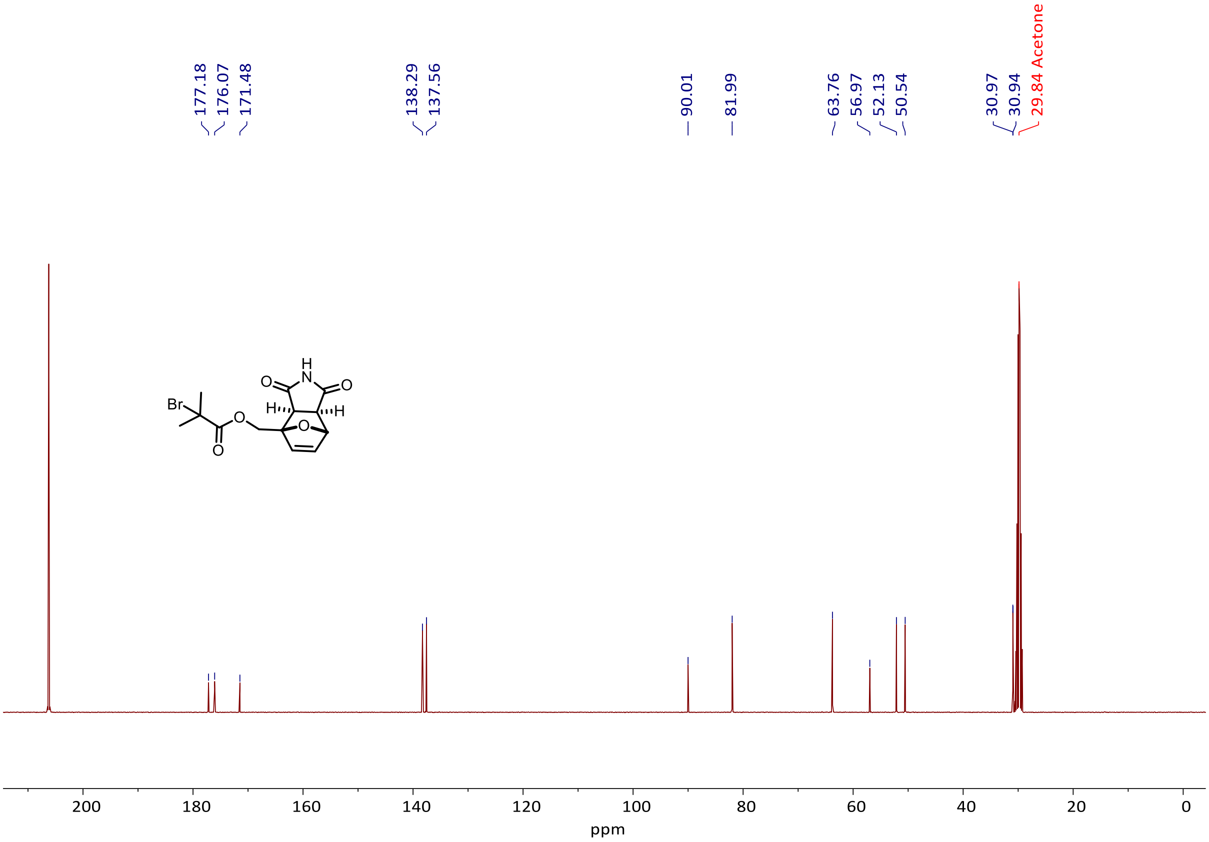

Spectrum S14. ^{13}C NMR (101 MHz, Acetone-d_6, 298 K) spectrum of compound **S11**.

8.1.8 Spectra of S13

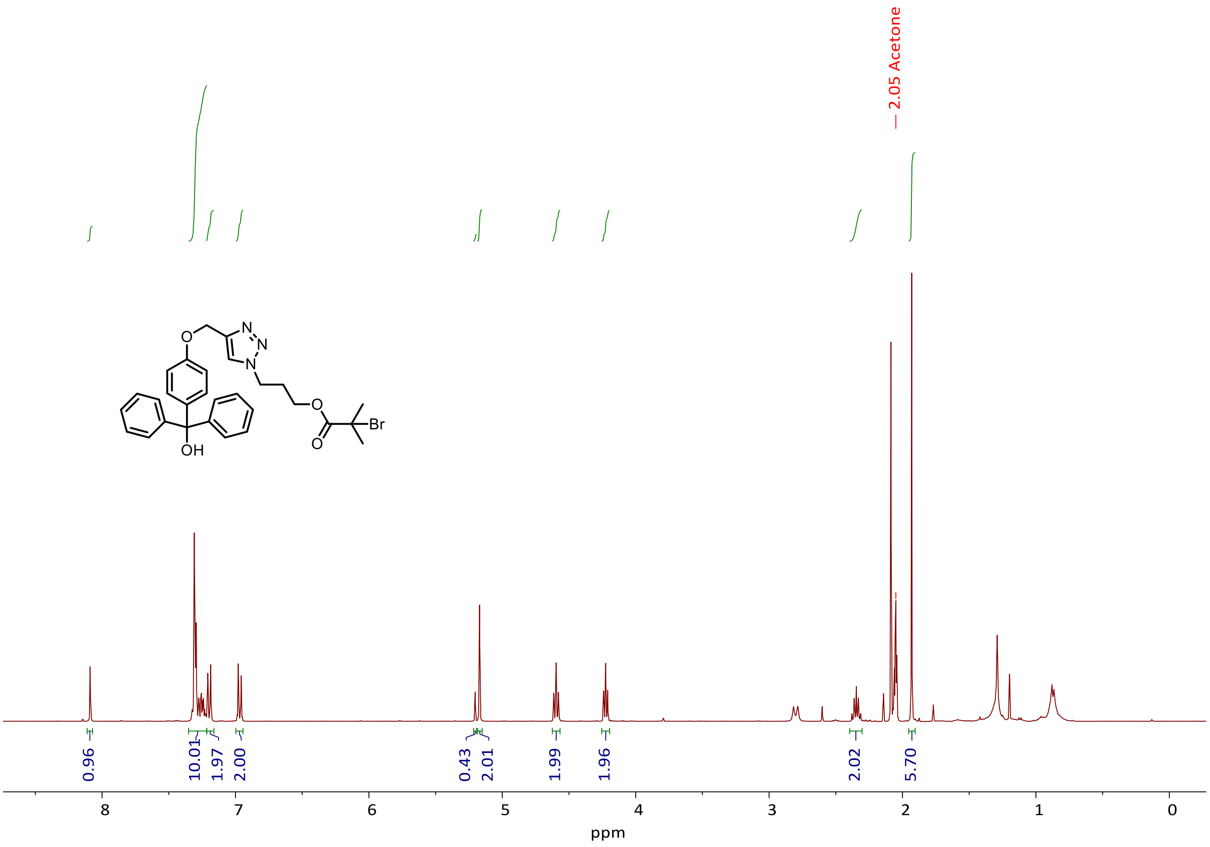

Spectrum S15. ^{1}H NMR (400 MHz, Acetone-d_6, 298 K) spectrum of compound **S13**.

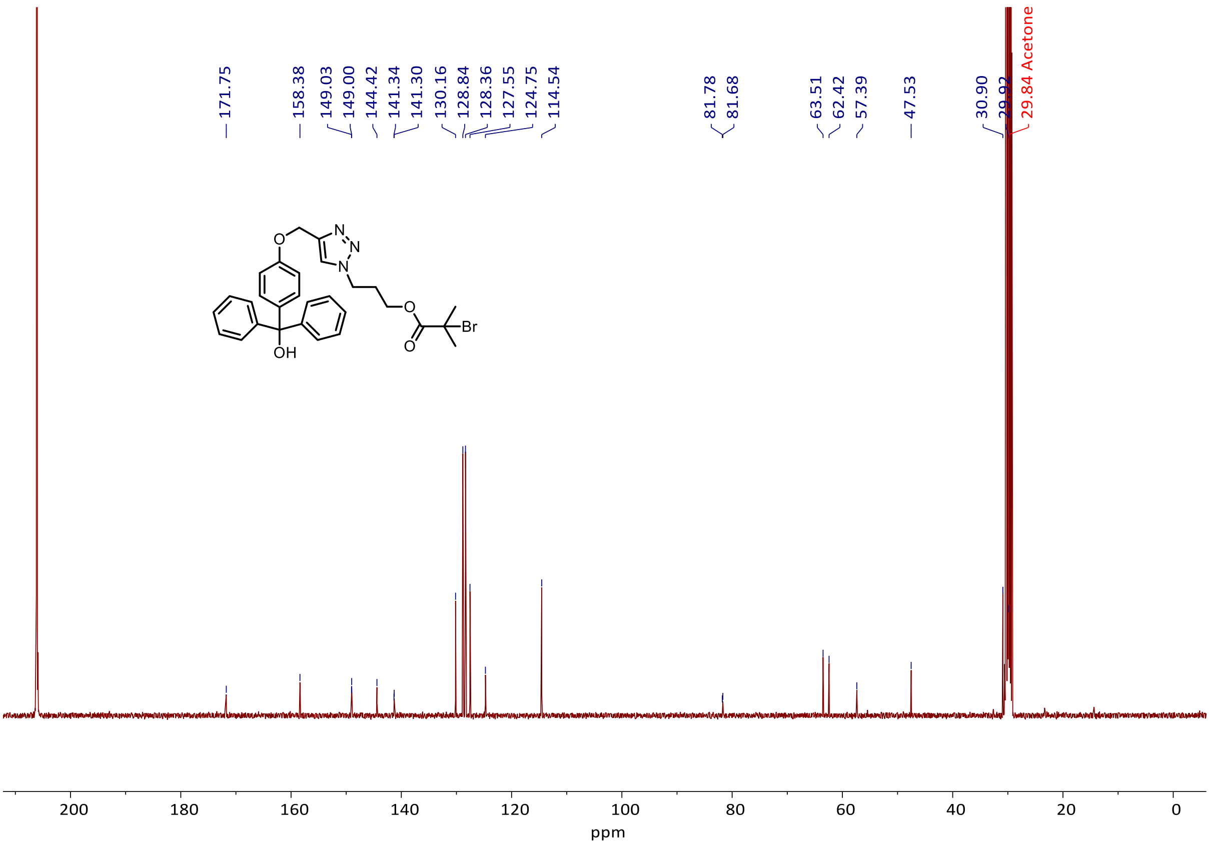

Spectrum S16. ^{13}C NMR (101 MHz, Acetone-d_6, 298 K) spectrum of compound **S13**.

8.1.9 Spectra of S14

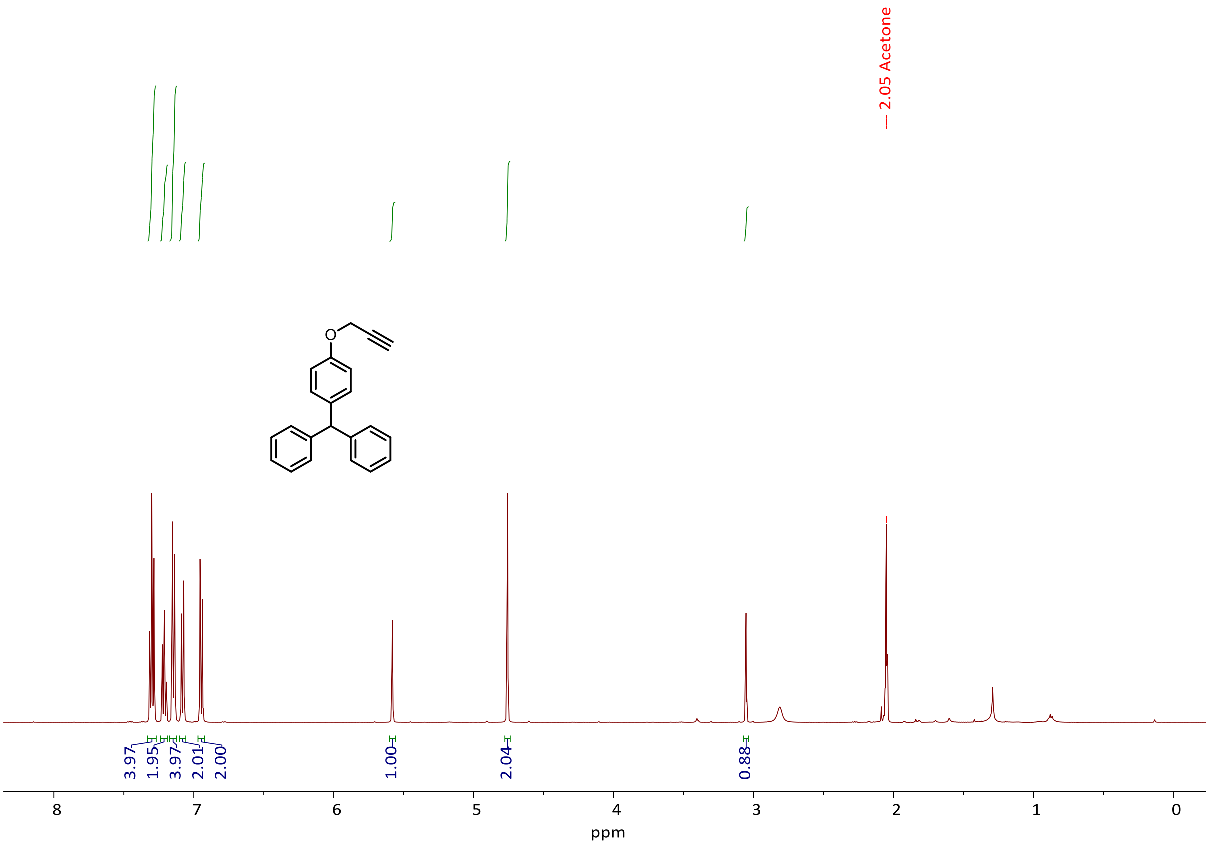

Spectrum S17. ^{1}H NMR (500 MHz, Acetone-d_6, 298 K) spectrum of compound **S14**.

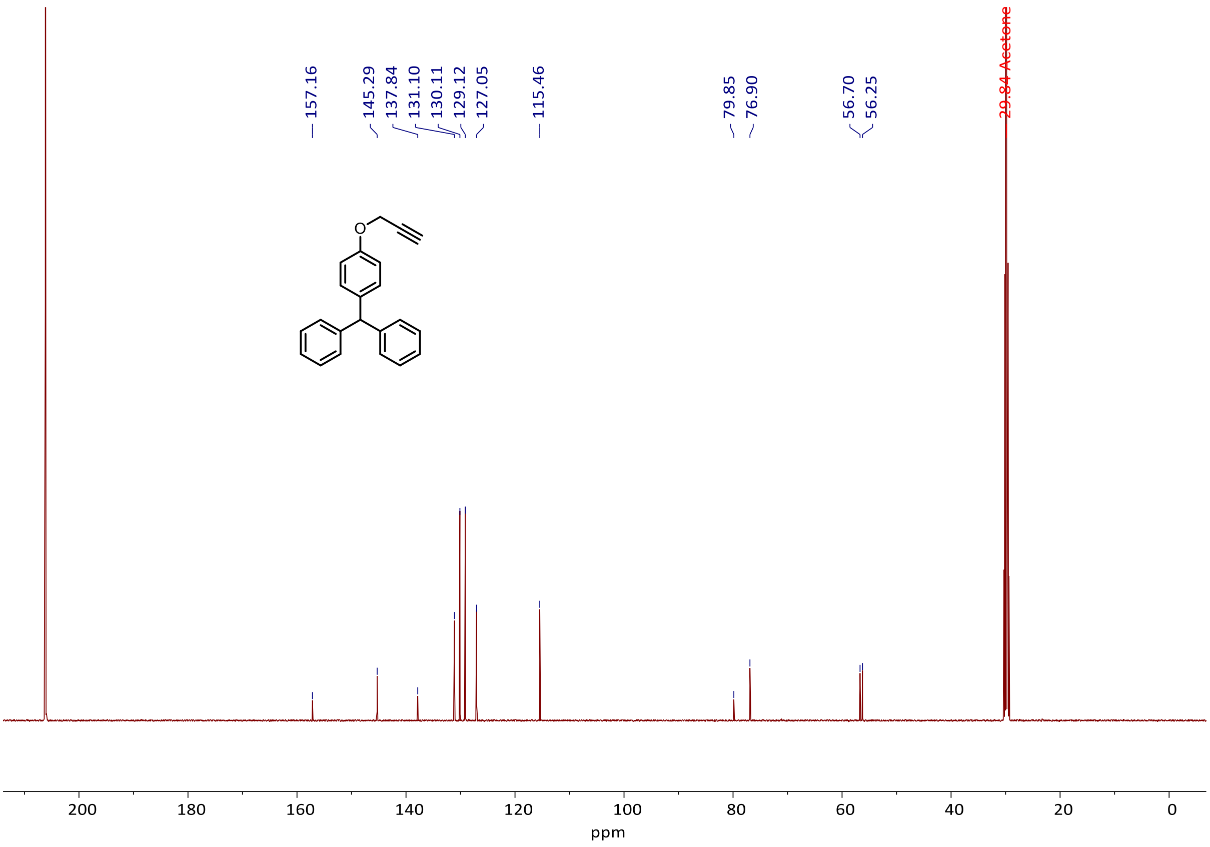

Spectrum S18. ^{13}C NMR (126 MHz, Acetone-d_6, 298 K) spectrum of compound **S14**.

8.1.10 Spectra of S15

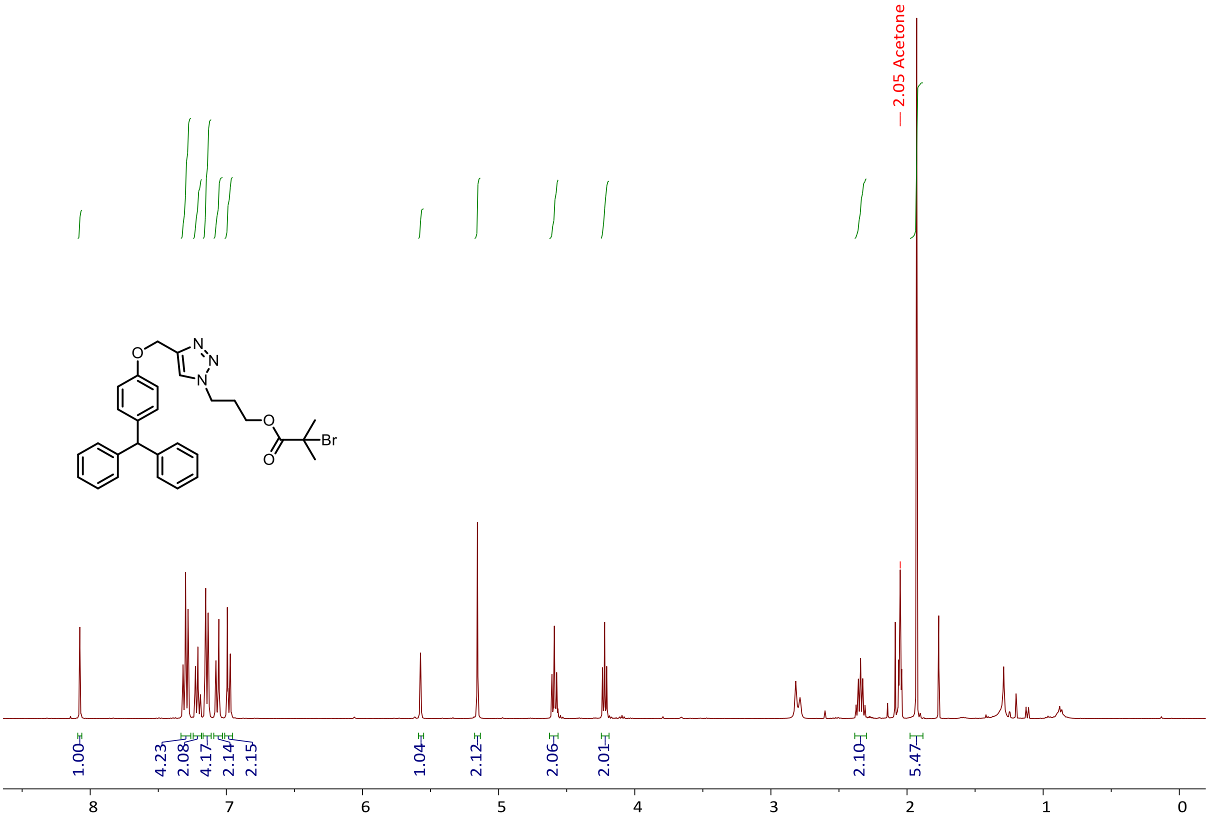

Spectrum S19. ^{1}H NMR (400 MHz, Acetone-d_6, 298 K) spectrum of compound **S15**.

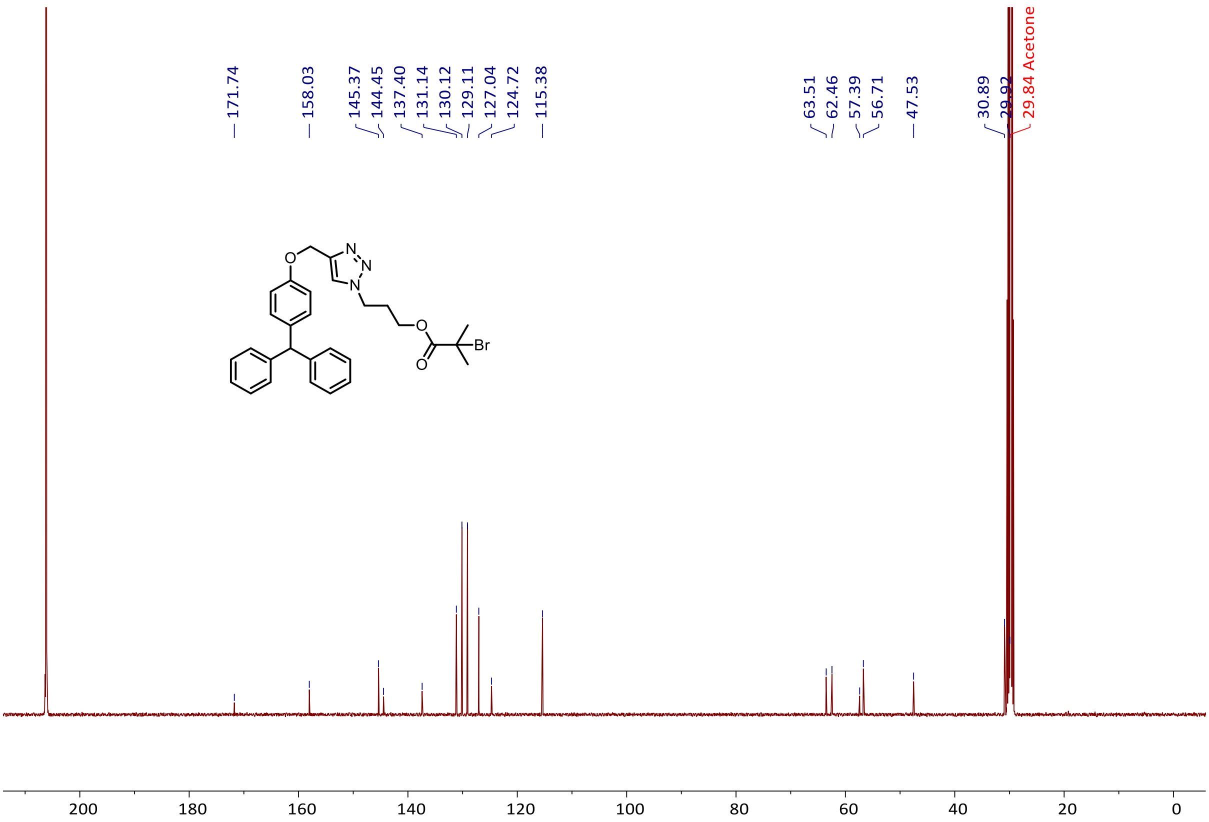

Spectrum S20. ^{13}C NMR (101 MHz, Acetone-d_6, 298 K) spectrum of compound **S15**.

8.2 Polymer NMR Spectra

8.2.1 Spectra of polymer 1

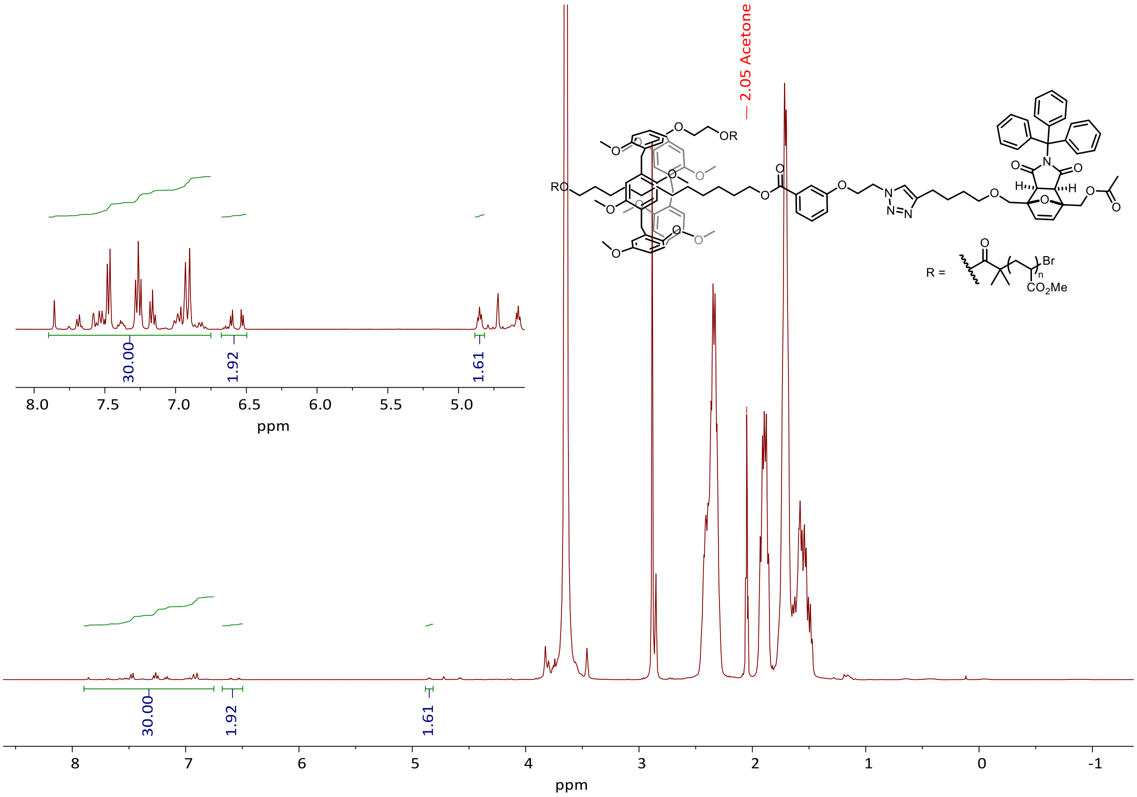

Spectrum S21. ^{1}H NMR (400 MHz, Acetone-d_6, 298 K) spectrum of polymer **1**.

8.2.2 Spectra of polymer 4

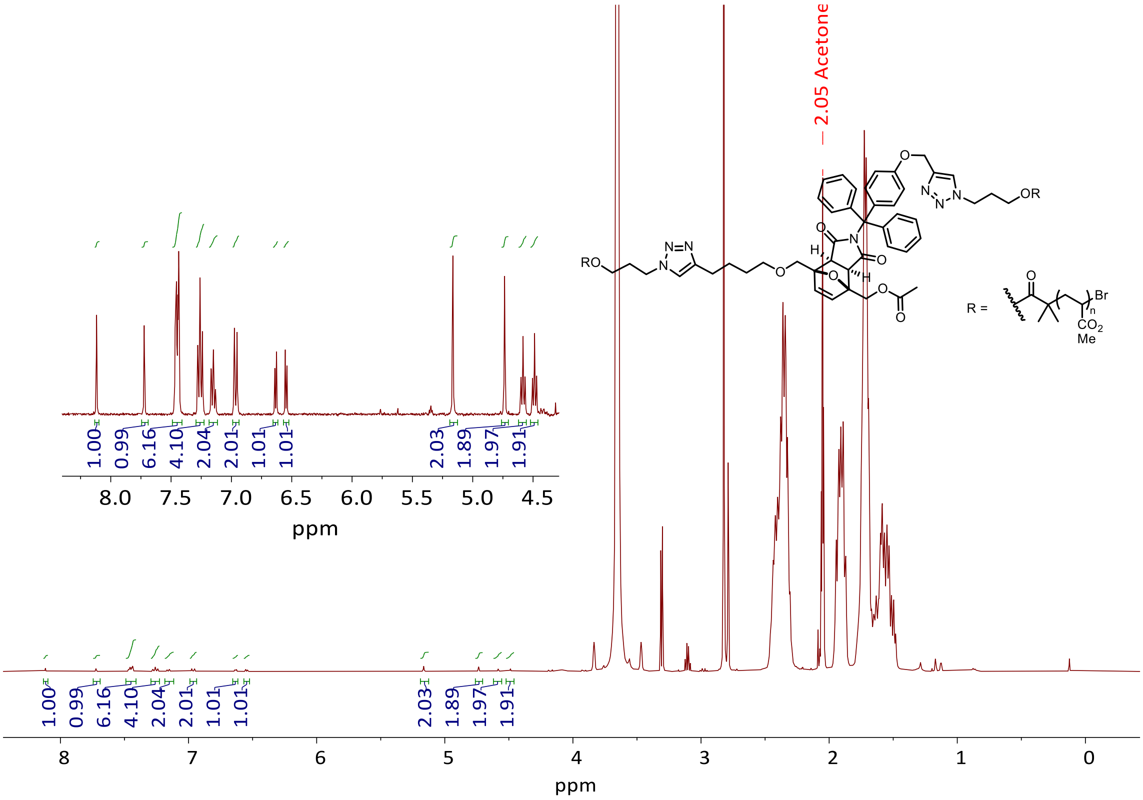

Spectrum S22. ^{1}H NMR (400 MHz, Acetone-d_6, 298 K) spectrum of polymer **4**.

8.2.3 Spectra of polymer S17

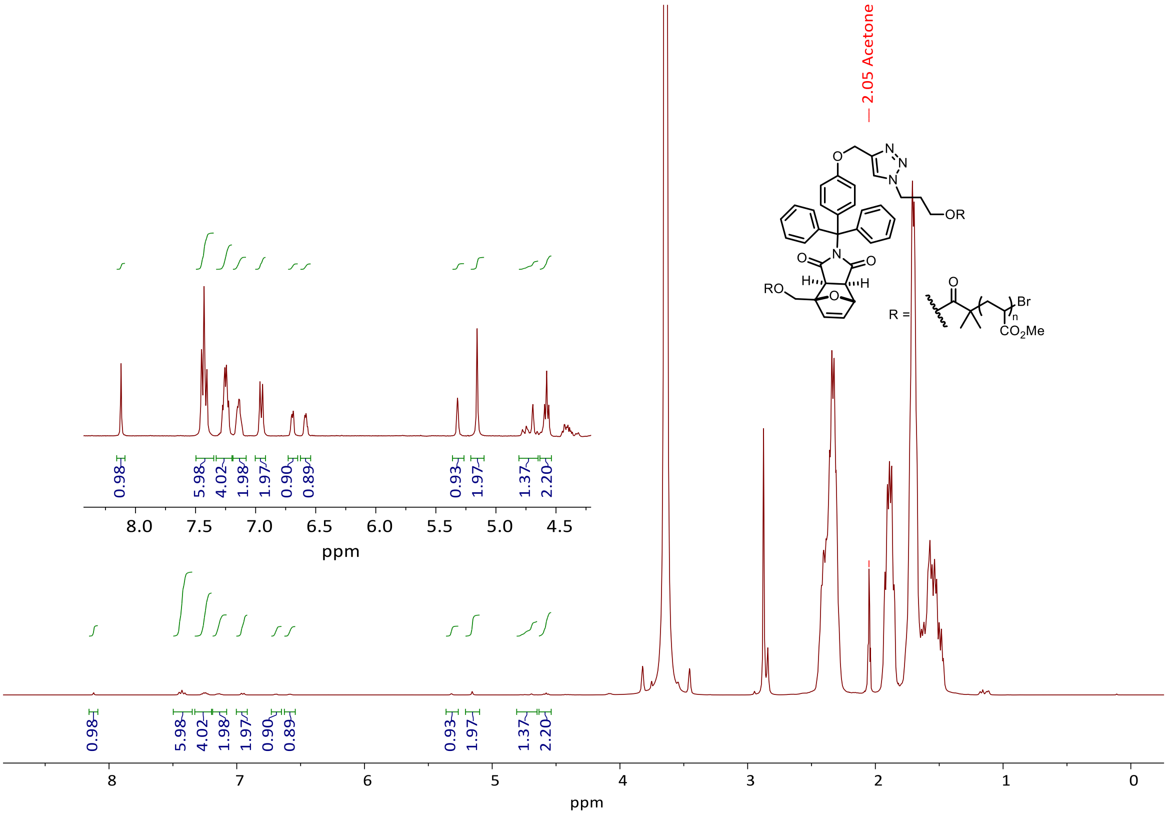

Spectrum S23. ^{1}H NMR (400 MHz, Acetone-d_6, 298 K) spectrum of polymer **S17**.

8.2.4 Spectra of polymer 5

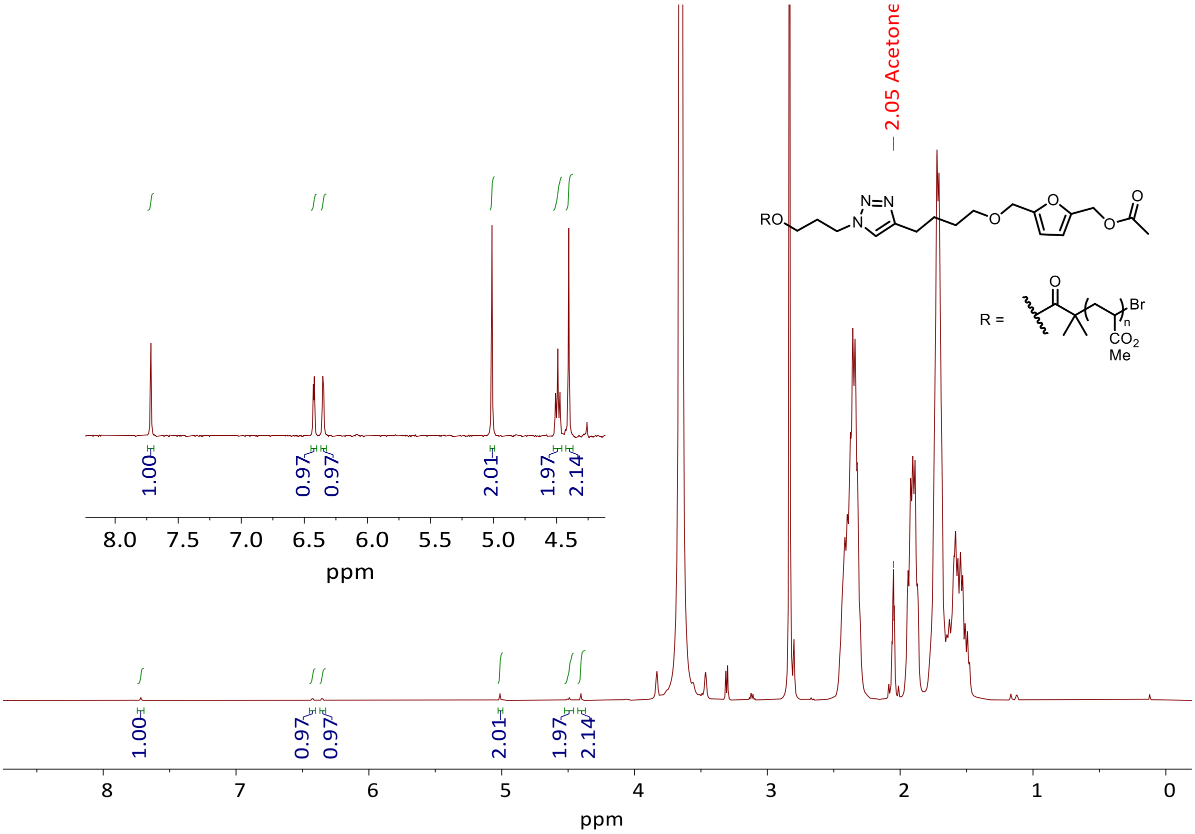

Spectrum S24. ^{1}H NMR (400 MHz, Acetone-d_6, 298 K) spectrum of polymer **5**.

8.2.5 Spectra of polymer S18

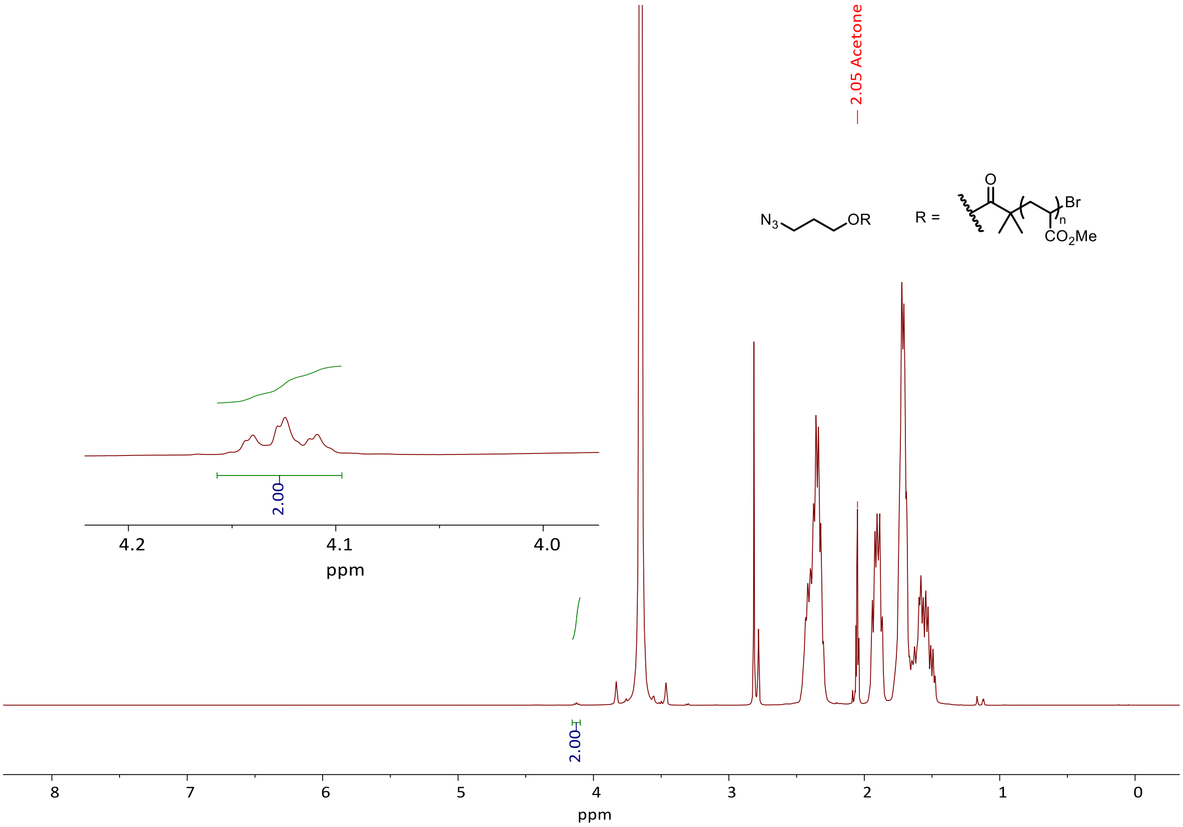

Spectrum S25. ^{1}H NMR (400 MHz, Acetone-d_6, 298 K) spectrum of polymer **S18**

8.2.6 Spectra of polymer 6

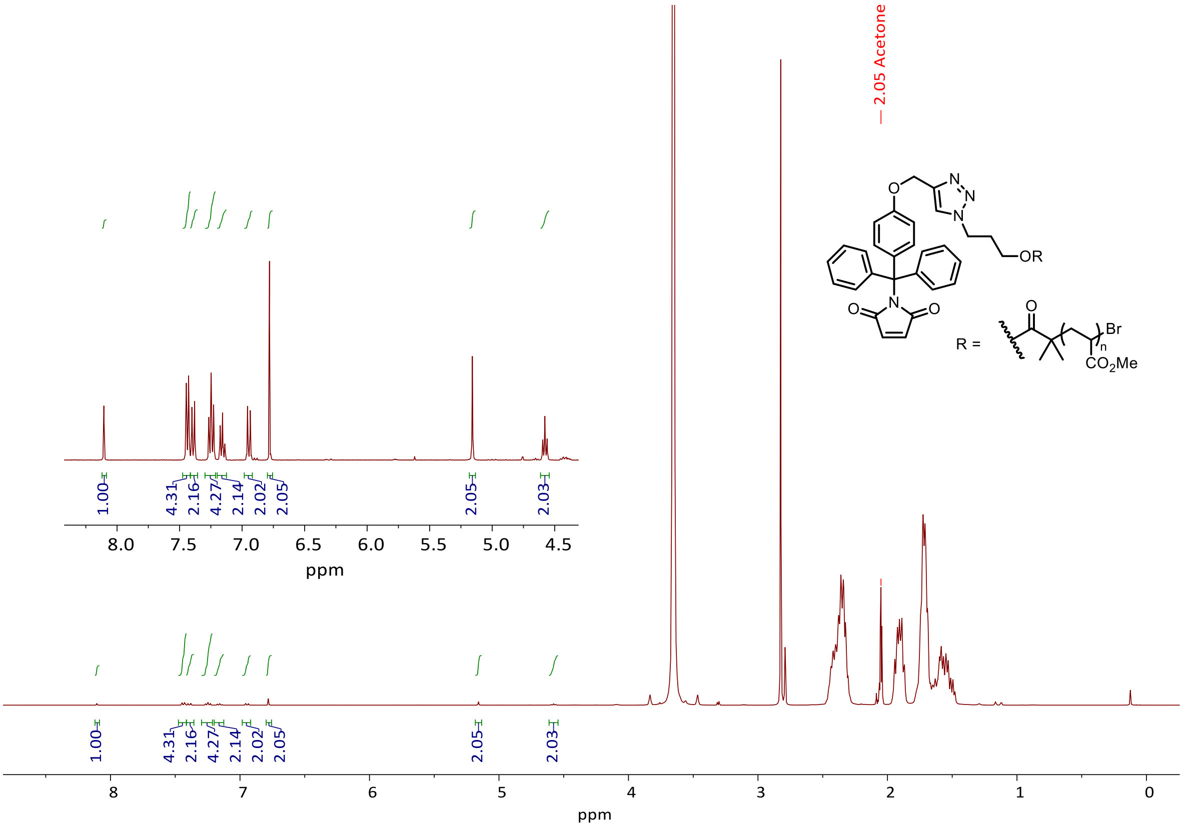

Spectrum S26. ^{1}H NMR (400 MHz, Acetone-d_6, 298 K) spectrum of polymer **6**.

8.2.7 Spectra of polymer 7

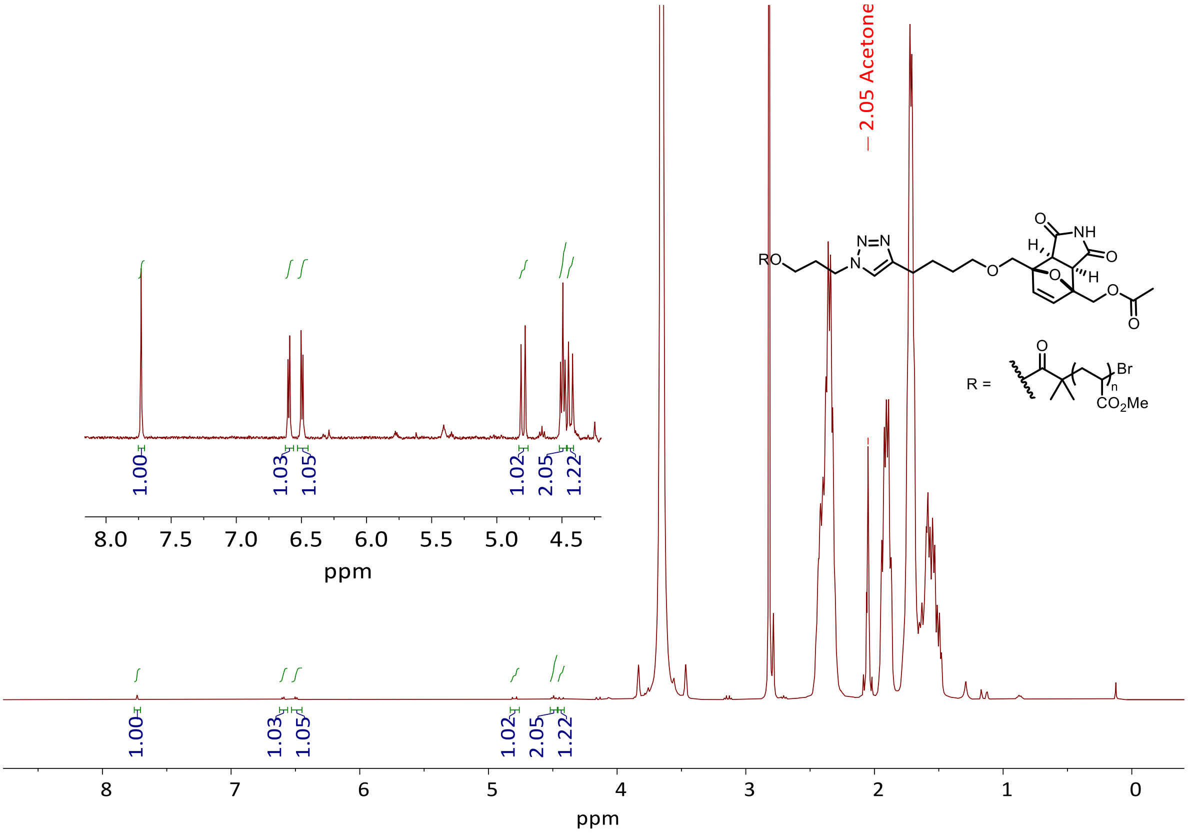

Spectrum S27. ^{1}H NMR (400 MHz, Acetone-d_6, 298 K) spectrum of polymer **7**.

8.2.8 Spectra of polymer 8

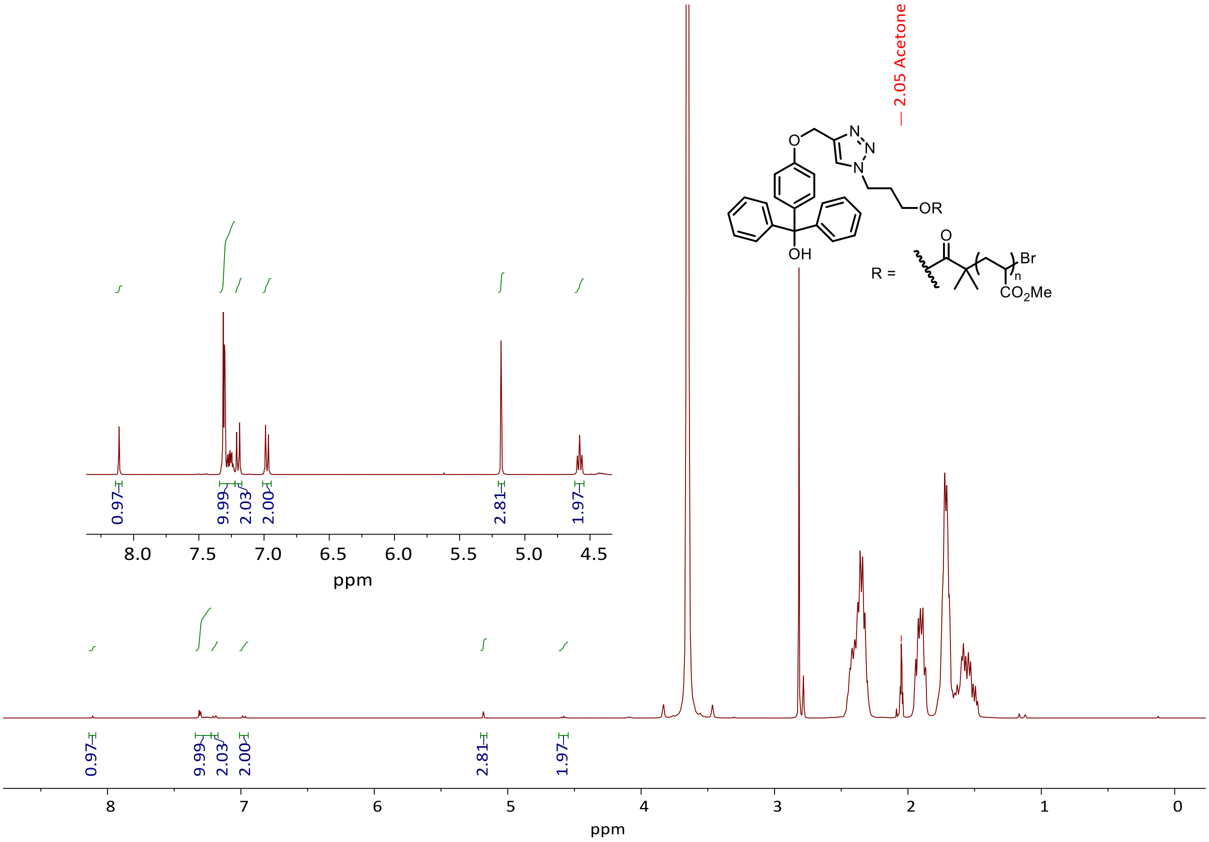

Spectrum S28. ^{1}H NMR (400 MHz, Acetone-d_6, 298 K) spectrum of polymer **8**.

8.2.9 Spectra of polymer S19

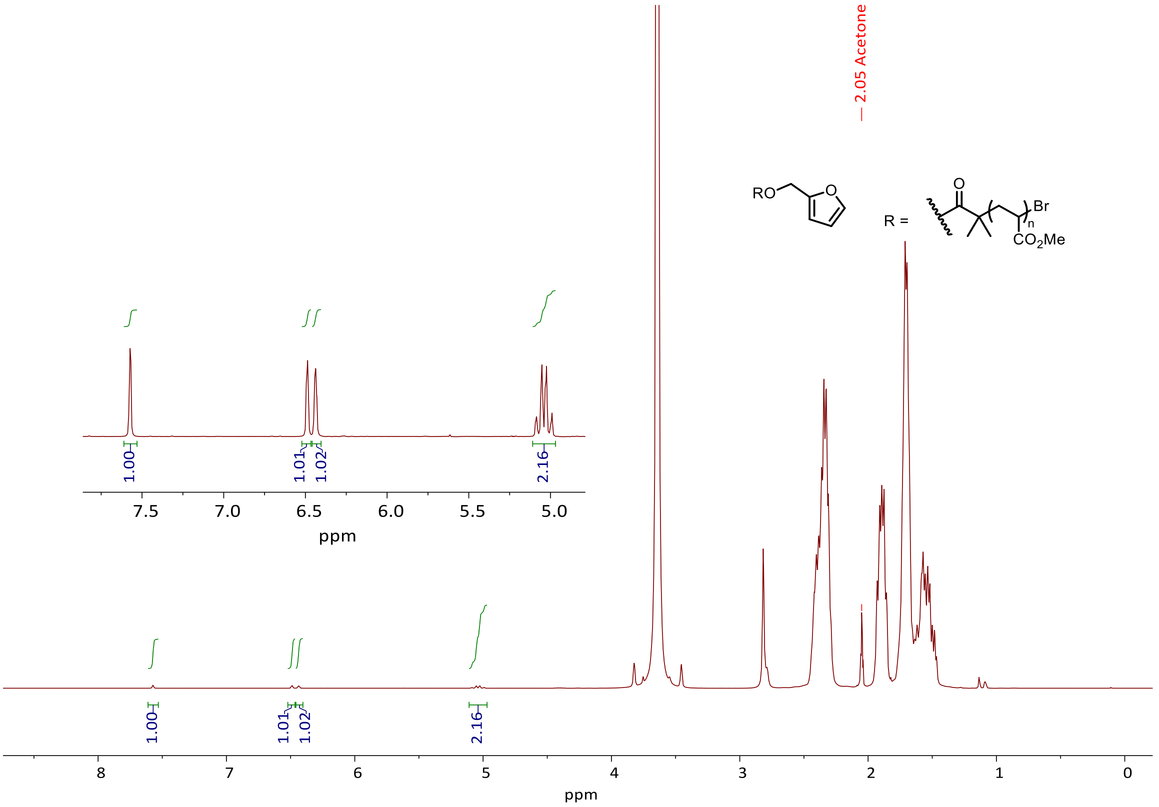

Spectrum S29. ^{1}H NMR (400 MHz, Acetone-d_6, 298 K) spectrum of polymer **S19**.

8.2.10 Spectra of polymer S20

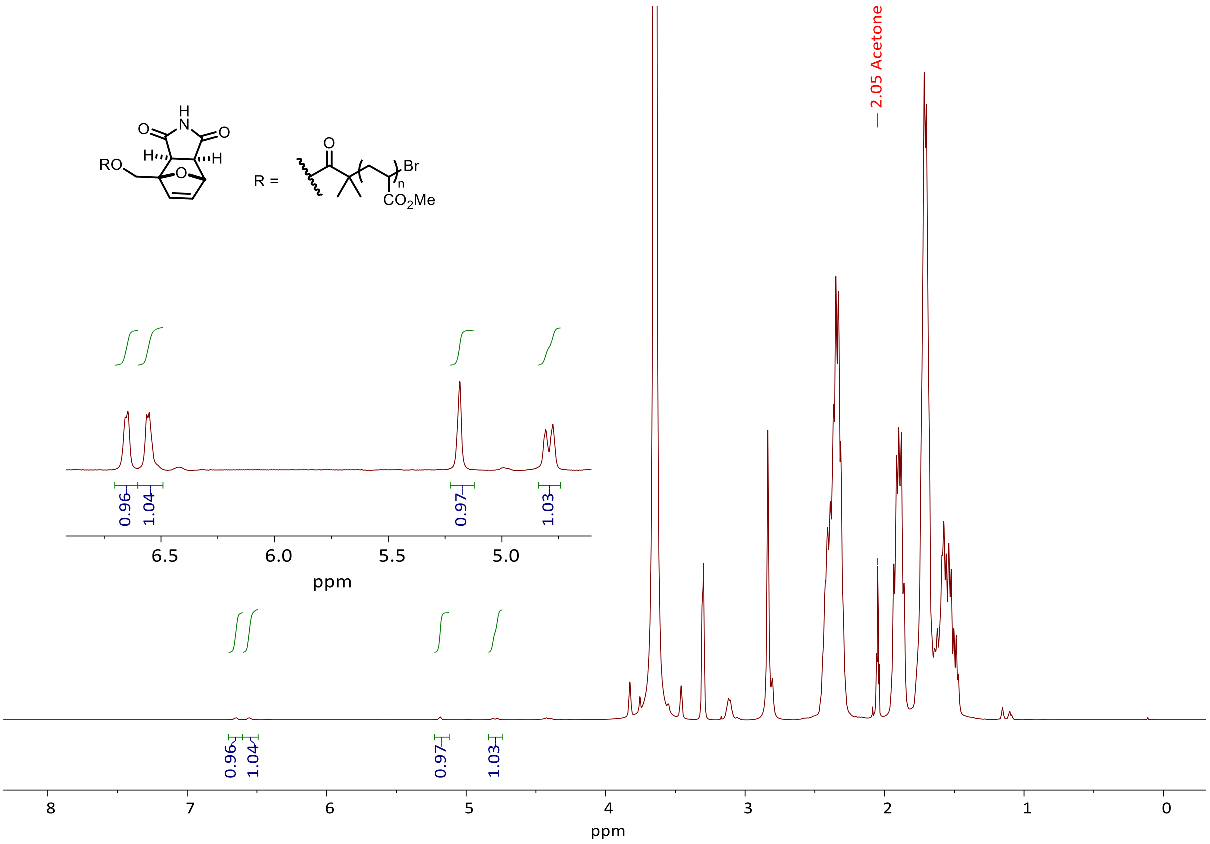

Spectrum S30. ^{1}H NMR (400 MHz, Acetone-d_6, 298 K) spectrum of polymer **S20**.

8.2.11 Spectra of polymer S21

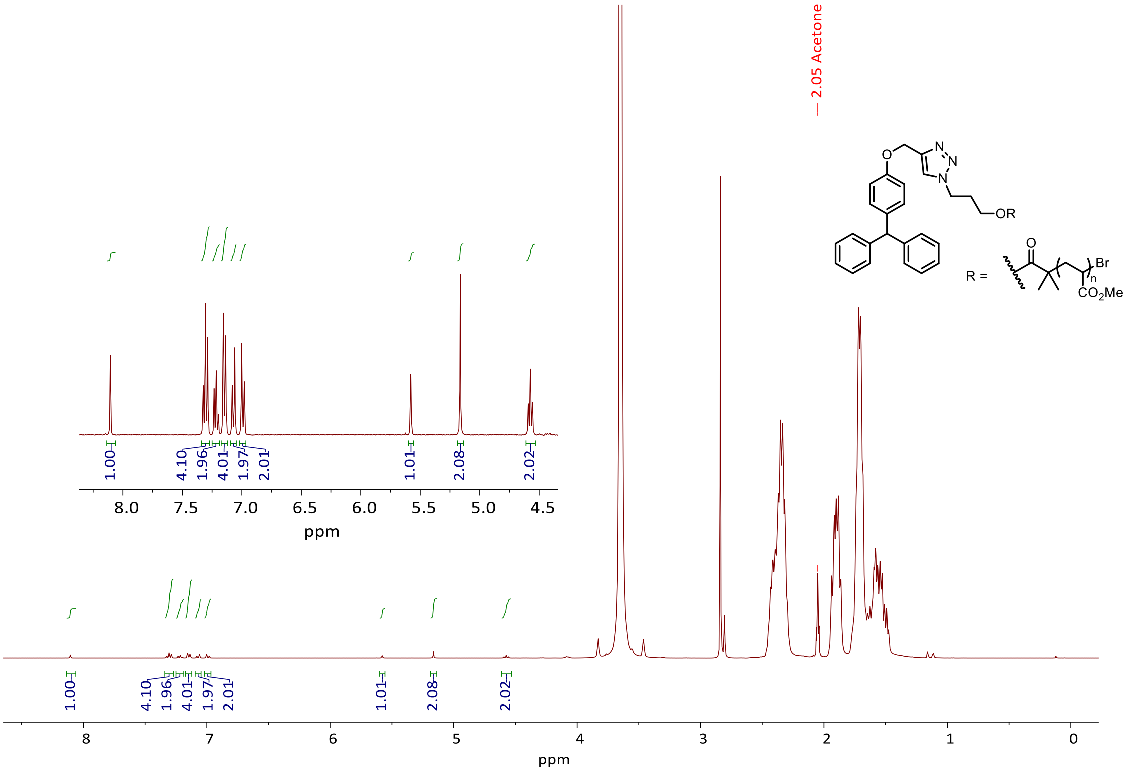

Spectrum S31. ^{1}H NMR (400 MHz, Acetone-d_6, 298 K) spectrum of polymer **S21**.

8.3 Post-Sonication NMR Spectra

8.3.1 Post-Sonication ^{1}H NMR Spectra of Polymer 1

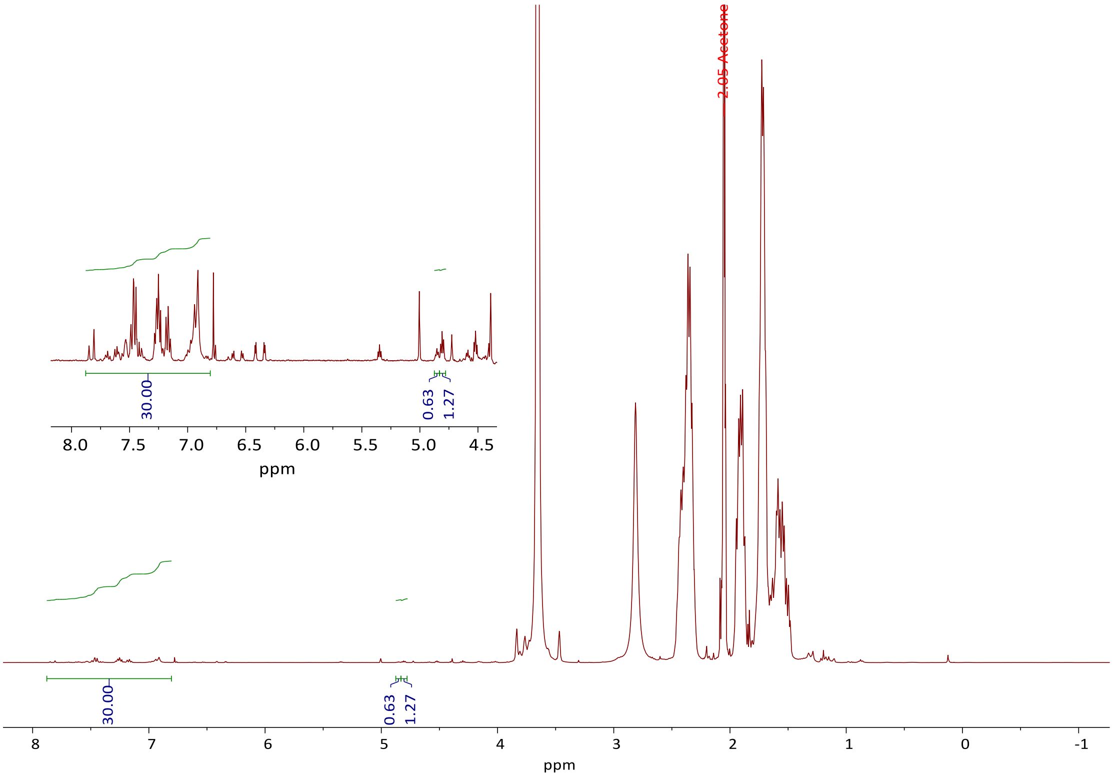

Spectrum S32. ^{1}H NMR (400 MHz, Acetone-d_6, 298 K) spectrum of post-sonication polymer **1** before being washed with methanol.

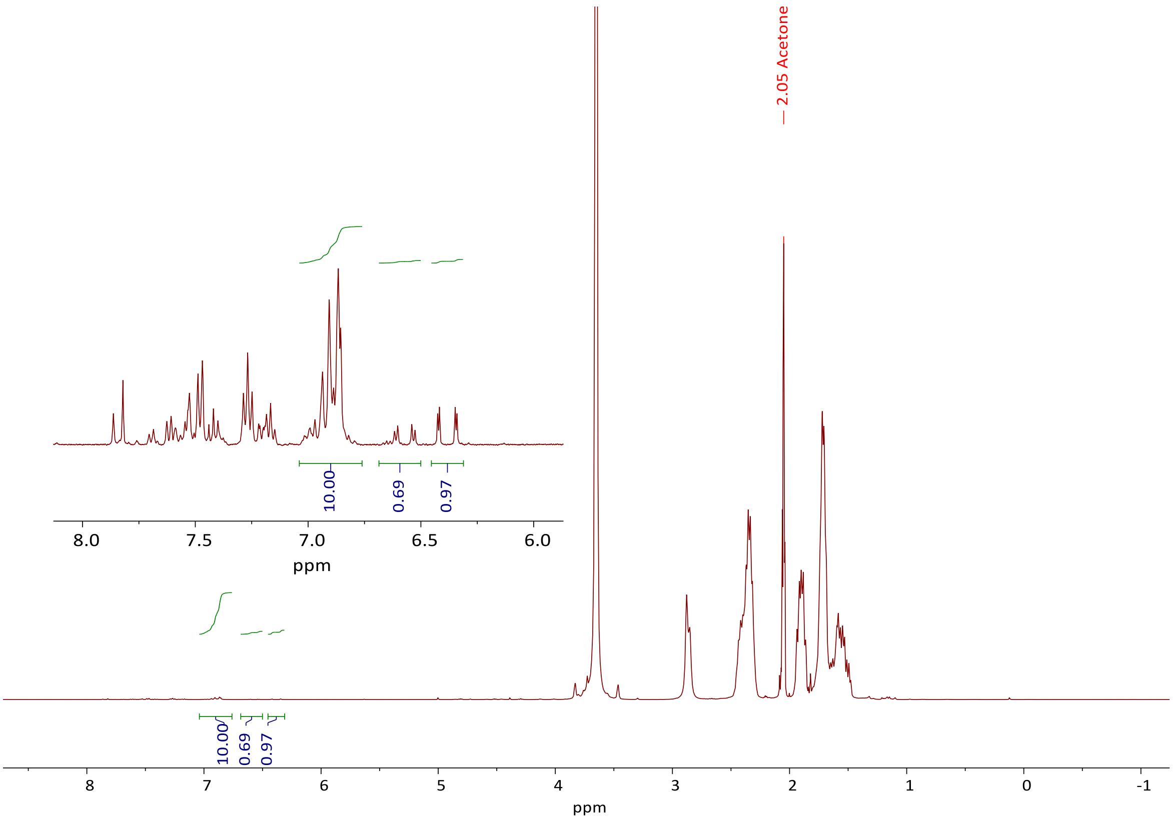

Spectrum S33. ^{1}H NMR (400 MHz, Acetone-d_6, 298 K) spectrum of post-sonication polymer **1** after being washed with methanol.

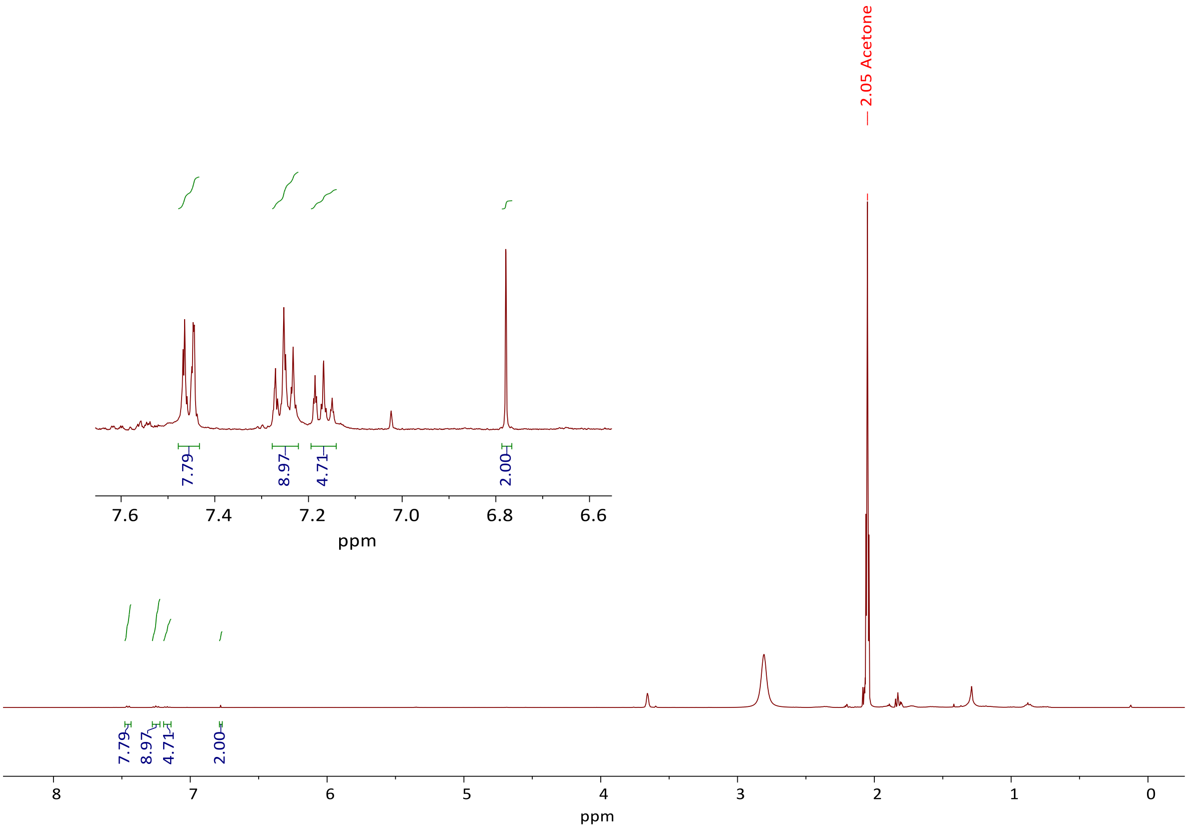

Spectrum S34. ^{1}H NMR (400 MHz, Acetone-d_6, 298 K) spectrum of the concentrated methanol washings from post-sonication polymer **1**.

8.3.2 Post-Sonication ^{1}H NMR Spectra of Polymer 4

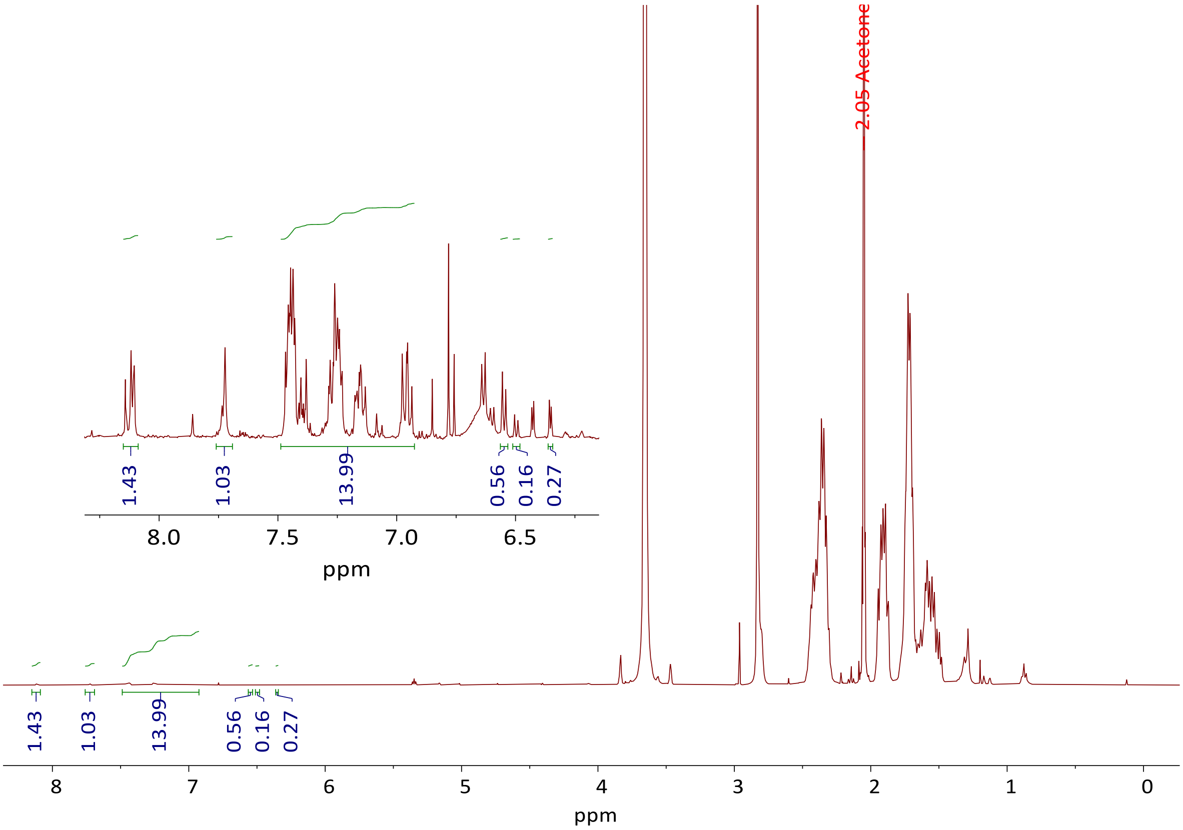

Spectrum S35. ^{1}H NMR (400 MHz, Acetone-d_6, 298 K) spectrum of post-sonication polymer **4** before being washed with methanol.

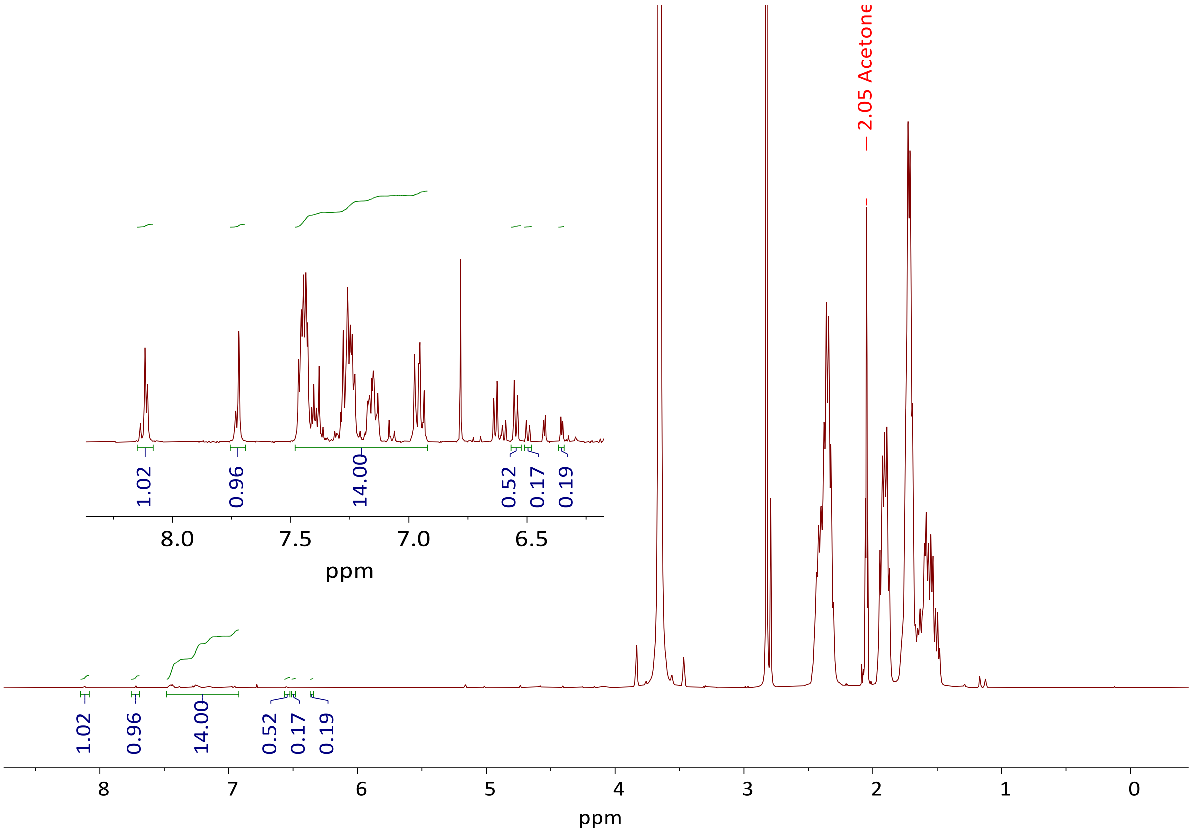

Spectrum S36. ^{1}H NMR (400 MHz, Acetone-d_6, 298 K) spectrum of post-sonication polymer **4** after being washed with methanol.

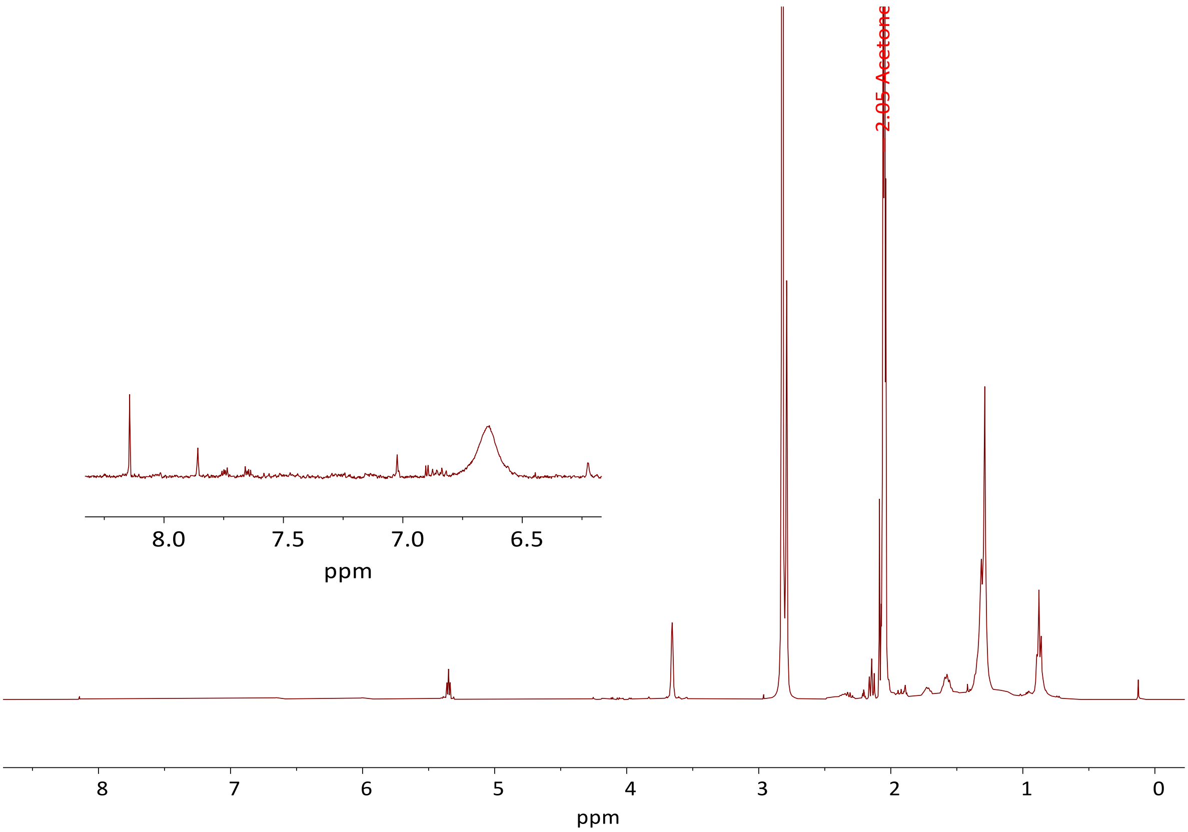

Spectrum S37. ^{1}H NMR (400 MHz, Acetone-d_6, 298 K) spectrum of the concentrated methanol washings from post-sonication polymer **4**.

8.3.1 Post-Sonication ^{1}H NMR Spectra of Polymer S17

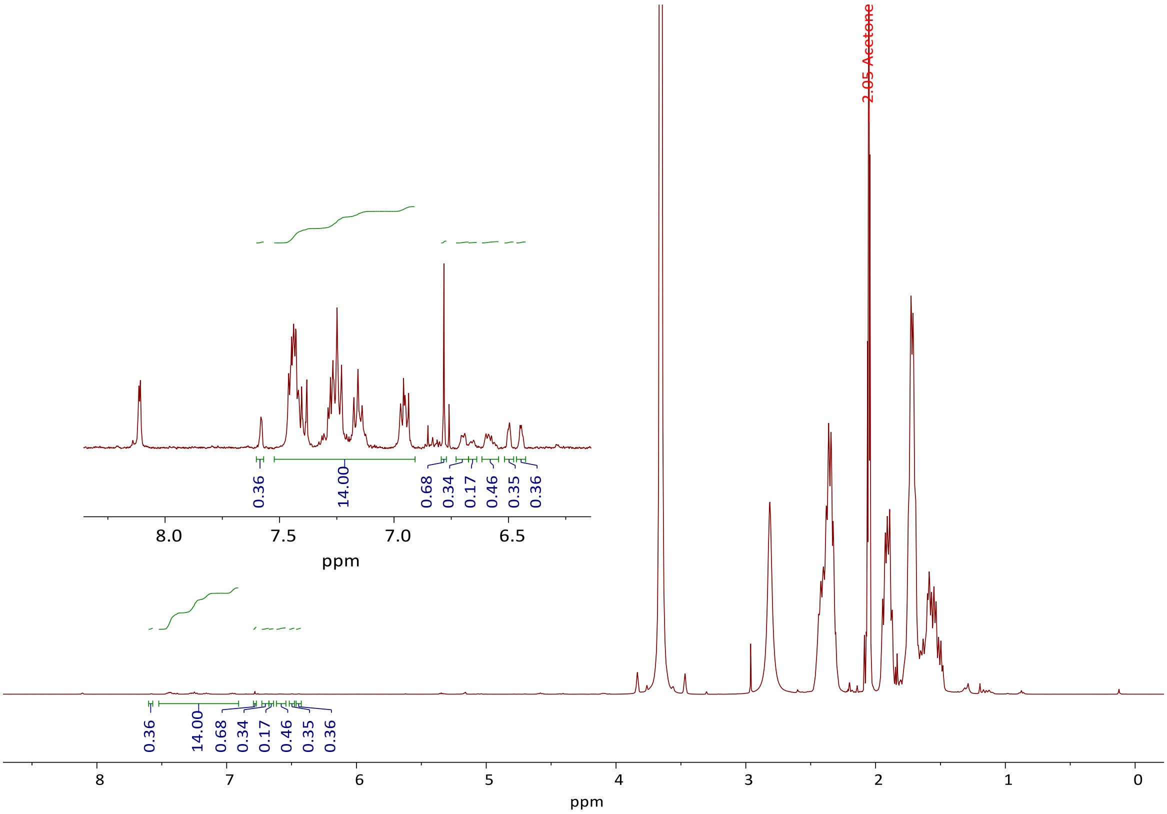

Spectrum S38. ^{1}H NMR (400 MHz, Acetone-d_6, 298 K) spectrum of post-sonication polymer **S17** before being washed with methanol.

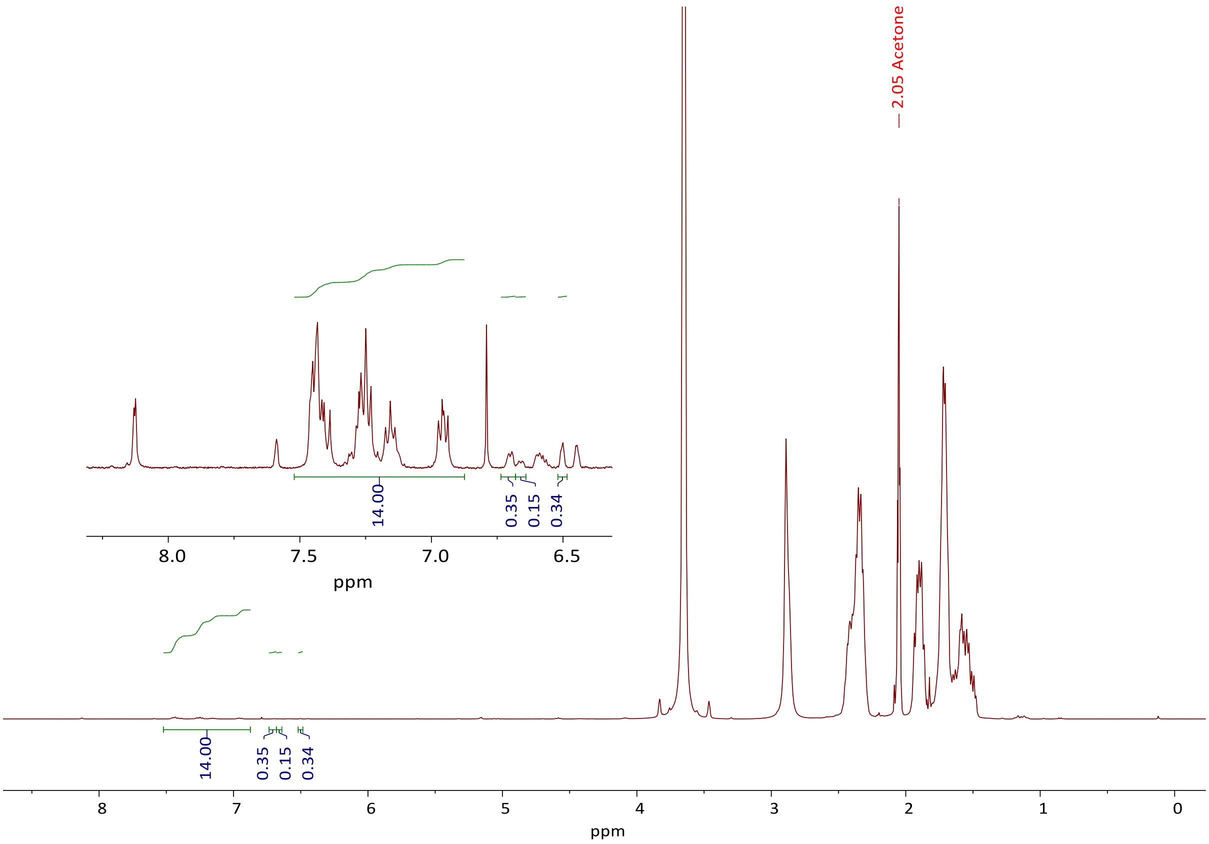

Spectrum S39. ^{1}H NMR (400 MHz, Acetone-d_6, 298 K) spectrum of post-sonication polymer **S17** after being washed with methanol.

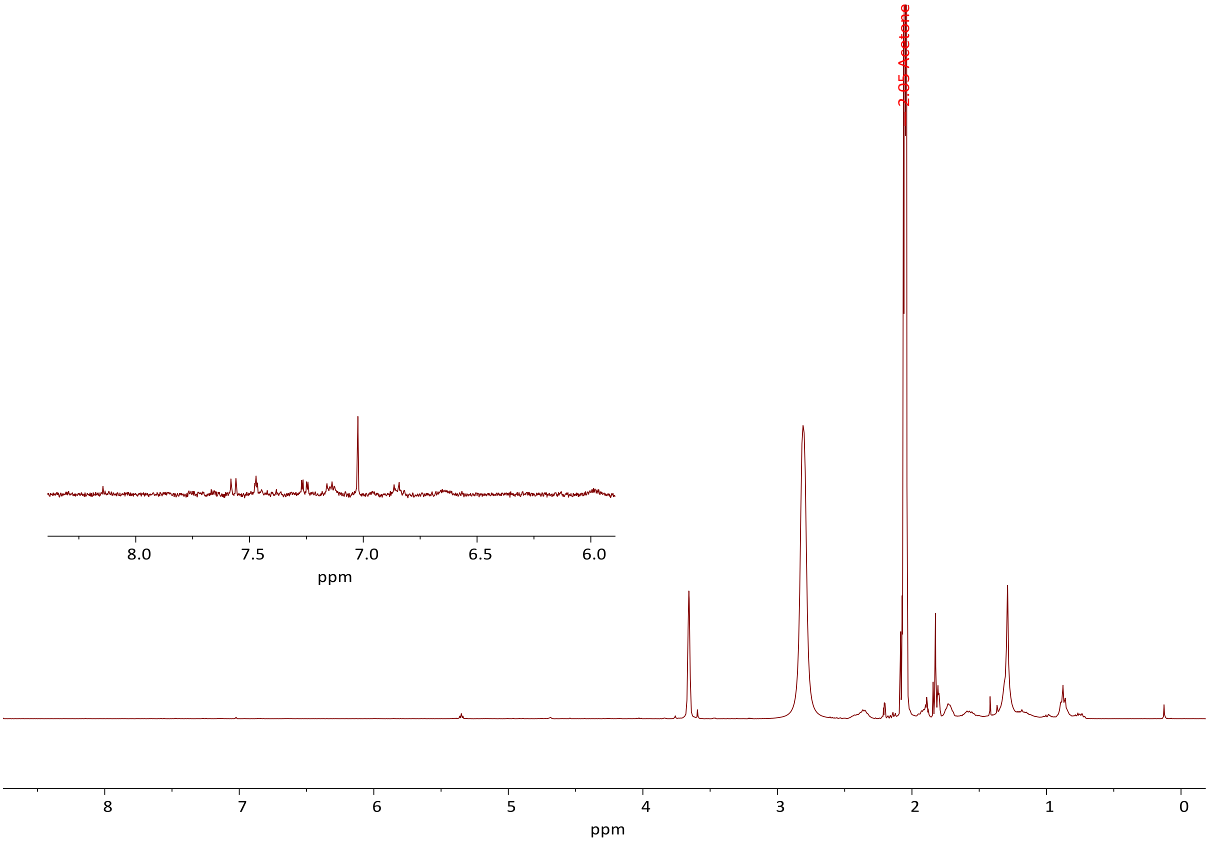

Spectrum S40. ^{1}H NMR (400 MHz, Acetone-d_6, 298 K) spectrum of the concentrated methanol washings from post-sonication polymer **S17**.

9 Mass Spectrometry Isotopic Patterns

9.1 Isotopic distribution of S5

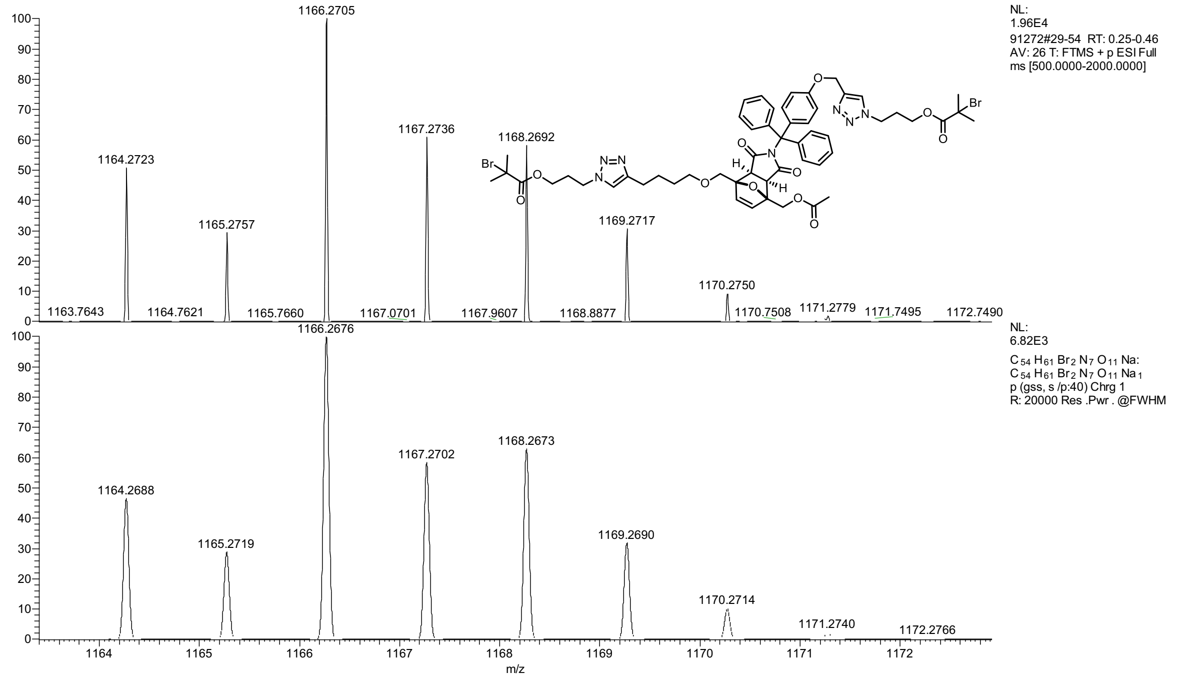

Spectrum S41. Isotopic distribution of **S5**. Top: Measured isotopic distribution for $C_{54}H_{61}Br_2N_7O_{11}Na$ ($[M+Na]^+$, +ESI). Bottom: Simulated isotopic distribution for $C_{54}H_{61}Br_2N_7O_{11}Na^+$.

9.2 Isotopic distribution of S7

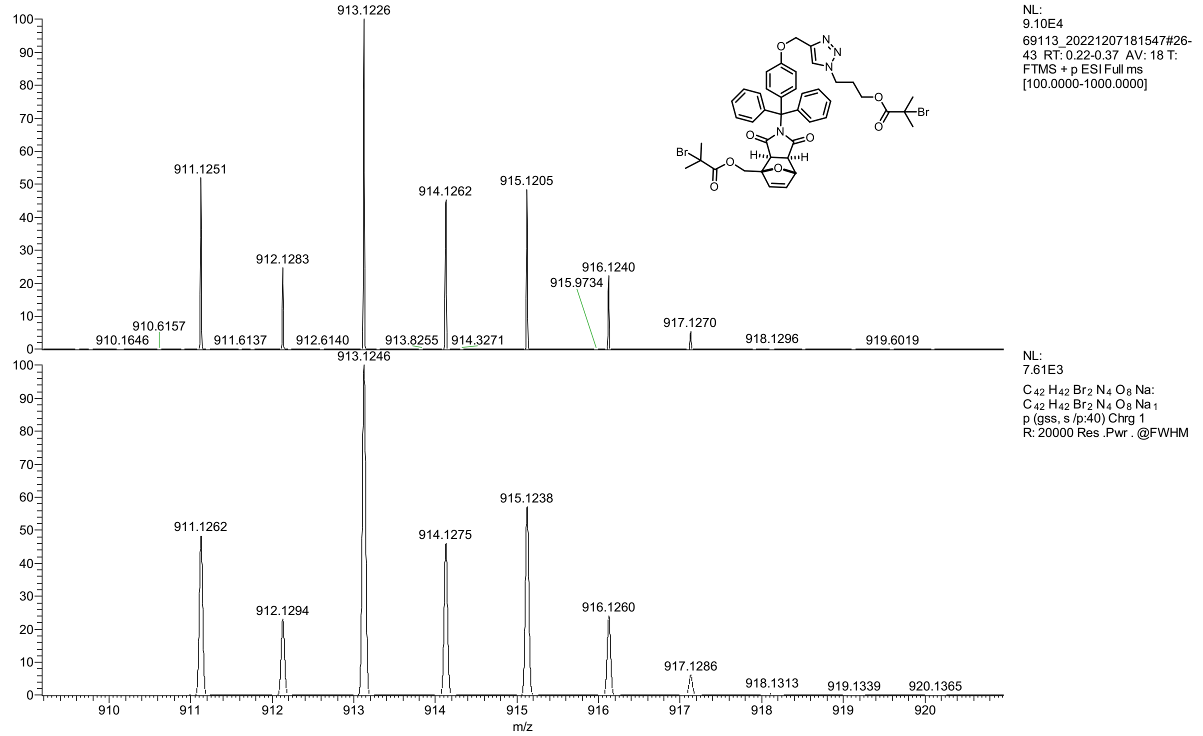

Spectrum S42. Isotopic distribution of **S7**. Top: Measured isotopic distribution for $C_{42}H_{42}Br_2N_4O_8Na$ ($[M+Na]^+$, +ESI). Bottom: Simulated isotopic distribution for $C_{42}H_{42}Br_2N_4O_8Na^+$.

10 References

(1) Antoni, P.; Hed, Y.; Nordberg, A.; Nyström, D.; von Holst, H.; Hult, A.; Malkoch, M. *Angew. Chem. Int. Ed.* **2009**, *48*, 2126-2130.

(2) Chen, L.; Nixon, R.; De Bo, G. *Nature* ***2024**, 628,* 320–325.

(3) Stevenson, R.; De Bo, G. *J. Am. Chem. Soc.* **2017**, *139*, 16768-16771.

(4) Hickenboth, C. R.; Moore, J. S.; White, S. R.; Sottos, N. R.; Baudry, J.; Wilson, S. R. *Nature* **2007**, *446*, 423-427.

(5) Sato, T.; Nalepa, D. E. *J. Appl. Polym. Sci.* **1978**, *22*, 865-867.

(6) Beyer, M. K. *J. Chem. Phys.* **2000**, *112*, 7307-7312.
